# PPARα activation overcomes fibroinflammatory liver microenvironment-associated anti-PD-1 resistance in hepatocellular carcinoma by mediating GSDME-dependent pyroptosis

**DOI:** 10.1038/s41467-026-75770-7

**Published:** 2026-08-06

**Authors:** Peng Chen, Kai Xiong, Kuiyuan Huang, Zheyu Dong, Junjie Liang, Xiaoning Gan, Tengzhen Li, Morang Zhang, Shunzi Jing, Xinwen Xu, Yalu Zheng, Zhangyun Li, Weicong Chen, Dandan Zheng, Yuanyuan Zhong, Guanqi Dai, Qiang Li, Jiaojiao Chu, Mingrong Cao, Jian Sun, Zhilong Liu, Jiexin Li, Shangxiang Chen, Yuanbo Zhao, Qin Juan, Xueqin Li, Zhili Wen, Yongyin Li, Huajin Pang, Duo Wang, Yuchuan Jiang

**Affiliations:** 1 Department of Gastroenterology, The Second Affiliated Hospital, Jiangxi Medical College, Nanchang University, Nanchang, Jiangxi China; 2 Department of Infectious Diseases, Nanfang Hospital, Southern Medical University, Guangzhou, China; 3 State Key Laboratory of Organ Failure Research, Key Laboratory of Infectious Diseases Research in South China, Ministry of Education, Guangdong Provincial Key Laboratory for Prevention and Control of Major Liver Diseases, Guangdong Provincial Clinical Research Center for Viral Hepatitis, Guangdong Institute of Hepatology, Guangdong Provincial Research Center for Liver Fibrosis Engineering and Technology, Guangzhou, China; 4 State Key Laboratory of Neurology and Oncology Drug Development, Nanjing, China; 5 Department of Hepatobiliary and Pancreatic Surgery, The First Affiliated Hospital of Jinan University, Jinan University, Guangzhou, China; 6 Department of Medical Oncology, The Second Affiliated Hospital, School of Medicine, South China University of Technology, Guangzhou, China; 7 Department of Breast Surgery, The Second People’s Hospital of Foshan, Foshan, China; 8 School of Traditional Chinese Medicine, Southern Medical University, Guangzhou, China; 9 Department of Radiation Oncology, The First Affiliated Hospital of Zhejiang University, Hangzhou, China; 10 Third Clinical Medical College of Ningxia Medical University, Ningxia Medical University, Yinchuan, China; 11 Department of General Surgery, The First Affiliated Hospital, Jiangxi Medical College, Nanchang University, Nanchang, China; 12 Zhujiang Hospital, Southern Medical University, Guangzhou, China; 13 Center of Interventional Radiology & Vascular Surgery, Department of Radiology, Zhongda Hospital, Medical School, Southeast University, Nanjing, China; 14 Interventional Therapy Center, Zhujiang Hospital, Southern Medical University, Guangzhou, China

**Keywords:** Cancer immunotherapy, Cancer microenvironment, Cancer microenvironment, Immunotherapy

## Abstract

The fibroinflammatory liver microenvironment (FILM), characterized by collagen-rich stroma and immunosuppressive inflammation, is prevalent in hepatocellular carcinoma (HCC) and correlates with poor response to programmed cell death protein 1 (PD-1) blockade. Here, we show that FILM suppresses gasdermin E (GSDME)-dependent pyroptosis and promotes immune suppression and anti-PD-1 resistance. Mechanistically, FILM-associated cancer-associated fibroblasts recruit and polarize macrophages toward a nitric oxide synthase 2 (NOS2)⁺ inflammatory phenotype. NOS2+ macrophage-derived nitric oxide induces SP1 S-nitrosylation, impairs SP1 binding to the peroxisome proliferator-activated receptor alpha (PPARA) promoter and transcriptionally represses PPARA in HCC cells. PPARα downregulation reduces pyruvate dehydrogenase kinase 4 (PDK4) expression, mitochondrial reactive oxygen species production, caspase-3 activation and GSDME cleavage. Conversely, ligand activation of tumor intrinsic PPARα restores the PDK4–ROS–caspase-3–GSDME axis, enhances dendritic cell and CD8⁺ T cell activation, and sensitizes HCC to anti–PD-1 therapy. The clinically approved PPARα agonist fenofibrate enhances anti-PD-1 efficacy in HCC models in male mice and is associated with improved clinical benefit in a retrospective cohort of patients with HCC. We propose a FILM–NOS2–SP1–PPARα–PDK4 axis that controls pyroptotic immunogenicity and immunotherapy response, supporting PPARα activation as a strategy to overcome FILM-associated immune resistance in HCC.

## Abstract (web summary)

A fibroinflammatory liver microenvironment (FILM) is prevalent in hepatocellular carcinoma (HCC) and associated with anti-PD-1 resistance. Here the authors show that FILM suppresses GSDME-dependent pyroptosis and drives immune suppression, proposing PPARα activation as a strategy to overcome FILM-associated immune resistance in HCC.

## Introduction

Most patients with hepatocellular carcinoma (HCC) are diagnosed at an advanced-stage, limiting therapeutic options to non-curative local or systemic interventions^1^. Immune checkpoint inhibitors (ICIs), particularly programmed death-1 (PD-1) monoclonal antibody (mAbs), have revolutionized the management of advanced HCC^2^. Although the CheckMate-040 and KEYNOTE-224 trials demonstrated that PD-1 mAb monotherapy extends median overall survival beyond 12.9 months, objective response rates (ORR) remain modest^3–6^. Recent trials, including IMbrave150 and CARES-310, combining ICIs with anti-angiogenic agents increased ORR to ~30%^7–9^, highlighting immunotherapy resistance as a central challenge and suggesting combination therapies may represent a viable solution.

As a paradigm of inflammation-driven malignancy, HCC develops in 90% of cases from chronic liver diseases, including viral hepatitis, alcohol-associated liver disease, and non-alcoholic fatty liver disease^10^. The progression of these conditions triggers a pathogenic shift toward hepatic fibro-inflammation, characterized by self-reinforcing interactions between fibrotic remodeling and chronic inflammatory cascades^11,12^. Within the fibroinflammatory liver microenvironment (FILM), dysregulated immune homeostasis coexists with hepatic and intratumoral immunosuppressive niches, collectively promoting therapeutic resistance^11,12^. For instance, non-alcoholic steatohepatitis (NASH) subverts PD-1 mAb efficacy via chronic inflammation-driven nonspecific activation of CD8^+^ PD-1^+^ T cells. This aberrant activation paradoxically promotes both pathological T cell accumulation and exhaustion, thereby disabling their tumor-targeting capacity and sustaining immunotherapy resistance^13^. Moreover, diverse etiologies of hepatic fibroinflammation elicit common immunosuppressive pathways, including hepatic stellate cell (HSC)-mediated reprogramming of myeloid-derived suppressor cells (MDSCs) and M2 macrophage polarization, which attenuate T-cells effector functions and ICI responses^14,15^. Therefore, elucidating the mechanisms underlying FILM-driven immunosuppression in HCC, along with strategies to enhance tumor-specific T cell activation, represent a pivotal avenue for improving immunotherapy efficacy.

Pyroptosis is a form of immunogenic cell death (ICD) mediated by gasdermin (GSDM) proteins and is triggered by proteolytic cleavage that releases the GSDM N‑terminal fragment. These fragments oligomerize to form membrane pores that promote the release of inflammatory mediators and drive immune activation^16^. Gasdermin E (GSDME)-mediated pyroptosis is considered a prerequisite for effective tumor immunotherapy, as GSDME pore formation exposes tumor antigens and releases danger-associated molecular patterns such as calreticulin (CRT) and high-mobility group box 1 (HMGB1), thereby recruiting immune cells, priming dendritic cells, and activating tumor-specific T cells^17–19^. Within an immunologically infiltrated tumor microenvironment (TME), PD-1 mAb therapy potentiates effector T-cell release of perforin (PFN) and granzyme B (GzmB), which proteolytically activate GSDME. This GSDME-driven pyroptosis subsequently recruits additional immune cells, creating an antitumor immunity-amplifying loop^18,20,21^. Notably, HCC retains preserved GSDME expression compared to other malignancies, suggesting therapeutic potential for pyroptosis induction^22,23^.

Peroxisome proliferator-activated receptor alpha (PPARA), a core regulatory member of the nuclear receptor superfamily, exerts its functions through ligand-dependent activation and governs physiological processes including lipid metabolism, inflammatory regulation, and cellular proliferation and differentiation^24^. Emerging evidence suggests that PPARα plays complex and context-dependent roles in tumor biology, including immune evasion, stemness maintenance, and angiogenesis^25,26^. In HCC, these effects appear particularly paradoxical^27^. Multiple studies demonstrate its tumor-promoting capacity through iron-dependent signaling pathways, SCD1-mediated chemoresistance via lipid metabolic reprogramming, and Wnt5b-Fzd5-β-catenin-driven expansion of liver cancer stem cells^28,29^. Conversely, PPARα activation has been implicated in suppressing HCC progression by reducing neutrophil extracellular trap formation, ameliorating hepatic lipid accumulation, and attenuating chronic inflammatory responses^30,31^. These findings suggest that the immune microenvironment may critically shape the functional role of PPARα in HCC, yet the interplay between PPARα and HCC immunotherapy remains unexplored.

Here, we show that FILM suppresses GSDME-dependent pyroptosis and limits anti-PD-1 efficacy by reducing tumor-cell PPARA expression. FILM-associated cancer-associated fibroblasts recruit and polarize NOS2⁺ macrophages, whose nitric oxide induces SP1 S-nitrosylation and reduces SP1 occupancy at the PPARA promoter. Ligand-dependent activation of tumor-cell-intrinsic PPARα transactivates PDK4 and promotes fatty-acid oxidation-associated mitochondrial reactive oxygen species accumulation, caspase-3 activation and GSDME cleavage, thereby enhancing dendritic-cell and CD8⁺ T-cell responses. Genetic and pharmacological studies in HCC models, together with exploratory clinical analyses, support prospective evaluation of PPARα agonists in PPARα-retaining HCC.

## Results

### FILM promotes anti-PD-1 resistance in HCC by inhibiting GSDME-mediated pyroptosis

**Fig. 1:**
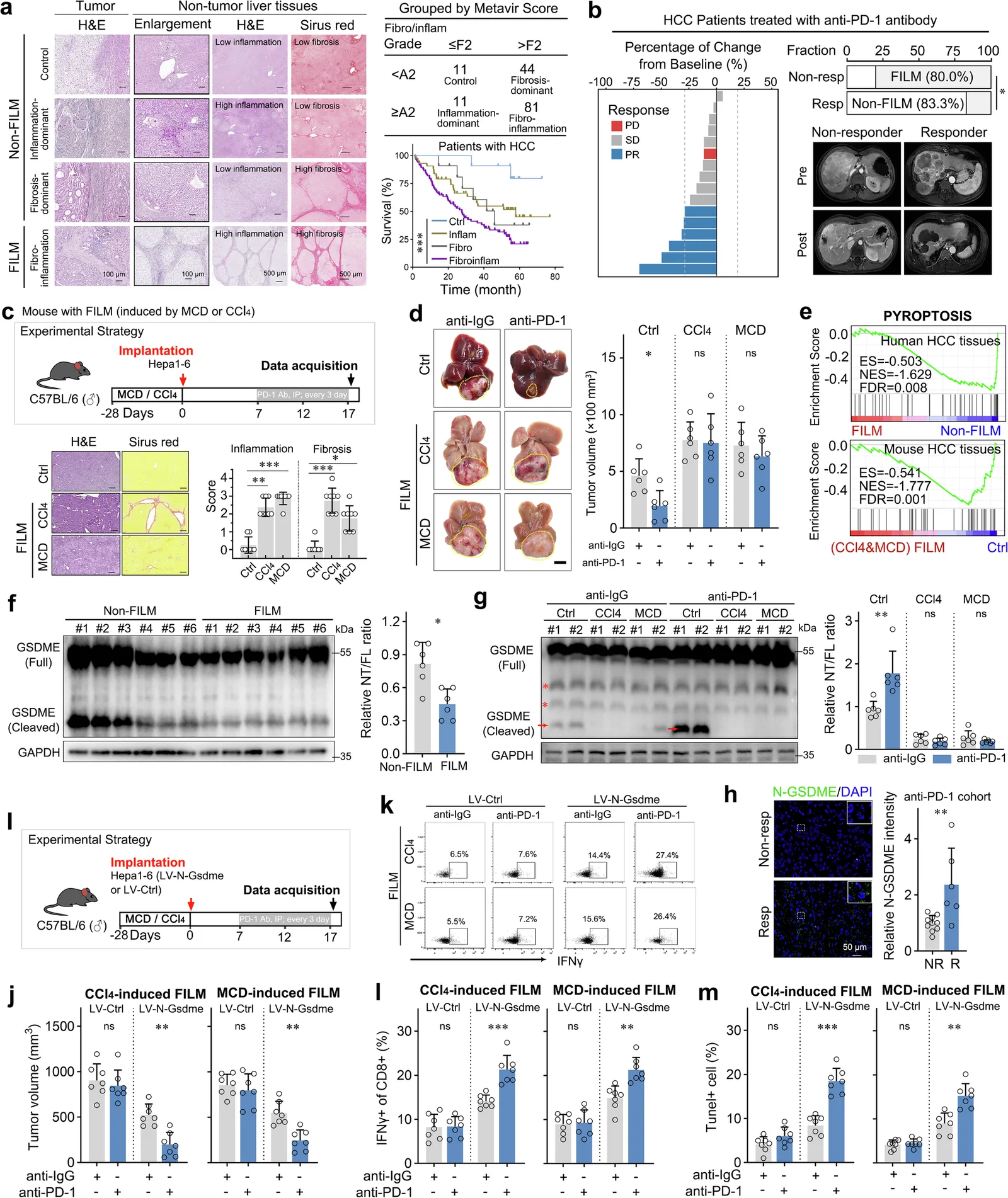
FILM promotes anti-PD-1 resistance in HCC by inhibiting GSDME-mediated pyroptosis and immune activation. **a** Representative H&E and Sirius Red staining of HCC and adjacent liver tissues from control (*n* = 11), inflammation-dominant (*n* = 11), fibrosis-dominant (*n* = 44) and FILM (*n* = 81) groups; classification of 147 patients by METAVIR inflammation and fibrosis scores; and Kaplan–Meier analysis of treatment-naive patients according to FILM status. **b** Distribution of anti-PD-1 responders (*n* = 6) and non-responders (*n* = 10) in FILM and non-FILM HCC, with representative MRI images. **c** Experimental scheme for CCl4- and MCD-induced FILM orthotopic HCC models, representative H&E and Sirius Red staining, and inflammation and fibrosis scores (*n* = 8 mice per group), Scale bar, 100 μm. **d** Representative gross liver images and tumor volumes (*n* = 6 mice per group), Scale bar, 5 mm. **e** Gene-set enrichment analysis of cell death-related pathways showing suppression of pyroptosis-associated signatures in FILM HCC compared with control HCC. The datasets and sample numbers are indicated in the corresponding panels. **f** Immunoblot and quantification of the GSDME-N/full-length GSDME ratio in non-FILM and FILM human HCC tissues (*n* = 6). **g** Immunoblot and quantification of GSDME cleavage in mouse HCC tissues (*n* = 6); the arrow indicates GSDME-N and the asterisk indicates a nonspecific band. **h** N-GSDME immunofluorescence in anti-PD-1 responders (*n* = 6) and non-responders (*n* = 10), Scale bar, 50 μm. **i** Experimental scheme for LV-Ctrl or LV-N-Gsdme implantation followed by control IgG or anti-PD-1 treatment. **j** Tumor volumes (*n* = 7 mice per group). **k** Representative flow-cytometry plots of IFNγ⁺ CD8⁺ T cells. **l**–**m** Frequencies of IFNγ⁺ CD8⁺ T cells and TUNEL⁺ cells (*n* = 7 mice per group). Data are mean ± s.d.; each point represents one independent patient or mouse. ns, not significant, *P < 0.05, **P < 0.01, ***P < 0.001. Exact *P* values are provided in Source Data files. Statistical significance was assessed by two-sided log-rank Mantel–Cox test in (**a**), two-sided Pearson χ² test in (**b**), two-sided Mann–Whitney U tests in (**f**, **h**) or Kruskal–Wallis tests in (**c**–**e**, **g**, **j**–**m**). FILM fibroinflammatory liver microenvironment, CCl_4_ carbon tetrachloride, MCD methionine- and choline-deficient, IgG immunoglobulin G, LV lentiviral vector, IFNγ interferon-γ, GZMB granzyme B, TUNEL terminal deoxynucleotidyl transferase dUTP nick-end labeling. Source data are provided as a Source Data file.

To explore the clinical significance of fibroinflammatory status in HCC, we retrospectively analyzed 147 surgically resected cases. Based on METAVIR scores of nontumorous liver tissue, patients were classified into four groups: control, inflammation-dominant, fibrosis-dominant and fibroinflammatory liver microenvironment (FILM), the latter characterized by concurrent marked fibrosis and active inflammation. More than half of the cases fell into the FILM group, whereas only 7.5% belonged to the control group (Fig. 1a, Supplementary Table 1). Kaplan–Meier analysis revealed that patients with FILM HCC had the poorest prognosis (Fig. 1a). We also retrospectively evaluated a cohort of patients who received anti–PD‑1 therapy prior to surgical resection. In this cohort, all of whom had advanced fibrosis, non-responders had significantly higher liver inflammation scores. This suggests that the FILM phenotype was more prevalent among non-responders than responders (Fig. 1b; Supplementary Fig. 1a). Additionally, to model this phenotype experimentally, we induced FILM in mice using carbon tetrachloride (CCl₄) or methionine–choline–deficient (MCD) diets, which resulted in pronounced liver fibrosis and inflammation (Fig. 1c; Supplementary Fig. 1b). Tumors implanted in FILM were completely resistant to anti–PD–1 therapy, whereas tumors in control livers responded effectively (Fig. 1d; Supplementary Fig. 1b, c). Furthermore, RNA sequencing revealed a broad downregulation of immune response–related genes in FILM HCC, and flow cytometry confirmed impaired CD8⁺ T-cell effector function, as evidenced by reduced interferon gamma (IFN-γ) and GzmB production, in both human and mouse HCC tissues (Supplementary Fig. 1d–h).

Interestingly, FILM tumors showed reduced TUNEL-positive area together with decreased membrane CRT and cytoplasmic HMGB1, suggesting an overall alteration in tumor cell death with reduced immunogenicity (Supplementary Fig. 2a). Given that PD-1 blockade is favored by a sufficiently immunogenic tumor microenvironment, we next examined immunogenic cell-death programs. Gene set enrichment analysis (GSEA) of cell death-related gene sets revealed significant suppression of the pyroptosis pathway in FILM, a pattern consistently observed in both mouse and human HCC tissues (Fig. 1e; Supplementary Fig. 2b–e). As pyroptosis is primarily mediated by the gasdermin family, we found that FILM did not significantly affect the expression of total GSDME, but markedly reduced its cleavage; by contrast, cleavage of other gasdermins showed no changes (Fig. 1f; Supplementary Fig. 2f, g). Consistent with a selective effect on pyroptotic execution, cleaved PARP1 and ferroptosis-related markers, including Gpx4, Acsl4 and Slc7a11, were not significantly decreased in FILM HCC compared with control tumors (Supplementary Fig. 2b, c), arguing against preferential suppression of apoptosis or ferroptosis in this setting. Importantly, although anti-PD‑1 therapy slightly increased GSDME expression in HCC implanted in both control livers and FILM, enhanced GSDME cleavage was observed only in tumors implanted in control livers, but not in FILM tumor (Fig. 1g). Clinically, HCC tissues from anti-PD‑1 responders exhibited significantly higher GSDME cleavage than in non‑responders (Fig. 1h). Functionally, Gsdme knockout promoted tumor growth and significantly impaired anti-PD-1 efficacy in mice with control livers (Supplementary Fig. 3a–c). Conversely, cleaved Gsdme overexpression provided only a modest incremental benefit in tumors implanted in control livers, where anti-PD-1 monotherapy is already effective (Supplementary Fig. 3d–f), but robustly restored anti-PD-1 responsiveness in FILM tumors (Fig. 1i, j), accompanied by an immune‑activated tumor microenvironment and an increase in TUNEL^+^ and 7‑AAD⁺/Annexin V⁺/CD45⁻ cells (Fig. 1k–m; Supplementary Fig. 4a–h). Notably, the deletion of CD8⁺ T cells or dendritic cells (DCs), but not other immune cell subsets, attenuated the sensitizing effect of GSDME cleavage on anti-PD‑1 therapy (Supplementary Fig. 4i). Collectively, these findings support a model in which FILM drives anti-PD-1 resistance in HCC by suppressing GSDME-dependent pyroptotic cell death.

### FILM-associated PPARα downregulation suppresses GSDME-mediated pyroptosis and drives anti-PD-1 resistance

**Fig. 2:**
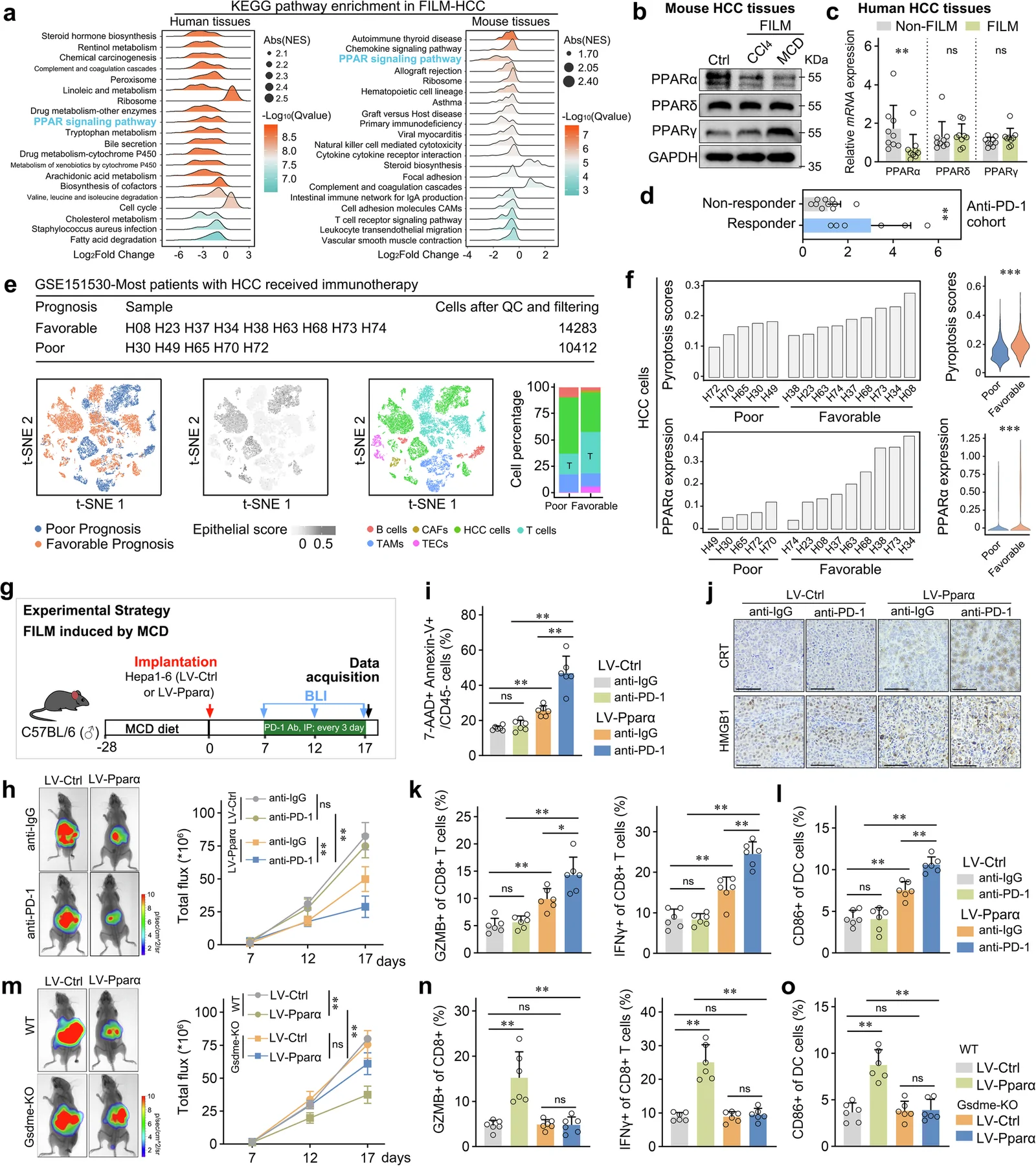
PPARA overexpression promotes pyroptosis and overcomes anti-PD-1 resistance of HCC in FILM. **a** KEGG pathway enrichment in FILM versus non-FILM HCC of human (FILM, *n* = 9, non-FILM, *n* = 9) and mouse (FILM, *n* = 6, non-FILM, *n* = 3); PPAR signaling is highlighted. **b** Immunoblot analysis of PPARα, PPARδ and PPARγ in mouse HCC tissues (Representative blots from *n* = 3 biologically independent experiments). **c** PPARA, PPARD and PPARG mRNA expression in non-FILM and FILM human HCC tissues (*n* = 9 patients per group). **d** PPARA expression in anti-PD-1 responders (*n* = 6) and non-responders (*n* = 10). **e** Overview of GSE151530, including prognostic groups, sample IDs, cell numbers after quality control, t-SNE distributions and cell-type proportions. **f** Pyroptosis scores and PPARA expression in malignant cells stratified by prognosis. **g** Experimental scheme for LV-Ctrl or LV-Ppara implantation in MCD-induced FILM-HCC followed by control IgG or anti-PD-1 treatment. **h** Representative bioluminescence images and radiance (*n* = 6 mice per group). **i** Annexin V + 7-AAD + CD45− terminally membrane-disrupted tumor cells quantified by flow cytometry (*n* = 6 mice per group). **j** Representative CRT and HMGB1 IHC and quantification of membrane CRT and cytoplasmic HMGB1 scores (*n* = 6 mice per group). Scale bar, 100 μm. **k** GZMB+ and IFNγ + CD8 + T cells. **l** CD86+ dendritic cells. **m** Bioluminescence and radiance in WT and GSDME-KO tumors with or without Ppara overexpression under control IgG or anti-PD-1 treatment (*n* = 6 mice per group). **n**, **o** GZMB+ and IFNγ + CD8 + T cells and CD86+ dendritic cells in the indicated groups. Data are mean ± s.d.; each point represents one independent biological experiment, mouse or patient as indicated (*n* = 6 mice per group). ns, not significant, *P < 0.05, **P < 0.01, ***P < 0.001. Exact *P* values are provided in Source Data files. Statistical significance was assessed using two-sided Mann–Whitney U tests in (**c**, **d** and **f**), and Kruskal–Wallis tests in (**h**, **i** and **k**–**o**). KEGG Kyoto Encyclopedia of Genes and Genomes, PPAR peroxisome proliferator-activated receptor, QC quality control, t-SNE t-distributed stochastic neighbor embedding, CRT calreticulin, HMGB1 high-mobility group box 1, WT wild type, KO knockout, DC dendritic cell. Source data are provided as a Source Data file.

Given that FILM suppressed pyroptosis and contributed to anti–PD‑1 resistance, we next performed KEGG-based GSEA to identify conserved signaling pathways altered in FILM HCC. Among the altered pathways, PPAR signaling was markedly downregulated in both human and mouse FILM HCC compared with non‑FILM or control counterparts (Fig. 2a). Consistently, FILM selectively suppressed PPARα expression and its corresponding pathway activation in HCC, whereas PPARγ and PPARδ expression remained unaffected (Fig. 2b, c; Supplementary Fig. 5a). In the anti-PD-1 cohort, *PPARA* mRNA levels were significantly downregulated in HCC from non-responders compared with responders (Fig. 2d). Additionally, single-cell RNA-sequencing data from immunotherapy-treated HCC patients revealed that better prognoses are associated with greater cytotoxic T-cell infiltration, higher pyroptosis scores, and specifically elevated PPARα expression and pathway enrichment in HCC cells, whereas such associations were absent for PPARγ and PPARδ (Fig. 2e, f; Supplementary Fig. 5b–k). Consistently, bulk RNA-sequencing datasets from TCGA, ICGC, and CPTAC similarly showed that high PPARα expression predicted improved overall survival (Supplementary Fig. 6a).

To delineate whether PPARα regulates tumor pyroptosis and anti-PD-1 resistance in FILM, we orthotopically implanted *Ppara*-overexpressing Hepa1-6 cells into FILM mouse models (Fig. 2g, Supplementary Fig. 6b). *Ppara* overexpression markedly suppressed tumor growth and promoted ICD, as indicated by increased tumor-cell membrane permeabilization (7‑AAD⁺/Annexin V⁺/CD45⁻) together with elevated levels of membrane CRT and cytoplasmic HMGB1 in vivo (Fig. 2h–j; Supplementary Fig. 6c–f). Notably, PPARA overexpression specifically promoted GSDME cleavage without altering the other gasdermins (Supplementary Fig. 6g), and reversed FILM-associated anti–PD-1 resistance, as indicated by suppressed tumor growth and increased infiltration of activated DCs and cytotoxic CD8⁺ T cells (Fig. 2h–l; Supplementary Fig. 6h–i).

To confirm that GSDME cleavage is essential for PPARα’s effects, we knocked out Gsdme in Hepa1-6 cells (Supplementary Fig. 3a). We found that loss of Gsdme abrogated the ability of PPARA overexpression to sensitize FILM tumors to anti–PD-1 therapy and significantly reduced terminal membrane-disrupted tumor cells (7‑AAD⁺/Annexin V⁺/CD45⁻), as well as the immune-reprogramming effects within the tumor microenvironment (Fig. 2m–o). Collectively, these findings demonstrate that PPARα downregulation in FILM HCC suppresses GSDME-dependent pyroptosis, thereby promoting resistance to anti–PD-1 therapy.

### FILM suppresses PPARA expression via an NOS2+macrophage-NO-SNO-SP1 axis

**Fig. 3:**
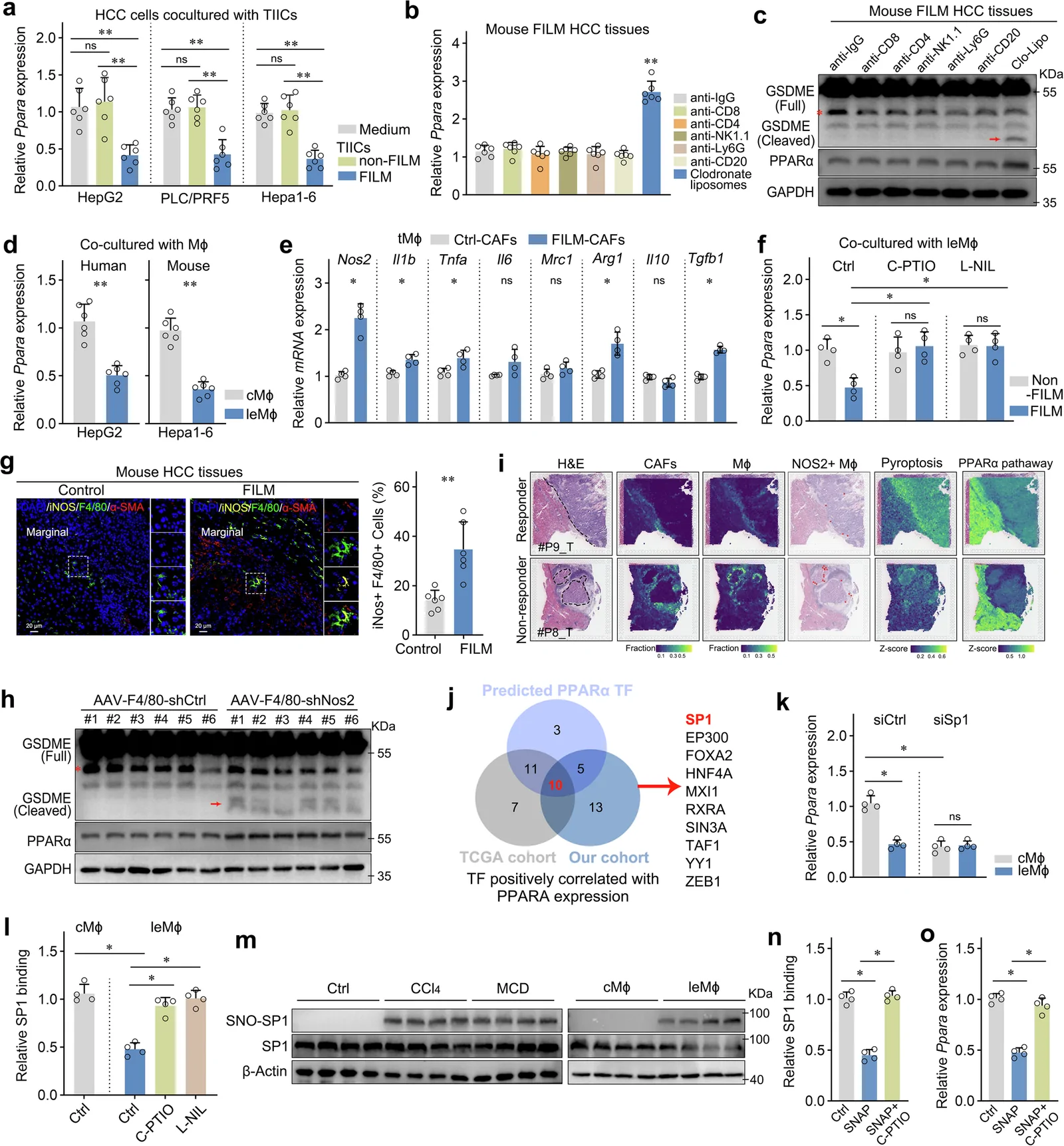
FILM-associated NOS2^+^ macrophages suppress tumor-cell PPARA through NO-dependent SP1 S-nitrosylation. **a** PPARA expression in HepG2, PLC/PRF/5 and Hepa1-6 cells co-cultured with tumor-infiltrating immune cells from non-FILM or FILM HCC (*n* = 6 independent experiments). **b** Ppara expression in MCD-induced FILM tumors after the indicated immune-cell depletion treatments (*n* = 6 mice per group). **c** Immunoblot analysis of GSDME cleavage and PPARα after immune-cell depletion (*n* = 6 mice per group); arrows indicate GSDME-N and asterisks indicate nonspecific bands. **d** PPARA expression in HCC cells co-cultured with leading-edge (le) or core (c) macrophages from human or mouse FILM-HCC (*n* = 6 independent experiments). **e** Expression of inflammatory, M2-associated and TAM-associated genes in macrophages polarized by control or FILM-derived CAFs (*n* = 4 independent experiments). **f** Ppara expression after co-culture with le macrophages from non-FILM or FILM tumors with or without C-PTIO or L-NIL (*n* = 4 independent experiments). **g** Multiplex immunofluorescence of iNOS, F4/80, α-SMA and DAPI and quantification of iNOS⁺F4/80⁺ macrophages at tumor margins (*n* = 6 mice per group). **h** Immunoblot analysis of GSDME cleavage, PPARα and GAPDH in tumors from AAV8-F4/80-shCtrl- or AAV8-F4/80-shNos2-treated mice (*n* = 6). **i** Spatial distributions of CAFs, macrophages, NOS2, pyroptosis activity and PPARα activity in representative anti-PD-1 responder and non-responder HCC tissues; color scales are defined in-panel, representative sections from one responder and one non-responder are shown. **j** Integrated analysis of predicted PPARA transcription factors and their correlation with PPARA expression in TCGA-LIHC (*n* = 364) and our HCC cohort (*n* = 20), identifying SP1 as a candidate regulator. **k** PPARA expression after siSP1 and macrophage co-culture. **l**, **n** SP1 ChIP–qPCR at the PPARA promoter after macrophage co-culture, C-PTIO, L-NIL or SNAP treatment. **m** Biotin-switch analysis of SNO-SP1 and total SP1. **o** PPARA expression after SNAP treatment (**k**–**o**, *n* = 4 independent experiments or mice, as indicated). Data are mean ± s.d.; each point represents one independent experiment or mouse. ns, not significant, *P < 0.05, **P < 0.01, ***P < 0.001. Exact *P* values are provided in Source Data files. Statistical significance was assessed using two-sided Mann–Whitney U tests in (**d**, **g**, **l**, **m** and **n**) and Kruskal–Wallis tests in (**a**–**c**, **f**, **i**, **j** and **k**). Source data are provided as a Source Data file.

Since PPARα expression is critical for the reversal of FILM‑associated anti‑PD‑1 resistance, we next sought to investigate how FILM suppressed PPARα expression. To this end, the effects of tumor microenvironment components from HCC implanted in FILM or control livers on PPARα expression were compared. While cancer associated fibroblasts (CAFs) from both sources exhibited minimal impact on PPARα levels (Supplementary Fig. 7a), tumor‑infiltrating immune cells (TIICs) isolated from FILM HCC significantly suppressed PPARα expression compared to those from HCC implanted in control livers (Fig. 3a). Furthermore, depletion of macrophages using clodronate liposomes or anti-CSF1R, but not the other immune subsets, restored PPARα expression and increased GSDME cleavage, indicating macrophages as the key suppressors (Fig. 3b, c; Supplementary Fig. 7b, c). Interestingly, only leading edge-derived macrophages (leMφ) from FILM tumors, rather than core-derived macrophages (cMφ), downregulated PPARα in co-cultured HCC cells (Fig. 3d). In light of the potential for cell-cell interactions and the features of FILM, we performed dual immunostaining, which revealed that both ECM and macrophages were predominantly enriched at the leading edge of FILM tumors (Supplementary Fig. 7d, e). Transwell assays revealed that FILM‑derived CAFs strongly recruited bone marrow‑derived macrophages (BMDMs) and polarized them toward a phenotype that co-expressed M1-associated genes (Nos2, Il1b, Tnfa) together with selected M2 markers (e.g., Arg1), consistent with a protumoral, inflammatory TAM-like state (Fig. 3e; Supplementary Fig. 7f, g); notably, leading edge-derived macrophages directly isolated from FILM tumors also displayed a similar phenotype compared with control counterparts (Supplementary Fig. 7h).

Furthermore, NO scavenger (C-PTIO) or NOS2 inhibitor (NIL) but not anti-Il1β or anti-Tnfα restored *Ppara* expression in HCC cells co‑cultured with leading edge-derived macrophages from FILM tumors or bone marrow‑derived macrophages polarized by FILM‑tumor‑derived CAFs, but not in those co-cultured with control liver-derived macrophages or with BMDMs polarized by normal-liver-derived CAFs (Fig. 3f; Supplementary Fig. 7i–k). Consistently, immunofluorescence revealed that NOS2⁺ macrophages were markedly enriched in FILM compared with non-FILM HCC, and associated with anti-PD-1 resistance in the human HCC cohort (Fig. 3g; Supplementary Fig. 7l, m). Specific inhibition of NOS2 in macrophages using AAV8 vectors (Supplementary Fig. 8) significantly inhibited tumor growth, prolonged survival, restored PPARα expression and GSDME cleavage in FILM HCC mouse models, and further enhanced the anti‑tumor efficacy of either anti‑PD‑1 (Fig. 3h; Supplementary Fig. 9a, b). Spatial transcriptomic analysis of HCC tissues from patients treated with anti-PD‑1 revealed that non‑responders harbored a leading edge niche enriched in CAFs, macrophages, and NOS2⁺ macrophages, accompanied by suppressed PPARα signaling and pyroptosis pathways compared to responders (Fig.3i).

To delineate how NO suppressed PPARα expression, we focused on the transcription factor (TF) predicted to regulate the PPARA promoter. The hTFtarget database predicted 29 TFs targeting PPARA; among them, only 10 showed a consistent positive correlation with PPARA expression in both TCGA and our HCC cohorts (Fig. 3j). We then performed an siRNA screen and found that knockdown of Sp1, but not the other candidate TFs, markedly reduced Ppara expression across macrophage co-culture conditions, indicating that SP1 is required to sustain Ppara transcription in HCC cells (Fig. 3k; Supplementary Fig. 9c). Notably, co-culture with leMφ had no significant effect on SP1 expression in HCC cells (Supplementary Fig. 9d), yet it markedly reduced SP1 occupancy at the PPARA promoter, and this effect was reversed by C-PTIO or NIL (Fig. 3l). Given that NO affected SP1 promoter binding without changing SP1 expression, we hypothesized that NO impairs SP1 transcriptional activity via a post-translational modification. Consistent with this notion, we found that SP1 s-nitrosylation (SNO-SP1) was significantly increased in FILM HCC tissues and in HCC cells cocultured with FILM leMφ, compared to their respective controls (Fig. 3m). Treatment of HCC cells with SNAP, an NO donor, reduced SP1 occupancy at the PPARA promoter and decreased *PPARA mRNA* expression (Fig. 3n, o). These effects were reversed by C-PTIO, supporting a direct NO-dependent mechanism (Fig. 3n, o). Collectively, these data indicate that FILM leMφ-derived NO suppresses PPARA transcription by inducing SP1 S-nitrosylation, thereby impairing SP1 promoter occupancy and transcriptional activity.

### Ligand-activated PPARα induces GSDME-dependent pyroptosis through an FAO–mitochondrial ROS/caspase-3 axis

**Fig. 4:**
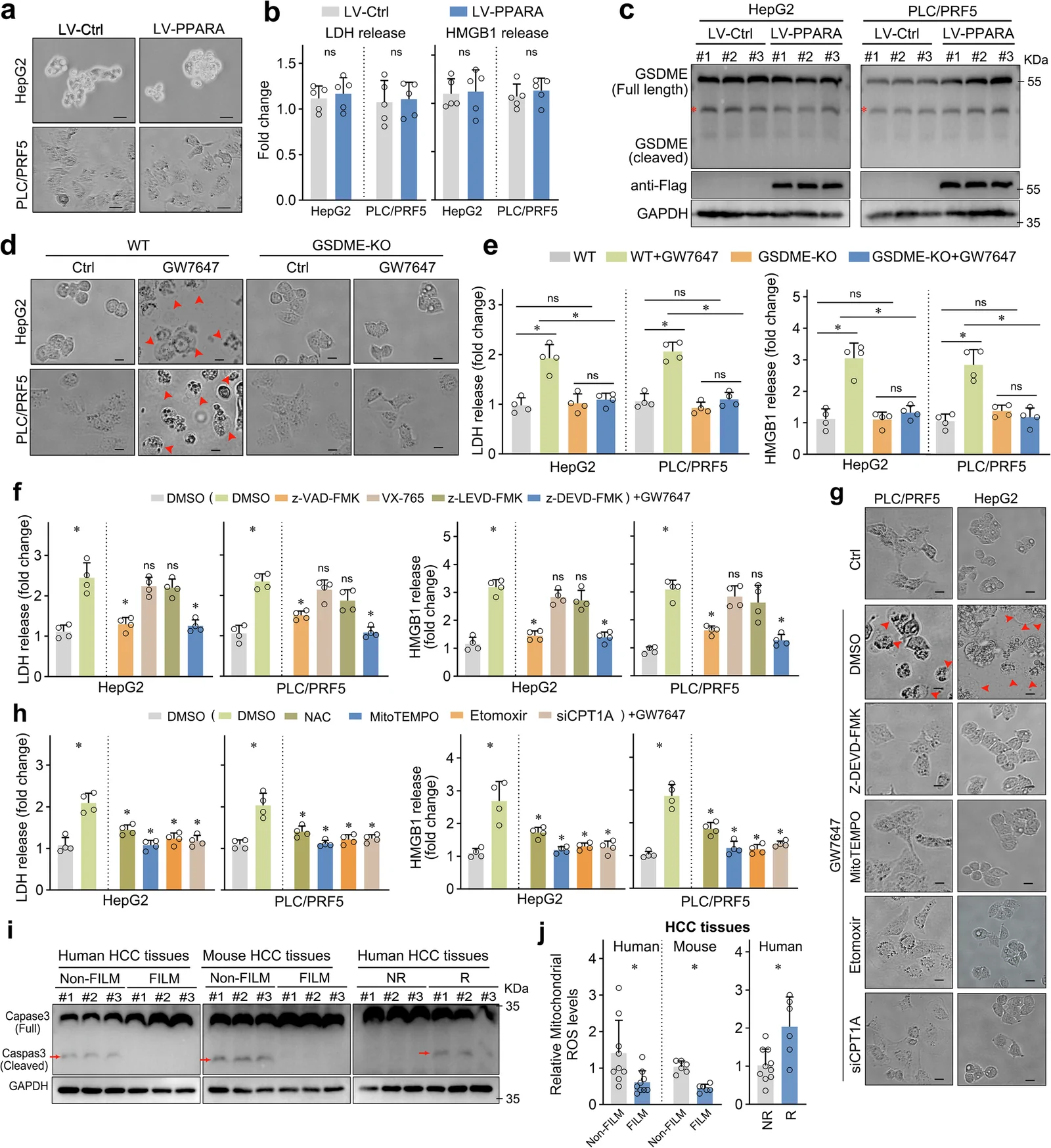
Ligand-dependent PPARα activation promotes GSDME-mediated pyroptosis via canonical ROS-caspase3 pathway. **a** Representative microscopic images of HepG2 and PLC/PRF5 cells transduced with LV-Ctrl or LV-PPARA. *n* = 4 independent experiments. Scale bars, 20 μm. **b** Relative LDH and HMGB1 levels in the supernatant of HepG2 and PLC/PRF/5 cells transduced with LV-Ctrl or LV-PPARA, *n* = 5 independent experiments. **c** Western blot analysis of full-length and cleaved GSDME, Flag-tagged PPARα in HepG2 and PLC/PRF/5 cells transduced with LV-Ctrl or LV-PPARα, *n* = 3 independent experiments, the red asterisk marked a nonspecific band. **d** Representative microscopic images of WT or GSDME-KO HepG2 and PLC/PRF5 cells treated with vehicle or 20 μM GW7647, and red arrows indicated pyroptotic cells, *n* = 4 independent experiments. Scale bars, 20 μm. **e** Relative LDH and HMGB1 levels in the supernatant of HepG2 and PLC/PRF/5 cells under indicated treatment, *n* = 4 independent experiments. **f** Relative LDH and HMGB1 levels in the supernatant of HepG2 and PLC/PRF/5 cells 20 μM GW7647 in the presence or absence of the indicated caspase inhibitors, *n* = 4 independent experiments. **g** Representative microscopic images of HepG2 and PLC/PRF5 cells treated with GW7647 in the presence or absence of z-DEVD-FMK, MitoTEMPO, etomoxir, or siCPT1A and red arrows indicated pyroptotic cells, *n* = 4 independent experiments. Scale bars, 20 μm. **h** Relative LDH and HMGB1 levels in the supernatant of HepG2 and PLC/PRF/5 cells treated with GW7647 in the presence or absence of NAC, MitoTEMPO, etomoxir, or siCPT1A, *n* = 4 independent experiments. **i** Western blot analysis of cleaved Caspase-3 in human and mouse HCC tissues from non-FILM, FILM, and anti-PD-1-treated cohorts. **j** The quantification of mitochondrial ROS levels in human (Non-FILM = 9; FILM = 9; NR = 10; R = 6) and mouse (*n* = 6) fresh frozen HCC tissues using MitoSOX. staining. Data were shown as Mean ± SD, Each dot represents an independent biological replicate, mouse, or patient sample as indicated.  ns, not significant, *P < 0.05, **P < 0.01, ***P < 0.001. Exact *P* values are provided in Source Data files. Statistical significance was determined by two-sided Mann–Whitney U test in (**b**, **j**) and Kruskal–Wallis test in (**e**, **f**, **h**). Source data are provided as a Source Data file.

Having defined how FILM suppresses PPARA expression, we next asked how restored PPARα activity promotes GSDME-dependent pyroptosis. Given that PPARα is a ligand-activated nuclear receptor, we examined whether this effect requires ligand-dependent activation. Interestingly, although *Ppara* overexpression in HCC tissues from mouse models with FILM markedly upregulated canonical PPARα target genes (*Cpt1a*, *Fabp1*, *Acox1*), their expression remained unchanged in vitro (Supplementary Fig. 10), suggesting that ligand availability in vivo may be required for PPARα activation^32^. Consistent with this notion, neither *Ppara* overexpression nor knockout in vitro altered GSDME cleavage or terminal membrane-disruptive cell death, as evidenced by unchanged LDH/HMGB1 release and Annexin V⁺7‑AAD⁺ populations (Fig. 4a–c; Supplementary Fig. 11a–k). We next treated human and mouse HCC cells with the selective PPARα agonist GW7647, which induced cell swelling and membrane bleb formation, accompanied by increased extracellular LDH and HMGB1 release and robust GSDME cleavage, together with an increased proportion of Annexin V⁺7‑AAD⁺ cells, all of which were abolished in Gsdme‑knockout cells (Fig. 4d, e; Supplementary Fig. 11l–o). In parallel, GW7647 markedly upregulated canonical PPARα target genes (*CPT1A*, *FABP1*, *ACOX1*) and promoted nuclear translocation of PPARα (Supplementary Fig. 12a, b). Notably, a similar increase in PPARα nuclear localization was also observed in vivo in *Ppara*‑overexpressing mouse HCC tissues (Supplementary Fig. 12c), confirming bona‑fide ligand‑mediated activation.

Building on this finding, we next explored how ligand‑activated PPARα triggers pyroptosis. RNA‑seq with GSEA revealed that GW7647 treatment significant enrichment of the caspase signaling pathway (Supplementary Fig. 13a). To functionally validate the involvement of caspases, we used pharmacological inhibitors and found that blocking caspase activity with either the pan‑caspase inhibitor z‑VAD‑FMK or the caspase‑3‑specific inhibitor z‑DEVD‑FMK abolished GW7647‑induced LDH/HMGB1 release and membrane blebbing, whereas inhibition of caspase‑1 (VX‑765) or caspase‑11 (z‑LEVD‑FMK) had no effect (Fig. 4f, g), establishing caspase‑3 as the key executor. Further GSEA analysis highlighted significant enrichment of oxidative stress–related pathways (Supplementary Fig. 13b), prompting us to assess whether ROS acted upstream of caspase‑3 activation. Pharmacological inhibition revealed that blocking total ROS (NAC) or mitochondrial ROS (MitoTEMPO) prevented GW7647‑induced LDH/HMGB1 release or membrane blebbing (Fig. 4g, h). Additionally, a time-course analysis following GW7647 stimulation indicated that mitochondrial ROS increased at early time points, whereas cleaved caspase-3 and cleaved GSDME became detectable later (Supplementary Fig. 14a, b). Consistently, PPARA deletion impaired GW7647-driven ROS-caspase-3-GSDME cascade (Supplementary Fig. 14a, b). To further clarify the metabolic origin of ligand-induced ROS, we next asked whether fatty-acid oxidation (FAO), a canonical PPARα-regulated pathway, contributes to this process. Inhibition of carnitine palmitoyltransferase 1 (CPT1) with etomoxir or siRNA impaired GW7647-induced mitochondrial ROS accumulation, caspase-3 activation, GSDME cleavage, LDH/HMGB1 release and membrane blebbing (Fig. 4g, h; Supplementary Fig. 14c, d). These data indicate that ligand-activated PPARα drives FAO-linked mitochondrial redox stress, providing a metabolic basis for its pro-pyroptotic function in HCC cells.

Consistent with these findings, ROS signals and cleaved caspase-3 expression were reduced in FILM tumors from both patients and mouse models (Fig. 4i, j). Moreover, anti‑PD‑1 responders exhibited higher ROS levels and cleaved caspase‑3 expression compared to non‑responders, linking this axis to clinical response (Fig. 4i, j). Collectively, these data indicate that ligand‑activated PPARα induces pyroptosis through the ROS/caspase‑3/GSDME axis, and that suppression of this pathway contributes FILM‑associated anti‑PD‑1 resistance (Supplementary Fig. 14e).

### Ligand-activated PPARα transactivates PDK4 to promote ROS-dependent pyroptosis

**Fig. 5:**
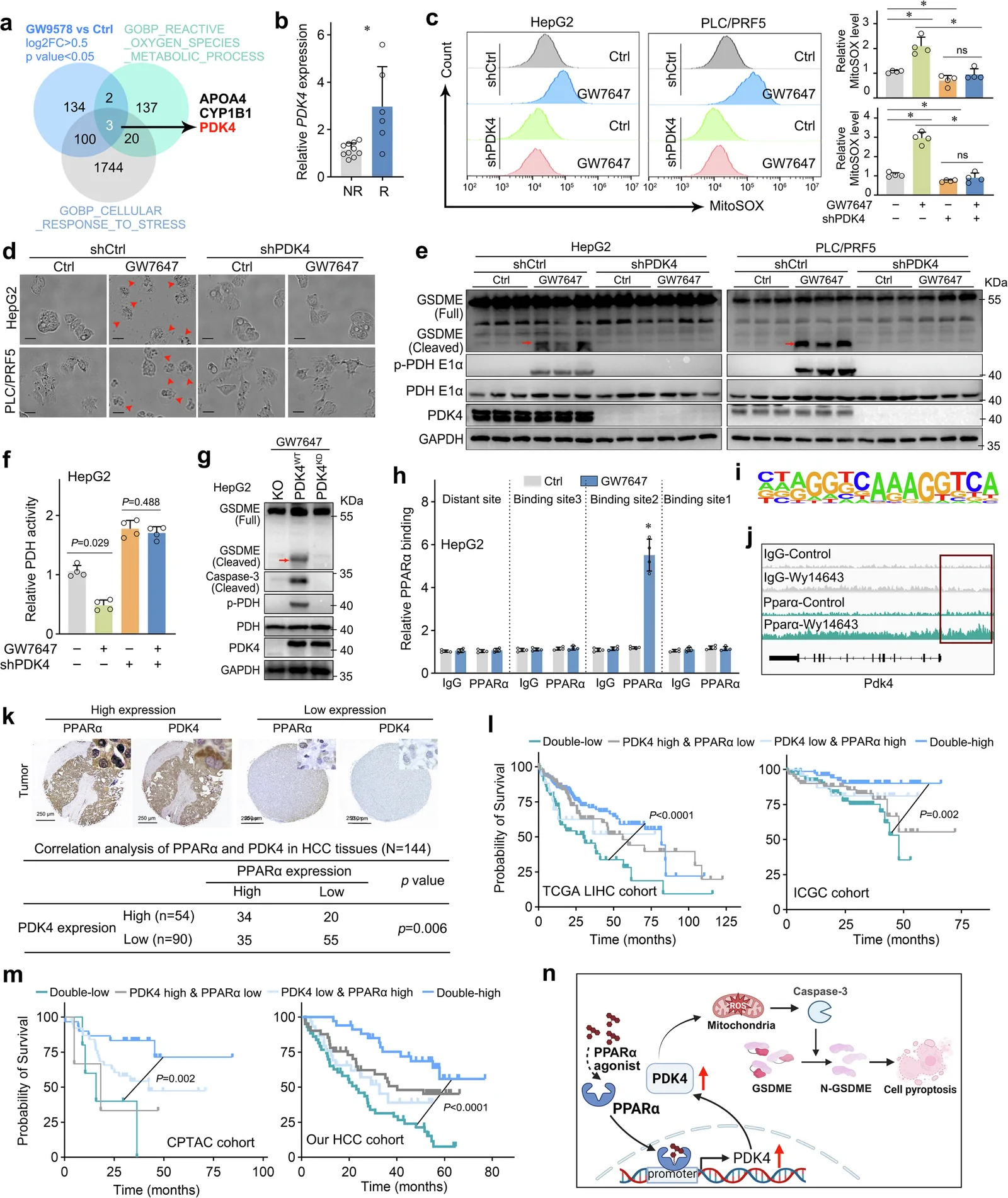
Ligand-activated PPARα transcriptionally upregulates PDK4 to boost ROS-dependent pyroptosis. **a** Overlapping genes among GW7647 upregulated genes from RNA-seq analysis of HCC cells (*n* = 3, Log2FC > 0.5 and *p* < 0.05), GOBP Reactive_Oxygen_Species_ Metabolic_Process and GOBP Cellular_Response_to_Stress. **b** *PDK4* expression in anti-PD-1 responders (*n* = 6) and non-responders (*n* = 10) HCC tissues. **c** MitoSOX fluorescen**c**e in shCtrl or shPDK4 HCC cells treated with vehicle or 20 μM GW7647, *n* = 4. **d** Representative images of shCtrl- or shPDK4-transduced cells under the indicated treatments; arrowheads indicate pyroptotic cells (*n* = 4). Scale bars, 20 μm. **e** Western blot analysis of HCC cells under indicated treatment, *n* = 3, the red asterisk marked a nonspecific band. **f** PDH activity in shCtrl- or sh*PDK4*-transduced HepG2 cells treated with vehicle or GW7647 (*n* = 4). **g** Immunoblot analysis of indicated proteins in *PDK4*-KO cells reconstituted with indicated constructs under GW7647 treatment; arrows indicate *GSDME*-N. Representative blots from *n* = 3 independent experiments. **h** ChIP assays demonstrated the direct binding of PPARα to the PDK4 promoter at the indicated binding sites, *n* = 4. **i** ChIP-seq-derived de novo motif logo of Pparα. **j** Representative PPARα ChIP-seq tracks showing PPARα occupancy at the Pdk4 locus with or without Wy14643 treatment. **k** Typical IHC staining of PPARα and PDK4 in the HCC tissue microarray and the correlation analysis. **l**, **m** Kaplan–Meier analysis of overall survival according to combined PPARα and PDK4 expression in TCGA (*n* = 364, double-high *n* = 165, double low *n* = 73), CPTAC (*n* = 76, double-high *n* = 30, double low *n* = 5), ICGC (*n* = 232, double-high *n* = 69, double low *n* = 85) and our (*n* = 147, double-high *n* = 42, double low *n* = 33) HCC cohort. **n** Proposed model. Created in BioRender. Zhong, S. (2026) https://BioRender.com/171hfdf. Data were shown as Mean ± SD, Each dot represents an independent biological replicate as indicated. ns, not significant, *P < 0.05, **P < 0.01, ***P < 0.001. Exact *P* values are provided in Source Data files.  Statistical significance was determined by two-sided Mann–Whitney U test in (**b**), Kruskal–Wallis test in (**c**, **f**, **g**), and log-rank Mantel–Cox test in (**l**, **m**). The association between PPARα and PDK4 expression (**k**) was analyzed using a two-sided Pearson χ² test. Source data are provided as a Source Data file. **l**, **m** Kaplan–Meier analysis of overall survival according to combined PPARα and PDK4 expression in TCGA, CPTAC, ICGC and an institutional HCC cohort. Sample sizes for the double-high, PPARα^high^PDK4^low^, PPARα^low^PDK4^high^ and double-low groups were 165, 17, 109 and 73 in TCGA; 30, 3, 38 and 5 in CPTAC; 69, 23, 55 and 85 in ICGC; and 42, 30, 42 and 33 in the institutional cohort, respectively.

To elucidate how PPARα activation promotes ROS accumulation, we performed transcriptomic profiling of GW7647‑treated HCC cells and intersected the upregulated genes with ROS/stress‑related gene sets, which highlighted three candidates: APOA4, CYP1B1, and PDK4 (Fig. 5a). Notably, only PDK4 was consistently downregulated in anti–PD-1 non-responders and FILM HCC tissues compared to controls (Fig. 5b; Supplementary Fig. 15a–c), and higher PDK4 expression was associated with improved patient prognosis (Supplementary Fig. 15d). Consistently, functional validation confirmed that silencing PDK4, but not APOA4 or CYP1B1, abrogated GW7647-induced mitochondrial ROS elevation (Fig. 5c; Supplementary Fig. 15e, f). Moreover, PDK4 knockdown attenuated PPARα activation-associated membrane blebbing, extracellular LDH and HMGB1 release, and the proportion of 7-AAD^+^ Annexin V^+^ tumor cells, accompanied by reduced GSDME cleavage (Fig. 5d, e; Supplementary Fig. 15g, h). To establish PDK4 as a mechanistic intermediary linking PPARα activation to mitochondrial redox stress, we first examined the PDK4-PDH axis. GW7647 treatment markedly increased phosphorylation of the PDH E1α subunit and reduced PDH enzymatic activity in control HCC cells, whereas these effects were largely impaired in PDK4-knockdown cells (Fig. 5e, f; Supplementary Fig. 16a). Furthermore, PDK4-knockout cells reconstituted with either wild-type (WT) or a kinase-dead mutant (KD) PDK4 were used to test whether the kinase activity of PDK4 is required for this pathway. Re-expression of WT PDK4 restored GW7647-induced PDH phosphorylation, mitochondrial ROS elevation, and subsequent GSDME cleavage, whereas the KD PDK4 mutant failed to do so despite comparable protein expression levels (Fig. 5f, g; Supplementary Fig. 16b–d).

We next examined how PPARα regulates PDK4 expression. *PPARA* and *PDK4* mRNA levels were positively correlated across public datasets and our HCC cohort (Supplementary Fig. 17a, b). GW7647 treatment, but not altered PPARA expression alone, markedly increased PDK4 expression, highlighting the necessity of ligand activation (Supplementary Fig. 17c–e). Bioinformatic analysis identified three putative PPARα binding sites within the PDK4 promoter (Supplementary Fig. 17f) and luciferase reporter assays showed that GW7647 treatment, but not PPARA overexpression alone, significantly enhanced PDK4 promoter activity (Supplementary Fig. 17g–i). Through serial deletion and site-directed mutagenesis, we localized the critical PPARα-responsive element to the −1188/−1205 bp region (Supplementary Fig. 17j). Furthermore, chromatin immunoprecipitation (ChIP) confirmed this requirement, demonstrating direct binding of PPARα to the PDK4 promoter only in the presence of ligand, whereas no enrichment was observed without ligand stimulation (Fig. 5h; Supplementary Fig. 17k). Consistently, analysis of public ChIP-seq datasets revealed ligand-dependent enrichment of PPARα binding near the PDK4 transcription start site (TSS), and motif analysis identified a 15-nt consensus binding sequence (Fig. 5i, j; Supplementary Fig. 17l, m).

Clinically, immunohistochemistry revealed a significant positive correlation between PPARα and PDK4 protein levels in HCC tissues (Fig. 5k). Patients with concurrent high PPARα and PDK4 expression had the best prognosis among all groups (Fig. 5l, m). Collectively, these findings demonstrate that ligand-activated PPARα directly transactivates PDK4, thereby promoting ROS-dependent GSDME pyroptosis in HCC (Fig. 5n).

### Pharmacological PPARα activation enhances anti–PD-1 therapy efficacy in HCC

**Fig. 6:**
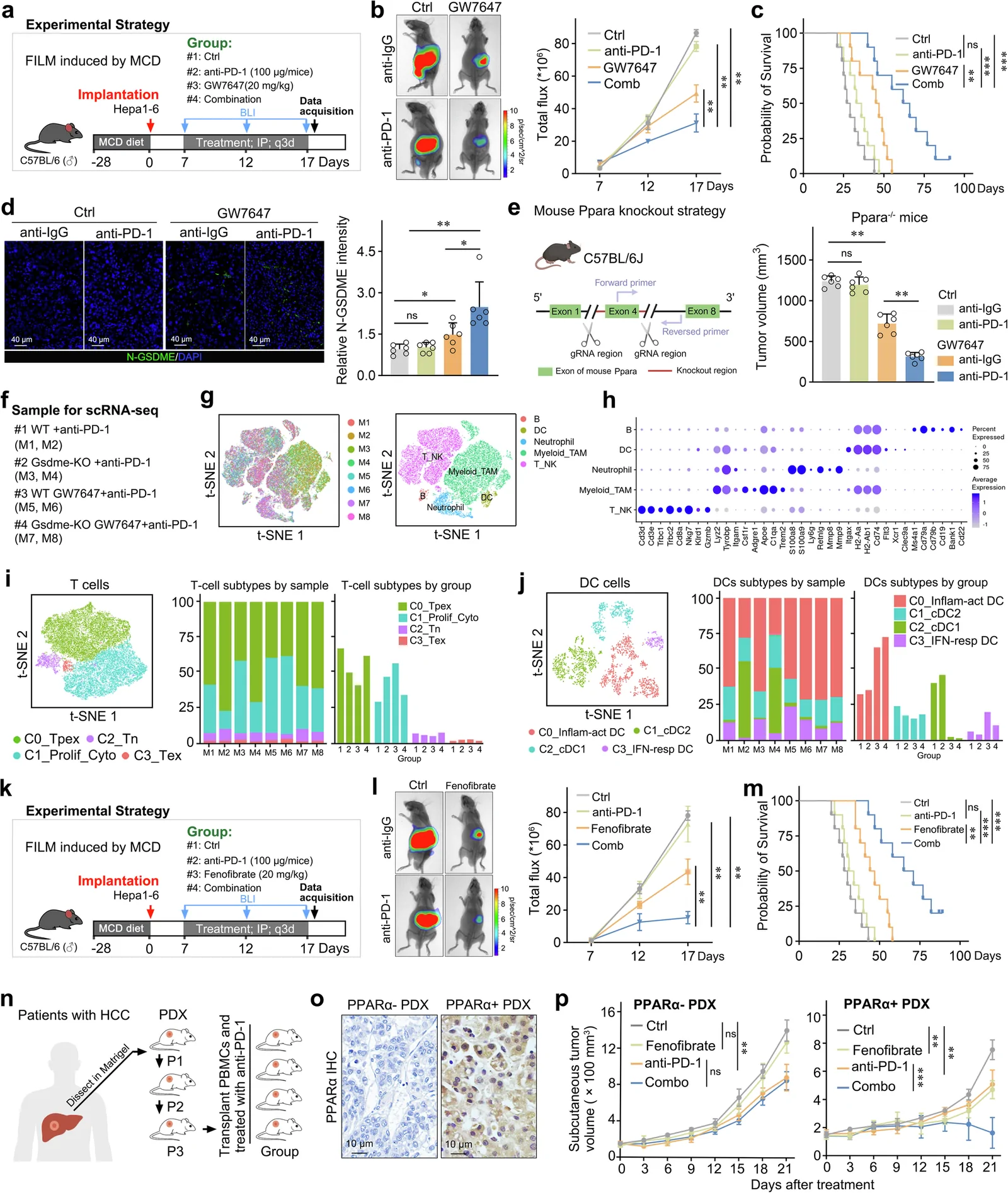
PPARα agonist fenofibrate potentiates the efficacy of PD-1 mAb against HCC in mouse models. **a** Experimental scheme for GW7647, anti-PD-1 or combination treatment in MCD-induced FILM orthotopic HCC. **b** Representative bioluminescence images and radiance (*n* = 6 mice per group). **c** Overall survival (*n* = 10 mice per group). **d** N-GSDME immunofluorescence and quantification (*n* = 6 mice per group). **e** Strategy used to generate Ppara-knockout mice, and tumor volumes in the indicated groups (*n* = 6 mice per group). **f** Strategy used to single cell RNA-seq. **g** Canonical markers used for major lineage annotation. **h** t-SNE visualization of immune cells from scRNA-seq analysis. Cells are colored by individual sample identity and by annotated major immune cell types. **i** T-cell reclustering, marker expression, functional scores and subset composition. **j** Dendritic-cell reclustering, marker expression and subset composition. **k** Experimental scheme for fenofibrate, anti-PD-1 or combination treatment in MCD-induced FILM orthotopic HCC. **l** Representative bioluminescence images and radiance (*n* = 6 mice per group). **m** Overall survival (*n* = 10 mice per group). **n** Experimental scheme for humanized HCC-PDX models generated using patient-derived tumors and human PBMCs. **o** Representative PPARα-positive and PPARα-negative PDX IHC (*n* = 4). **p** Tumor growth in PPARα-negative and PPARα-positive PDX models treated with control, fenofibrate, anti-PD-1 or combination therapy (*n* = 6 PDX-bearing mice per group). Data are mean ±s.d.; each point represents one independent mouse, patient or PDX-bearing mouse. ns, not significant, *P < 0.05, **P < 0.01, ***P < 0.001. Exact *P* values are provided in Source Data files. Statistical significance was assessed using Kruskal–Wallis tests in (**b**, **d**, **e**, **l**, and **p**), and two-sided log-rank Mantel–Cox tests in (**c** and **m**). MCD methionine- and choline-deficient, ORR objective response rate, DCR disease control rate, PDX patient-derived xenograft, PBMC peripheral blood mononuclear cell, IHC immunohistochemistry.  Source data are provided as a Source Data file.

Given that PPARα activation promotes GSDME‑mediated pyroptosis and alleviates FILM‑associated anti‑PD‑1 resistance, we next assessed whether pharmacological activation of PPARα could enhance anti-PD‑1 efficacy in PPARα-retaining HCC. In orthotopic FILM HCC mouse models, treatment with the PPARα agonist GW7647 inhibited tumor growth and significantly enhanced the efficacy of anti–PD-1 mAb (Fig. 6a–c; Supplementary Fig. 18a–c), accompanied by increased ICD-associated markers and augmented intratumoral immunity, reflected by elevated membrane CRT and cytoplasmic HMGB1, a higher proportion of 7‑AAD⁺/Annexin V⁺/CD45⁻ tumor cells, and increased frequencies of GZMB⁺ and IFNγ⁺ CD8⁺ T cells and CD86⁺ DCs (Supplementary Fig. 18d–g). Importantly, GW7647 promoted GSDME cleavage, which was further enhanced under anti‑PD‑1 treatment (Fig. 6d). Genetic deletion of Ppara in HCC cells markedly impaired the ability of GW7647 to suppress tumor growth and enhance anti-PD-1 efficacy (Supplementary Fig. 18h). Conversely, GW7647 retained its antitumor and anti-PD-1–sensitizing effects in Pparα KO mice bearing Pparα-WT HCC cells, supporting a predominant role of tumor-cell-intrinsic PPARα in this therapeutic response (Fig. 6e; Supplementary Fig. 18i, j).

To investigate whether GSDME contributes to PPARα agonist-induced immune remodeling during anti–PD-1 therapy, we established orthotopic FILM-HCC models using control or Gsdme KO HCC cells and treated mice with anti–PD-1 alone or in combination with GW7647. GW7647 markedly enhanced anti–PD-1-mediated tumor suppression and increased GZMB^+^/IFNγ^+^ CD8^+^ T cells and CD86^+^ DCs in control tumors, whereas these effects were largely attenuated in Gsdme-deficient tumors (Supplementary Fig. 19). Furthermore, we performed CD45⁺ immune-cell scRNA-seq on orthotopic FILM-HCC tumors established from control or Gsdme-deficient HCC cells (Fig. 6f; Supplementary Fig. 20a). Unsupervised clustering identified major tumor-infiltrating immune populations, including T/NK cells, TAMs, DCs, neutrophils, and B cells, which were annotated using canonical lineage markers (Fig. 6g, h). Reclustering of T cells revealed four major states, including Tpex, proliferative/cytotoxic T cells, naïve T cells, and terminally exhausted T cells (Fig. 6i; Supplementary Fig. 20b–d). PPARα agonist treatment shifted T cells toward a proliferative/cytotoxic state, whereas Gsdme deletion partially reversed this effect (Fig. 6i). We next analyzed TAM and DC compartments. PPARα agonist treatment increased the relative abundance of immune-activation-associated C0_Malt1⁺ TAMs and reduced C2_F13a1⁺ and C3_Arg1⁺ TAMs, which were enriched for tumor-promoting or TME-remodeling programs, although these TAM changes were less affected by Gsdme deletion (Supplementary Fig. 20e–i). In contrast, an IFN-responsive DC subset was expanded by PPARα agonist treatment and reduced upon Gsdme deletion (Fig. 6j; Supplementary Fig. 20j). Given that IFN-responsive/ISG⁺ DC states can activate CD8⁺ T cells and promote protective antitumor immunity, these data suggest that GSDME contributes to PPARα agonist-induced immune activation, including IFN-responsive DC expansion and subsequent T-cell remodeling during anti–PD-1 therapy.

To explore clinical translation, we assessed fenofibrate, an FDA‑approved PPARα agonist used for dyslipidemia. In an exploratory retrospective control study, 14 patients receiving fenofibrate plus anti-PD-1 were compared with 25 patients treated with anti-PD-1 (Supplementary Table 3). While objective response rate (ORR) and disease control rate (DCR) were similar between groups, the combination group exhibited a significantly greater proportion of patients achieving >20% tumor shrinkage and prolonged progression‑free survival (Supplementary Fig. 21a–c), supporting further prospective evaluation. Subsequently, across two distinct preclinical HCC mouse models—including orthotopic FILM HCC models, and DEN/CCl₄‑induced spontaneous models—fenofibrate significantly suppressed tumor growth or prolonged survival (Fig. 6k–m; Supplementary Fig. 21d–h). Notably, their combination produced the strongest antitumor effect, achieving maximal tumor inhibition or the greatest survival benefit (Fig. 6k–m; Supplementary Fig. 21d–h). Consistent with GW7647, fenofibrate in the orthotopic FILM HCC model increased ICD, reinforced intratumoral immunity, and promoted GSDME cleavage, which was further enhanced by anti‑PD‑1 treatment (Supplementary Fig. 21i–m). Moreover, in humanized immune system HCC patient‑derived xenograft (PDX) models (Fig. 6n), fenofibrate enhanced anti-PD‑1 efficacy in PPARα‑positive tumors but not in PPARα‑negative counterparts, indicating a biomarker‑dependent response (Fig. 6o, p). Collectively, these data support pharmacological PPARα activation as a strategy to enhance anti-PD-1 efficacy and overcome FILM-associated immunotherapy resistance in PPARα-positive HCC.

## Discussion

HCC immunotherapy has demonstrated limited improvements in ORR since its clinical implementation^7–9^. As HCC predominantly develops from chronically inflamed livers, emerging evidence highlights the critical role of FILM in shaping tumor immunosuppression and immunotherapy resistance^13–15,33^. Our study elucidates PPARA downregulation as a pivotal mechanism through which diverse hepatic fibroinflammatory conditions drive immunosuppressive microenvironment reprogramming in HCC. Mechanistically, PPARA downregulation reduces receptor availability and thereby dampens ligand-dependent PPARα transcriptional activity, which impairs GSDME-mediated pyroptosis via the PDK4/ROS/Caspase-3 axis. Pharmacological PPARα activation restores this pathway, inducing pyroptosis-dependent immune activation and infiltration that establishes the microenvironmental foundation for PD-1 mAb efficacy. Our integrated preclinical models and retrospective clinical analysis address the urgent need to define fenofibrate’s clinical impact on HCC immunotherapy^30^, supporting that combining an FDA-approved PPARα agonist with PD-1 mAb therapy overcomes hepatic environment-driven resistance mechanisms, providing a rationale for prospective evaluation for HCC.

The efficacy of HCC immunotherapy is strongly shaped by the immunological phenotype of the TME. Current TME-based classifications include immune-enriched (immune activation), fibrotic/immune-enriched (immune residence), fibrotic (immune suppressive) and immune-depleted (immune exclusion) types^34,35^, among which the immune-activated phenotype generally show the greatest responsiveness to PD-1 mAb therapy owing to abundant functional cytotoxic CD8^+^ T cells^36^. In this context, the response rate of PD-1 mAb monotherapy in HCC remains limited, whereas most HCCs arise in chronically inflamed and fibrotic livers^37,38^. Our prior study has established hepatic fibroinflammation as a key driver of HCC progression and therapeutic resistance^39,40^. Recent studies further reveal that hepatic fibroinflammation drives immunosuppressive reprogramming through T-cell exhaustion and infiltration of immunosuppressive cells, thereby diminishing PD-1 mAb efficacy^13,14^. Building on this foundation, our findings identify FILM-associated PPARA downregulation as a microenvironmental checkpoint suppressing pyroptosis-driven immune activation. PD-1 blockade mainly restores antitumor immune effector activity, whereas tumor-cell PPARA determines whether this immune pressure can be coupled to GSDME-mediated immunogenic cell death. In FILM tumors, reduced PPARα limits PDK4 expression, mitochondrial ROS accumulation, caspase-3 activation, and GSDME cleavage, thereby weakening the amplification of immune-mediated tumor killing. Conversely, PPARα restoration or activation does not replace immune effector mechanisms, but enables tumor cells to undergo a more immunogenic form of death under anti-PD-1 treatment, as supported by the loss of sensitization after CD8^+^ T-cell or DC depletion. We further show FILM enhances NOS2^+^ macrophage accumulation around tumors, and spatial analysis suggests that CAF-macrophage coupling may help maintain this immunosuppressive TME, a critical immunosuppressive niche in FILM^41^. The contributions of additional FILM-recruited suppressive populations, including MDSCs, Tregs, and neutrophils, remain to be clarified in future studies.

Tumoral pyroptosis releases antigens and DAMPs that promote DC maturation and tumor-specific CD8⁺ T cell responses^42,43^. GSDM family protein expression licenses tumor cells to execute pyroptosis in response to apoptotic stimuli, stress, or immune effector mechanisms within the TME^19^. Pre-existing intratumoral pyroptosis has also been identified as a predictive biomarker for immunotherapy efficacy^44,45^. Consistent with this concept, our stratified analysis of the TCGA HCC cohort revealed significant enrichment of pyroptosis signatures within the immune-enriched and fibrotic/immune-enriched subtypes which are predicted to have higher response rates to immunotherapy (Supplementary Fig. 22). Although HCC retains relatively preserved GSDME expression compared to other malignancies, its activation mechanisms appear to be suppressed. This distinction is important because GSDME abundance does not necessarily indicate GSDME activation; rather, its pyroptotic function depends on proteolytic cleavage. Thus, in FILM HCC, GSDME acts mainly as a downstream executor, whereas the FILM-sensitive regulatory nodes lie upstream at the level of PPARα–PDK4-dependent mitochondrial ROS production and caspase-3 activation. This model may also help explain why systemic fenofibrate treatment preferentially restores GSDME cleavage in FILM-conditioned tumors without implying indiscriminate GSDME activation in normal tissues, where GSDME has tissue- and context-dependent functions^46–49^. Our study reveals that FILM-mediated PPARA suppression leads to reduced intratumoral stress and impaired GSDME cleavage, a prerequisite for initiating the self-reinforcing interplay between pyroptosis and CD8⁺ T cell immunity. Specifically, impaired GSDME activation prevents the cascade where pyroptosis-derived DAMPs recruit and activate tumor-specific CD8⁺ T cells, which in turn amplify GSDME cleavage through GzmB secretion to sustain immunogenic cell death. Building upon this mechanistic understanding, we enhanced PD-1 mAb efficacy in HCC through PPARα activation, which reinstates a positive feedback loop between tumor-specific CD8⁺ T cells and GSDME-mediated pyroptosis. Collectively, our study illuminates the critical interplay among HCC immune phenotypes, pyroptotic competence, and PD-1 mAb responsiveness from a perspective anchored in fibroinflammation-driven HCC.

Mechanistically, our study identifies a mechanistic role of PPARα in HCC in linking hepatic metabolism regulation to GSDME-dependent pyroptosis. Upon ligand activation, PPARα directly occupies the functional cis-element spanning in the PDK4 promoter, enhancing its expression and thereby rewiring mitochondrial metabolism toward fatty-acid oxidation (FAO)-dependent mitochondrial ROS accumulation. This mitochondrial ROS signal subsequently activates Caspase-3 and promotes GSDME cleavage, culminating in pyroptotic tumor-cell death. Importantly, the pro-pyroptotic function of PPARα is highly context-dependent and does not contradict its canonical role in maintaining metabolic homeostasis^50,51^. In this framework, PPARA expression determines signaling capacity, whereas ligand engagement determines functional output. Accordingly, PPARA overexpression alone was insufficient to trigger pyroptosis in vitro, whereas robust activation of downstream target genes and GSDME cleavage occurred in vivo, indicating that an intact metabolic and inflammatory milieu is required to license this pathway. Mitochondrial ROS may activate caspase-3 partly through intrinsic mitochondrial death signaling, potentially involving caspase-9, although the precise apical mechanism remains to be further defined^52^. These findings extend previous studies on PPARα in HCC and may help explain why its reported effects have varied across different tumor stages and immune contexts. Our study addresses this critical gap by demonstrating that pharmacological activation of PPARα using fenofibrate significantly enhances the therapeutic efficacy of PD-1 mAb treatment. This synergistic effect is especially notable in patients with concurrent metabolic disorders, such as fatty liver disease or hyperlipidemia, as revealed by our retrospective cohort analysis, which also highlights the clinical relevance of our findings.

This study has several limitations. First, the spatiotemporal dynamics of pre-existing pyroptosis in HCC and its interplay with chronic hepatic inflammation remain undefined. This includes potential crosstalk with immunosuppressive mechanisms, which might attenuate PD-1 mAb efficacy during prolonged treatment. Second, although our data establish that PDK4 kinase activity is required for PPARα activation-induced mitochondrial ROS accumulation and GSDME cleavage, the precise apical mechanism by which PDK4-dependent mitochondrial stress activates caspase-3 remains to be further defined. Third, although our MCD- and CCl_4_-induced mouse models recapitulate key features of inflammatory HCC, they incompletely mirror the complexity of human disease. Fourth, although Hepa1-6 has been widely used in syngeneic HCC studies, we further validated our conclusions using the C57BL/6-derived Hep53.4 model, thereby minimizing potential confounding effects related to minor histocompatibility mismatch. Additionally, the retrospective fenofibrate cohort has important limitations. The sample size was small, the comparison was not randomized, and fenofibrate use may be confounded by metabolic status, liver function, concomitant therapies, and physician selection. Therefore, these clinical data should be regarded as exploratory and hypothesis-generating. They do not establish fenofibrate as an effective anti-PD-1 sensitizer in HCC. Rather, together with the mechanistic and preclinical data, they provide a rationale for prospective, biomarker-stratified studies evaluating PPARα agonists in PPARα-retaining HCC. Moreover, GW7647 and fenofibrate are not pharmacologically equivalent. GW7647 served as a selective tool compound to define the PPARα-PDK4-ROS-GSDME mechanism, whereas fenofibrate is a clinically approved fibrate with broader systemic effects. Therefore, fenofibrate’s activity in PPARα-retaining HCC supports the translational relevance of PPARα activation, while possible PPARα-independent effects should be considered.

Our study highlights a critical role of PPARα in modulating tumor immune activation and resistance to PD-1 mAb within FILM. We demonstrate that PPARα activation induces PDK4 transcription, triggering ROS/Caspase3-mediated GSDME cleavage to restore pyroptosis and antitumor immunity. Clinically, the FDA-approved PPARα agonist fenofibrate warrants prospective evaluation to reprogram the TME. Our findings provide a rationale for prospective clinical evaluation of PPARα agonist-based combination immunotherapy in HCC and potentially other fibroinflammation-associated malignancies.

## Methods

### Ethics statement

All human studies were approved by the Ethics Committee of the Second Affiliated Hospital of Nanchang University (O-Yi Yan Lun Shen [2025] No. 104), the Ethics Committee of the First Affiliated Hospital of Jinan University (KY-2024-206), and the Ethics Committee of Nanfang Hospital, Southern Medical University (NFEC-202110-K5-01). The studies were conducted in accordance with the Declaration of Helsinki and relevant institutional guidelines. Written informed consent was obtained from all participants, or the requirement for informed consent was waived by the relevant ethics committee for the retrospective use of archived, de-identified specimens and clinical data, as applicable.

All animal experiments were approved by the Bioethics Committee of Southern Medical University (IACUC-LAC-20210925-002) and were performed in accordance with the approved institutional guidelines and relevant regulations.

### HCC cell lines

The human liver cancer cell lines HepG2 (catalog no. SCSP-510; male donor) and PLC/PRF/5 (catalog no. SCSP-5095; male donor) were obtained from the Cell Bank of the Chinese Academy of Sciences (Shanghai, China). The mouse HCC cell line Hepa1-6 (catalog no. SCSP-512; C57L/J background) was obtained from the Cell Bank of the Chinese Academy of Sciences. Hep53.4 cells (catalog no. SAc0678; C57BL/6J background; male) were obtained from Warner Bio (China). HEK293T cells (catalog no. SCSP-502; female donor) used for lentiviral packaging were obtained from the Cell Bank of the Chinese Academy of Sciences. Cells were cultured in Dulbecco’s modified Eagle medium supplemented with 10% fetal bovine serum at 37 °C in a humidified incubator containing 5% CO₂. The cell lines have been authenticated by short tandem repeats (STRs) DNA profiling. All cells were tested for mycoplasma contamination before use with the Universal Mycoplasma Detection Kit (LT07-218-Lonza) and were not contaminated by mycoplasma.

### HCC tissue specimens

Cohort 1 included 147 patients with surgically resected HCC treated at Nanfang Hospital between 2006 and 2010 (129 male and 18 female; 76 aged ≤50 years and 71 aged >50 years). Archived formalin-fixed, paraffin-embedded specimens were used for histopathological analyses, and available matched tumor and adjacent non-tumor liver tissues, collected ~3 cm from the tumor margin and confirmed pathologically, were stored in liquid nitrogen for molecular analyses. Cohort 2 included 16 patients who underwent HCC resection after anti-PD-1-based therapy between January 2020 and January 2023 at the Second Affiliated Hospital of Nanchang University or the First Affiliated Hospital of Jinan University (13 male and 3 female; 6 aged ≤50 years and 10 aged >50 years). Treatment response was assessed according to mRECIST using preoperative imaging. Pathological findings were evaluated separately. Cohort 3 was a retrospective exploratory cohort of 39 patients with HCC who received anti-PD-1-based therapy with or without concomitant fenofibrate between January 2020 and January 2024 at the same institutions (29 male and 10 female; 8 aged ≤50 years and 31 aged >50 years). Eligibility required documented treatment, evaluable baseline and follow-up imaging, and sufficient follow-up for mRECIST assessment. Fenofibrate exposure was defined as documented concomitant use before the first radiological response assessment. Detailed clinicopathological characteristics are provided in Supplementary Tables 1–3. Sex was obtained from institutional medical records; gender identity was not routinely recorded. Sex was not used as a prespecified stratification factor, and sex-specific analyses were not performed because of the limited and imbalanced subgroup sizes. The studies were approved by the relevant institutional ethics committees and conducted in accordance with the Declaration of Helsinki. Written informed consent was obtained, or waived for retrospective use of archived, de-identified specimens and clinical data, as applicable. No additional compensation was provided. Residual paraffin-embedded blocks and frozen tissue aliquots are retained in the pathology archives or institutional biobanks of the respective hospitals and may be accessed through the corresponding author, subject to ethical approval, sample availability and institutional regulations.

### Histopathological assessment

Hepatic inflammation and fibrosis were evaluated using the METAVIR scoring system, a clinically validated framework widely applied in chronic liver diseases. Inflammation activity was graded as A0 no activity, A1 mild interface hepatitis and focal lobular necrosis, A2 moderate interface hepatitis and lobular necrosis, or A3 severe extensive interface hepatitis and lobular necrosis. Fibrosis was staged on a scale of F0 to F4, F0 no fibrosis, F1 portal fibrosis without septa, F2 portal fibrosis with a few septa, F3 numerous septa without cirrhosis, and F4 cirrhosis with architectural distortion and regenerative nodules. Assessments were performed by experienced hepatopathologists using hematoxylin-eosin staining to identify inflammatory changes and sirius red staining to evaluate fibrotic deposition^53,54^.

### ChIP-seq data analysis

Public PPARα ChIP-seq data with or without Wy14,643 treatment were obtained from GEO under accession number GSE154277. Normalized ChIP-seq signal tracks were used to visualize PPARα occupancy at the Pdk4 locus. Signal intensity around the Pdk4 transcription start site and PPARα-binding regions was visualized using deepTools and IGV. De novo motif analysis of PPARα-enriched regions was used to identify the consensus PPARα-binding motif.

### Bulk RNA-Seq and data analysis

Total RNA from 18 human tissue samples and 9 mouse tissue samples was extracted for bulk RNA-seq. RNA quality was assessed using an Agilent 2100 Bioanalyzer and RNase-free agarose gel electrophoresis. Eukaryotic mRNA was enriched with Oligo(dT) beads, fragmented, and reverse-transcribed into cDNA using the NEBNext Ultra RNA Library Prep Kit for Illumina. After end repair, A-tailing, adapter ligation, AMPure XP bead purification, and PCR amplification, the resulting libraries were sequenced on an Illumina NovaSeq 6000 platform by Gene Denovo Biotechnology Co. (Guangzhou, China).

Raw RNA-seq reads were processed by the sequencing service provider. Briefly, adapter sequences and low-quality reads were removed using fastp v0.20.0. Reads shorter than 100 bp after trimming, reads containing more than 10 ambiguous bases, and reads in which low-quality bases (Q ≤ 30) accounted for more than 40% of the read length were discarded. Clean reads from human samples were aligned to the GRCh38.112 reference genome using HISAT2 v2.2.1; mouse samples were aligned to the GRCm39.112 reference genome using the same pipeline. Gene-expression levels were quantified using StringTie v2.0.4 and normalized as FPKM and TPM values. Normalized expression matrices were used for expression visualization, PCA, sample clustering, immune-cell deconvolution, and gene-set enrichment analyses. Immune-cell infiltration was estimated from normalized bulk RNA-seq expression profiles. Human tissue samples were analyzed using xCell, MCPcounter, Quantiseq, and ImmunCellAI, whereas mouse tissue samples were analyzed using ImmunCellAI^55,56^.

For gene-set enrichment analyses, expression values were log2-transformed as log2(expression + 1), and genes were ranked by log2FC, calculated as the difference between the mean log2-transformed expression in FILM samples and that in non-FILM/control samples. Thus, a positive normalized enrichment score (NES) indicates enrichment in FILM, whereas a negative NES indicates enrichment in non-FILM/control samples, corresponding to relative suppression in FILM. KEGG-based GSEA was used to identify conserved signaling pathways altered between FILM and non-FILM/control HCC tissues, including PPAR signaling. GO biological-process gene sets related to pyroptosis, apoptosis, autophagy, necroptosis, and ferroptosis were used as predefined cell death-related signatures. GSEA was performed using fgsea and clusterProfiler, with a minimum gene-set size of 5 and a maximum gene-set size of 500. For fgsea analyses, 10,000 permutations were used. *P* values were adjusted using the Benjamini–Hochberg method where applicable.

### ScRNA-Seq and data analysis

Single-cell RNA-seq data from HCC patients were obtained from the GEO database under accession number GSE151530. Raw count matrices from individual samples were imported into Seurat and grouped according to the available prognosis-related clinical annotations. Cells with 200–5000 detected genes, more than 500 UMIs, and less than 20% mitochondrial gene content were retained. Potential doublets were removed using DoubletFinder on a per-sample basis before sample merging. The remaining singlet cells were merged, log-normalized, and analyzed using the standard Seurat workflow, including highly variable gene selection, scaling, PCA, UMAP/tSNE visualization, shared nearest-neighbor graph construction, and clustering. Cell-type annotation, including malignant epithelial cells and immune-cell populations, was primarily based on the reference metadata provided with GSE151530 and further validated by canonical marker genes. Malignant epithelial cells were additionally assessed using epithelial/tumor marker expression and epithelial signature scores calculated with AddModuleScore. For T-cell subpopulation analysis, annotated T cells were extracted and reclustered using the same workflow. T-cell states were inferred using T Cell State Scores calculated with the TCellSI R package, together with canonical markers and functional signatures. Pyroptosis scores were calculated using AddModuleScore based on the human GOBP_PYROPTOSIS gene set from MSigDB. Exploratory cell-level differential expression analyses were performed using Wilcoxon rank-sum tests implemented in Seurat. For malignant epithelial cells, raw counts were aggregated at the patient/sample level to generate pseudobulk profiles, followed by differential expression analysis between prognosis groups using DESeq2. KEGG GSEA was performed using preranked gene lists based on pseudobulk log2 fold changes, and functional enrichment analyses were performed using clusterProfiler.

For the mouse CD45⁺ immune-cell scRNA-seq dataset, CD45⁺ tumor-infiltrating immune cells were isolated from orthotopic FILM-HCC tumors established using control or Gsdme-deficient HCC cells under PPARα agonist and anti-PD-1-based treatment conditions. Single-cell count matrices from individual mouse tumor samples were processed using Seurat. Gene symbols were read from the 10X Genomics feature annotation files, and duplicate gene symbols were made unique before creating Seurat objects. Cells with more than 200 and fewer than 7500 detected genes and less than 15% mitochondrial gene content were retained. Potential doublets were removed using DoubletFinder on a per-sample basis. After doublet removal, singlet cells were merged, log-normalized, and analyzed using Seurat. The top 2000 highly variable genes were selected, mitochondrial percentage was regressed out during scaling, and batch effects across samples were corrected using Harmony with sample identity as the covariate. UMAP/tSNE visualization, shared nearest-neighbor graph construction, and clustering were performed using the Harmony reduction. Major immune-cell lineages, including T/NK cells, TAMs, DCs, neutrophils, and B cells, were annotated according to canonical marker genes. T/NK cells, TAM/myeloid cells, and DCs were extracted and reclustered separately using the same Seurat-Harmony workflow. Cluster marker genes were identified using FindAllMarkers with Wilcoxon rank-sum tests. Functional module scores were calculated using AddModuleScore based on curated marker gene sets or mouse MSigDB Hallmark gene sets. Cell-subset compositions were calculated from the annotated metadata and summarized at the sample and treatment-group levels. *P* values were adjusted for multiple testing where applicable. All statistical analyses and visualizations were performed in R.

### StRNA-Seq and data analysis

Spatial transcriptomic data from HCC tissue sections were obtained from published datasets^57^ and analyzed using the RCTD algorithm for cell type deconvolution. Single-cell RNA-seq objects covering major cell lineages were annotated based on available metadata, with priority given to subtype information. These references were merged and used for deconvolving cell type proportions for each spatial spot in the Visium data. Major cell populations such as CAF and macrophages were quantified by summing the relevant deconvoluted fractions at each spot. NOS2⁺ macrophage spots were defined as those with both a high macrophage fraction and positive NOS2 gene expression. The spatial distributions of CAFs, macrophages, and NOS2⁺ macrophages were visualized using ggplot2 based on spot coordinates. H&E-stained images were aligned for histological comparison. To assess pathway activities within the spatial context, the mean expression of predefined gene sets for the PPARα pathway and pyroptosis pathway was calculated for each spot and z-score normalized. Spatial feature plots were generated to visualize the distribution of pathway activities across tissue sections.

### Immunohistochemistry (IHC) staining

Human HCC surgical specimens and mouse HCC tissues were fixed in 10% neutral-buffered formalin and embedded in paraffin. Tissue sections (4 μm) were deparaffinized, rehydrated and subjected to heat-induced antigen retrieval in citrate buffer (pH 9.0) at 120 °C for 5 min. Endogenous peroxidase activity was blocked with 0.3% H₂O₂ for 15 min at room temperature, and nonspecific binding was blocked with 10% normal goat serum in PBS for 30 min at 37 °C.

Sections were incubated overnight at 4 °C with antibodies against PPARα (Abcam, ab126285; 1:200), PDK4 (Proteintech, 12949-1-AP; 1:100), HMGB1 (Abcam, ab18256; 1:200), calreticulin (Abcam, ab92516; 1:200) or Ki67 (Abcam, ab16667; 1:200), as applicable. Immunoreactivity was detected using a streptavidin–peroxidase detection kit (ZSGB-Bio) according to the manufacturer’s instructions.

Staining was independently evaluated by two pathologists blinded to the experimental groups and clinical information. Five non-overlapping microscopic fields were assessed for each section at ×200 magnification. The proportion of positive cells was scored as 0, 0%; 1, 1–25%; 2, 26–50%; 3, 51–75%; or 4, 76–100%. Staining intensity was scored as 0, absent; 1, weak; 2, moderate; or 3, strong. An immunoreactivity score ranging from 0 to 12 was calculated by multiplying the proportion and intensity scores. Scores of 0–2, 3–5, 6–8 and 9–12 were classified as negative, weakly positive, moderately positive and strongly positive, respectively.

### Oligonucleotides and plasmids

All primer, shRNA and sgRNA sequences used in this study, together with their target genes, species and applications, are provided in Supplementary Tables 4, 10 and 11. Commercial plasmids and vector backbones are listed with their suppliers and catalog or repository numbers. Newly generated promoter-reporter, expression, mutant and lentiviral constructs were generated by standard molecular cloning or site-directed mutagenesis as described below and were confirmed by Sanger sequencing. Newly generated oligonucleotide constructs, plasmids, and viral vectors are available from the corresponding author upon reasonable request.

### Inducible N-GSDME expression

The N-terminal fragment of mouse GSDME was cloned into a doxycycline-inducible lentiviral vector. Lentiviral particles were produced in 293T cells using psPAX2 and pMD2.G packaging plasmids. Hepa1-6 cells were transduced with LV-N-Gsdme or control lentivirus in the presence of 8 μg/ml polybrene and selected with hygromycin in the absence of doxycycline to allow stable cell expansion without constitutive N-GSDME toxicity. For induction, cells or tumor-bearing mice were treated with doxycycline at 1–2 μg/ml in vitro or 2 mg/ml in drinking water with sucrose in vivo. N-GSDME expression was validated by western blotting using anti-Flag or anti-GSDME antibody before downstream experiments.

### Transient transfection and luciferase reporter assay

Cells were seeded in six-well plates one day before transfection to reach ~60–70% confluence. The indicated promoter-reporter plasmids, expression constructs and pRL-TK Renilla control plasmid were transfected using Lipofectamine 3000 reagent (Invitrogen) in Opti-MEM following the supplier’s protocol. After 6 h, the medium was replaced with fresh complete medium, and cells were harvested at the indicated time points. Luciferase activity was quantified using the Dual-Luciferase Reporter Assay System (Promega). Firefly and Renilla luciferase activities were measured using a Modulus TD20/20 luminometer (Turner Biosystems), and firefly activity was normalized to Renilla activity. Results were expressed relative to the indicated control group.

### Establishment and treatment of HCC mouse models

Male C57BL/6J mice (Mus musculus; 4–8 weeks old, depending on the experimental model) were purchased from Beijing HFK Bioscience Co., Ltd. (Beijing, China). Ppara-knockout mice (Ppara^−/−^; C57BL/6JCya-Pparaem1/Cya; strain no. S-KO-16218; Mus musculus; C57BL/6JCya background) were purchased from Cyagen Biosciences (Suzhou, China). Male Ppara^−/−^ mice aged 6–8 weeks and age- and sex-matched wild-type mice on the same C57BL/6JCya background were used for the indicated experiments. Mice were housed under specific pathogen-free conditions at 20–22 °C and ~62% relative humidity, with a 12-h light/12-h dark cycle. Food and water were provided ad libitum. All animal experiments were approved by the Bioethics Committee of Southern Medical University and were performed in accordance with the approved institutional guidelines. According to the approved animal protocol, the maximal permitted tumor diameter was 20 mm. Tumor volume was calculated as length × width² / 2. Mice were euthanized at prespecified endpoints or when approved humane endpoint criteria were met, including tumor ulceration or necrosis, impaired mobility, inability to access food or water, severe ascites or abdominal distension, labored breathing, moribund appearance, or body-weight loss exceeding 20%.

### Orthotopic HCC model with a fibroinflammatory liver microenvironment

For the CCl4-induced FILM model, 4-week-old male C57BL/6J mice were gavaged with 20% CCl4 in olive oil at 100 μl per mouse for 4 weeks; controls received olive oil. For the methionine- and choline-deficient diet model, 7-week-old male C57BL/6J mice were fed an MCD diet for 4 weeks; controls received a matched control diet. After FILM establishment, mice received intrahepatic subcapsular injection of 1 × 10⁶ luciferase-expressing Hepa1-6 cells in 25 μl Matrigel-containing suspension. Tumor progression was monitored by bioluminescence imaging using an FX Pro imaging system (Bruker). Tumor and matched non-tumor liver tissues were collected at the indicated endpoints.

### DEN/CCl₄-induced HCC model

Male C57BL/6J mice received a single intraperitoneal injection of diethylnitrosamine at 25 mg/kg on postnatal day 14^58,59^. Beginning at 8 weeks of age, mice received CCl₄ at 0.2 ml/kg by intraperitoneal injection once weekly for 14 consecutive weeks. For anti-PD-1 treatment, mice received anti-PD-1 antibody at 100 μg per mouse by intraperitoneal injection every 3 days. Control mice received an equivalent amount of the corresponding isotype-control IgG. GW7647 or fenofibrate was administered at 20 mg/kg once daily by oral gavage. Mice were euthanized according to the criteria described above.

### Randomization, blinding, exclusion criteria, and sample size

For animal studies, mice were randomly assigned to treatment groups after tumor establishment based on comparable baseline tumor burden or bioluminescence intensity. Investigators performing histological scoring, immunostaining quantification, image analysis, and flow-cytometric gating were blinded to group allocation whenever feasible. Mice were excluded before randomization if tumor implantation failed or if procedure-related death occurred before treatment initiation. No animals were excluded after randomization unless predefined humane endpoint criteria were met. Sample sizes were determined based on prior experience with orthotopic HCC models and were chosen to provide sufficient power to detect biologically meaningful differences in tumor burden and immune readouts while minimizing animal use. Exact sample sizes are indicated in the corresponding figure legends.

### In vivo immune-cell depletion

For immune-cell depletion experiments, tumor-bearing mice were treated with depletion antibodies or corresponding isotype controls after tumor establishment. Anti-CD8α antibody (clone 2.43, Bio X Cell, BE0061), anti-CD4 antibody (clone GK1.5, Bio X Cell, BE0003-1), anti-NK1.1 antibody (clone PK136, Bio X Cell, BE0036), anti-Ly6G antibody (clone 1A8, Bio X Cell, BE0075-1), anti-CD20 antibody (clone MB20-11, Bio X Cell, BE0356), and anti-CD11c antibody (clone N418, Bio X Cell, BE0038) were used to deplete CD8^+^ T cells, CD4^+^ T cells, NK cells, neutrophils, B cells, and dendritic cells, respectively (Supplementary Table 7).

### Anti-CSF1R-mediated macrophage depletion

Briefly, FILM-associated orthotopic HCC models were established as described above. After tumor implantation and baseline tumor establishment, mice were randomly assigned to receive anti-mouse CSF1R antibody or isotype control. Anti-CSF1R antibody was administered intraperitoneally at 300 μg per mouse every 3 days from day 7 after tumor implantation. Control mice received the same dose of rat IgG2a isotype control following the same schedule. The anti-CSF1R antibody used was InVivoMAb anti-mouse CSF1R, clone AFS98, Bio X Cell, catalog number BE0213, its isotype is rat IgG2a, κ. At the experimental endpoint, depletion efficiency was assessed by immunofluorescence staining of F4/80⁺ tumor-associated macrophages in tumor sections (Supplementary Table 7).

### AAV-mediated macrophage-specific Nos2 knockdown

Macrophage-targeted knockdown of Nos2 was performed using an adeno-associated viral vector carrying shRNA against mouse Nos2 under the control of the F4/80 promoter. AAV8-F4/80-shNos2 and the corresponding AAV8-F4/80-shCtrl vector were generated by HanBio at a viral titer of 1 × 10¹² vg/ml. The shRNA target sequence was listed in Supplementary Table 11. For in vivo delivery, tumor-bearing mice received AAV8-F4/80-shNos2 or AAV8-F4/80-shCtrl at 1 × 10¹¹ vg per mouse via tail-vein injection on day 7 relative to tumor implantation. Mice were subsequently treated with anti-IgG, anti-PD-1, GW7647, or combined anti-PD-1 plus GW7647 therapy as indicated. Knockdown efficiency and cell-type specificity were validated by flow-cytometric analysis of NOS2 expression in tumor-infiltrating immune-cell subsets.

### Chromatin immunoprecipitation assay (ChIP)

For ChIP–qPCR, cells were fixed with 1% formaldehyde for 10 min and the reaction was quenched according to the ChIP kit protocol. Nuclei were isolated and chromatin was fragmented by sonication to obtain DNA fragments suitable for qPCR analysis. After dilution of the sheared chromatin, samples were incubated with antibodies against PPARα (Thermo Fisher, MA1-822), SP1 (Cell Signaling Technology, 9389), or control IgG, together with Protein A/G magnetic beads. Immunoprecipitated chromatin was washed, eluted and reverse-crosslinked, and DNA was purified using a PCR purification kit (QIAGEN). Enrichment at the indicated promoter regions was quantified by qPCR using primers listed in Supplementary Table 4.

### Western blotting

Total protein (30 μg per sample) was separated by SDS–PAGE and transferred onto polyvinylidene difluoride membranes (Millipore). Membranes were blocked with 5% non-fat milk for 45 min at room temperature and incubated with the indicated primary antibodies at 4 °C overnight. The following primary antibodies were used at 1:1000 dilution: anti-PPARα (Abcam, ab126285), anti-PPARγ (Abcam, ab45036), anti-PPARδ (Abcam, ab23673), anti-PDK4 (Proteintech, 12949-1-AP), anti-HMGB1 (Abcam, ab18256), anti-calreticulin (Abcam, ab92516), anti-GSDMA (Cell Signaling Technology, 49307), anti-GSDMC (Cell Signaling Technology, 61921), anti-GSDMD (Cell Signaling Technology, 39754), anti-GSDME (Cell Signaling Technology, 19453), anti-cleaved caspase-3 (Cell Signaling Technology, 9661), anti-PARP1 (Cell Signaling Technology, 9532), anti-phospho-PDH E1α (Proteintech, 29582-1-AP), anti-PDH E1α (Proteintech, 18068-1-AP), anti-SP1 (Cell Signaling Technology, 9389), anti-Flag tag (Sigma-Aldrich, F1804), anti-GAPDH (Cell Signaling Technology, 2118), anti-β-actin (Cell Signaling Technology, 4970), anti-α-tubulin (Cell Signaling Technology, 3873) and anti-lamin B1 (Proteintech, 23498-1-AP). After washing, membranes were incubated for 1 h at room temperature with HRP-conjugated anti-rabbit IgG (Cell Signaling Technology, 7074; 1:2000) or HRP-conjugated anti-mouse IgG (Cell Signaling Technology, 7076; 1:2000), as appropriate. Protein bands were detected using an enhanced chemiluminescence detection reagent (Millipore) and imaged using an enhanced chemiluminescence imaging system (Sage Creation, Beijing, China). Complete antibody information is provided in Supplementary Table 5.

### Detection of SP1 S-nitrosylation

SP1 S-nitrosylation was detected using a modified biotin-switch assay as previously described with minor modifications^60^. Briefly, freshly harvested HCC tissues or cultured HCC cells were lysed in ice-cold HEN buffer containing 250 mM HEPES-NaOH, pH 7.7, 1 mM EDTA, 0.1 mM neocuproine, 1% NP-40, and protease inhibitor cocktail. All procedures were performed under dim light and at 4 °C whenever possible to preserve labile S-nitrosothiol modifications. Free thiol groups were blocked by incubating lysates with 20 mM methyl methanethiosulfonate (MMTS) at 50 °C for 20 min with frequent vortexing. Excess MMTS was removed by protein precipitation with cold acetone. The protein pellets were resuspended in HEN buffer containing 1% SDS. S-nitrosylated cysteines were selectively reduced by adding 20 mM sodium ascorbate, followed by labeling of the newly generated free thiols with 0.4 mM biotin-HPDP for 1 h at room temperature in the dark. A parallel reaction without sodium ascorbate was included as a negative control to assess nonspecific biotin labeling. After removal of excess biotin-HPDP, biotinylated proteins were enriched using streptavidin-agarose beads overnight at 4 °C. The beads were washed extensively with HEN buffer containing 0.1% SDS, and bound proteins were eluted by boiling in SDS sample buffer containing reducing reagent. The enriched proteins were subjected to SDS-PAGE and immunoblotted with anti-SP1 antibody to detect S-nitrosylated SP1.

### RNA extraction and real-time PCR

Total RNA from cultured cells and frozen surgical HCC tissues was isolated with TRIzol reagent (Invitrogen) as instructed. cDNA was synthesized from 1 μg of total RNA with random primers with the use of the PrimeScript RT reagent Kit (TAKARA, Japan). Expression of mRNAs was assessed based on the threshold cycle (Ct), and relative expression levels were calculated as 2^−[(Ct of mRNA)–(Ct of GAPDH)]^ after normalization to GAPDH expression. Experiments were performed at least three times. All forward and reverse primer sequences used for human and mouse qRT–PCR are provided in Supplementary Table 4.

### Lentivirus production and transfection

Lentiviral plasmids containing pMD2.G, psPAX2, and target genes or corresponding control lentiviral plasmids were cotransfected into 293T cells using polyethyleneimine (PEI) according to the manufacturer’s instructions to produce lentiviral particles. Viral supernatants were collected 48 h after transfection, clarified by centrifugation, and passed through a 0.45-μm filter before use. The virus supernatants were added to HCC cells, and the transduced cells were screened with puromycin or hygromycin B 72 h after infection.

### Tumor-infiltrating immune cell isolation

Tumor-infiltrating immune cells (TIICs) were isolated from freshly harvested orthotopic HCC tissues as previously described with minor modifications. Briefly, tumor tissues were carefully dissected from the liver, and necrotic areas, visible blood clots, and surrounding non-tumor tissues were removed. The tumors were washed with cold PBS and mechanically minced into small fragments using sterile scissors. The minced tissues were digested in RPMI-1640 medium containing 2% fetal bovine serum, 1 mg/mL collagenase IV (Worthington Biochemical, LS004188), and 200 μg/mL DNase I (Worthington Biochemical, LS002139) at 37 °C for 30 min with gentle rotation.

After digestion, the cell suspension was passed through a 70 μm cell strainer to remove undigested tissue fragments and debris. Cells were centrifuged, resuspended in 30% Percoll, and carefully layered onto 70% Percoll. After density-gradient centrifugation, mononuclear cells at the interface were collected, washed twice with RPMI-1640 medium, and subjected to red blood cell lysis when necessary. The resulting cell fraction was defined as TIICs, representing a heterogeneous CD45^+^ tumor-infiltrating immune population containing lymphoid and myeloid cells. Cell number and viability were determined by trypan blue exclusion before downstream experiments.

For co-culture experiments, freshly isolated TIICs were added to HCC cells at a ratio of 10:1 and co-cultured for 48 h. After co-culture, non-adherent immune cells were removed by gentle washing, and HCC cells were collected for RNA extraction and qRT-PCR analysis. In selected experiments, the immune-cell composition of the isolated TIIC fraction was validated by flow cytometry using CD45 and lineage markers, including CD3/CD4/CD8, CD11b, F4/80, CD11c, NK1.1, and Gr-1.

### Flow cytometry assay

Single-cell suspensions were stained with LIVE/DEAD Fixable viability dye (Invitrogen, L34994) according to the manufacturer’s instructions. Fc receptors were blocked with TruStain FcX PLUS anti-mouse CD16/32 antibody (BioLegend, 156603, clone S17011E; 1 μl) for 10 min at 4 °C. Cells were subsequently incubated with the indicated fluorophore-conjugated antibodies for 30 min at 4 °C in the dark. Unless otherwise stated, antibody amounts are given per 1 × 10⁶ cells in a final staining volume of 100 μl. For immune-cell profiling, the antibody panel comprised CD45-PerCP/Cy5.5 (BioLegend, 103132, clone 30-F11; 1 μl), CD4-FITC (BioLegend, 100406, clone GK1.5; 2 μl), CD8a-PE/Cy7 (BioLegend, 162312, clone S18018E; 1 μl), CD11b-BV510 (BD Pharmingen, 562950, clone M1/70; 2 μl), CD11c-BV605 (BioLegend, 117334, clone N418; 2 μl), NK1.1-APC (BioLegend, 156506, clone S17016D; 2 μl), F4/80-BV421 (BioLegend, 123137, clone BM8; 1 μl), I-A/I-E-BV785 (BioLegend, 107645, clone M5/114.15.2; 1 μl) and Gr-1-PE (BD Pharmingen, 553128, clone RB6-8C5; 1 μl). For dendritic-cell phenotyping, cells were stained with CD45-PerCP/Cy5.5 (BioLegend, 103132, clone 30-F11; 1 μl), CD11b-BV510 (BD Pharmingen, 562950, clone M1/70; 2 μl), CD11c-BV605 (BioLegend, 117334, clone N418; 2 μl), I-A/I-E-BV785 (BioLegend, 107645, clone M5/114.15.2; 1 μl), CD80-APC (BioLegend, 104714, clone 16-10A1; 1 μl) and CD86-BV421 (BioLegend, 105123, clone PO3; 2 μl). For intracellular cytokine staining, cells were stimulated for 4 h with Cell Stimulation Cocktail plus protein transport inhibitors (Thermo Fisher Scientific). Cells were then fixed and permeabilized using an intracellular fixation/permeabilization kit (Thermo Fisher Scientific) according to the manufacturer’s instructions and stained with IFN-γ–Alexa Fluor 700 (BioLegend, 505823, clone XMG1.2; 2 μl) and granzyme B–BV421 (BioLegend, 396414, clone QA18A28; 2 μl). After staining, cells were washed and resuspended in 100 μl staining buffer. Data were acquired using an LSRFortessa flow cytometer (BD Biosciences, San Jose, CA, USA) and analyzed using FlowJo software. Antibody details, including lot numbers where available, are provided in Supplementary Table 6.

### Microscopy imaging

To examine the morphology of apoptotic and pyroptotic cells, cells were first seeded in 6-well culture dishes. Static bright-field images were captured using a Leica XSP-8CA microscope.

### LDH and HMGB1 release assay

Lactate dehydrogenase (LDH) and High Mobility Group Box 1 (HMGB1) release into the culture supernatant were measured as indicators of membrane-disruptive pyroptotic cell death. Cells were pretreated with z-DEVD-FMK (10 µM, HY-12466, MedChemExpress), Mito-TEMPO (10 µM, HY-112879, MedChemExpress) or without any of the above, for 4 h, before stimulation with GW7647 (20 µM, HY-13861, MedChemExpress). LDH and HMGB1 were measured using an LDH (BC0685, Solarbio) or HMGB1 (SEKH-0409, Solarbio) release assay kit, respectively, according to the manufacturer’s protocol. The absorbance was measured at 490 nm.

### Mitochondrial ROS measurement

MitoSOX Red dye (HY-D1055, MedChemExpress) was used to measure mitochondrial ROS according to the manufacturer’s protocol. Cells were stained with 5 µM MitoSOX Red for 30 min in the dark at 37 °C. Fluorescence intensity was measured by flow cytometry using an LSRFortessa cytometer.

### In vitro cell apoptosis assay

For the apoptosis assay, cells were seeded at 3 × 10^5^ cells/well into a six-well plate in triplicate. After 24 h, cells were subjected to indicated treatment for 24 or 48 h, and then followed by flow cytometry analysis. The cell apoptosis assay was determined according to the manual of Annexin V-PE and 7-aminoactinomycin D Apoptosis Detection Kit I (BD Biosciences). Data were analyzed by FlowJo software (FlowJo).

### CRISPR-Cas9 knockout

Gene knockout in the indicated cell lines was performed by lentiviral delivery of lentiCRISPR-v2 constructs. For gene knockouts, sgRNA (5′-CGGGGCTATTGGGACAGTCG-3′) for mouse Gsdme; (5′-GTATAACTCAATGACACCGTAGG-3′) for human GSDME; (5′-TCTGTCGGGATGTCACACAA-3′) for mouse Ppara; (5′-AGCGTGGACGAGTCTCCCAGTGG-3′) for human PPARA were cloned into lentiCRISPR-v2 (#52961, Addgene) plasmid. Lentivirus was produced by transfecting HEK293T cells. HCC cells were seeded at a density of 1 × 10^5^ cells/well in six-well plates and transfected with lentivirus. After 48 h, cells with stable GFP expression were sorted by flow cytometry, and single cells were seeded in a 96-well plate. Gene disruption was confirmed by sequencing of the targeted genomic region and by western blotting for the corresponding target protein. (Supplementary Table 11).

### PDK4 knockout and rescue experiments

PDK4-knockout HCC cells were generated as described above using the sgPDK4 sequence listed in Supplementary Table 11. For rescue experiments, PDK4-KO cells were transduced with lentiviral vectors expressing wild-type PDK4 or kinase-dead PDK4 mutant. The kinase-dead PDK4 mutant was generated by substituting Asp394 and Trp395 with alanine, and the constructs were confirmed by DNA sequencing.

### PDH activity assay

PDH enzymatic activity was measured using a PDH activity assay kit (abcam, ab109902) according to the manufacturer’s instructions. Briefly, HCC cells were treated with vehicle or GW7647 in the presence or absence of PDK4 knockdown or rescue. Cells were lysed in the assay buffer, and equal amounts of protein were loaded into assay wells. PDH activity was determined by monitoring the reduction of the assay substrate at 450 nm using a microplate reader. PDH activity was normalized to total protein concentration and presented relative to the control group.

### In vitro regulating the expression of PDK4 and PPARA

For the silence of PDK4 in HCC cells, shRNA sequences against human PDK4 (shPDK4: AACATTCTGAAGGAAATTGATAT) and mouse Pdk4 (shPdk4: CTGGACTTTGGTTCAGAAAATGC) (GeneChem, Shanghai, China) were transfected into indicated cells, respectively. The transduced cells were selected with 5 µg/ml puromycin. For PDK4 and PPARA overexpression, the custom-made lentiviral vectors with human/mouse PDK4 or PPARA cDNAs or the same for control cDNAs (GeneChem, Shanghai, China) were transfected into indicated cells which were then selected with 5 µg/ml puromycin. Knockdown and overexpression efficiencies were confirmed by qRT–PCR and western blotting before functional experiments.

### Multiplex immunohistochemistry

Multiplex immunohistochemistry was performed using the Opal 3- or 6-color Detection Kits (NEL810001KT and NEL821001KT; Akoya Biosciences) according to the manufacturer’s instructions. Formalin-fixed, paraffin-embedded tissue sections were incubated at 65 °C for 1.5 h, deparaffinized in xylene and rehydrated through a graded ethanol series. Sections were washed with double-distilled water and post-fixed in 10% neutral-buffered formalin for 10 min. Antigen retrieval was performed by microwave heating in boiling AR6 antigen-retrieval solution (pH 6.0) for 15 min. Endogenous peroxidase activity was blocked with 3% H₂O₂ for 10 min, and sections were blocked with Opal antibody diluent/blocking solution for 10 min at room temperature. Sections were incubated overnight at 4 °C with the following primary antibodies: anti-CD8 (Abcam, ab237709; 1:100), anti-CD11c (Cell Signaling Technology, 97585; 1:200), anti-α-SMA (Abcam, ab242395; 1:500) and anti-F4/80 (Abcam, ab300421; 1:200). After washing with TBST, sections were incubated with HRP-conjugated goat anti-rabbit IgG (Abcam, ab205718; 1:500) for 10 min, followed by incubation with the corresponding Opal TSA fluorophore for 10 min. After each staining cycle, antibody complexes were removed by microwave treatment in AR6 antigen-retrieval solution for 15 min. The staining procedure was repeated sequentially for each target using Opal 520, Opal 540, Opal 570 and Opal 620 fluorophores. After completion of all staining cycles, nuclei were counterstained with DAPI for 5 min at room temperature, and slides were mounted using an antifade mounting medium. Stained sections were scanned using a Vectra 3 automated quantitative pathology imaging system (PerkinElmer). Multispectral images were unmixed, segmented and quantitatively analyzed using inForm software version 2.4 (PerkinElmer).

### Statistical analysis

Data are presented as mean ± s.d. unless otherwise stated. Statistical analyses were performed using GraphPad Prism version 9.0 and R. For two-group comparisons, two-sided Mann–Whitney U tests were used unless otherwise specified. For comparisons among multiple groups, Kruskal–Wallis tests followed by appropriate multiple-comparison tests were used unless otherwise specified. Associations between categorical variables were assessed using two-sided Pearson χ² tests or Fisher’s exact tests, as appropriate. Correlations were analyzed using Pearson’s correlation analysis. Survival curves were estimated using the Kaplan–Meier method and compared using two-sided log-rank Mantel–Cox tests. The exact statistical tests used for each comparison are specified in the corresponding figure legends. Exact *P* values are provided in the corresponding figure panels or Source Data file whenever possible. *P* < 0.05 was considered statistically significant. No statistical method was used to predetermine sample size, and no data were excluded from the analyses.

### Reporting summary

Further information on research design is available in the Nature Portfolio Reporting Summary linked to this article.

## Supplementary information

Supplementary Information

Reporting Summary

Transparent Peer Review file

## Source data

Source Data

## Data Availability

Human bulk RNA-seq data generated in this study have been deposited in the Genome Sequence Archive for Human (GSA-Human) under accession number HRA019045. These data are available under restricted access because they contain human-derived sequencing data linked to patient clinical information, and access is restricted to protect patient privacy and comply with ethical requirements. The restricted dataset includes raw and processed human bulk RNA-seq data and associated de-identified clinical metadata required to interpret the analyses. Access may be requested through the GSA-Human access system for scientific research purposes. Requests should include a brief research proposal, intended use of the data, applicant affiliation and evidence of institutional or ethics approval where applicable. Access requests will be reviewed by the relevant data access committee and corresponding authors. Requests will be reviewed within 7 days, and approved data will be made available within 2 weeks. Access will be granted for the approved research purpose and for the period permitted by the data access agreement and applicable institutional requirements. Mouse bulk RNA-seq data have been deposited in the Genome Sequence Archive under accession number CRA044550. The raw and processed single-cell RNA-seq data generated in this study have been deposited in the Genome Sequence Archive under accession number CRA043152. Publicly available datasets used in this study were obtained from TCGA-LIHC via the UCSC Xena Browser (https://xenabrowser.net/), ICGC-LIRI-JP via the International Cancer Genome Consortium 25K Data Archive (https://docs.icgc-argo.org/docs/data-access/icgc-25k-data), CPTAC (https://proteomic.datacommons.cancer.gov/pdc/study/PDC000198), and the Gene Expression Omnibus, including GSE151530, GSE154277 and GSE238264. The remaining data supporting the findings of this study are available within the Article, Supplementary Information and Source Data files. Source data are provided with this paper.

## Code availability

Custom R scripts used for bulk RNA-seq GSEA, immune deconvolution, public scRNA-seq reanalysis, mouse CD45⁺ immune-cell scRNA-seq analysis, module-score calculation, functional enrichment and figure generation are available at GitHub (https://github.com/jiangych17/PPARa_GSDME_HCC_analysis) and archived on Zenodo (https://doi.org/10.5281/zenodo.20798383).
