# Supplementary material for: PPARα activation overcomes fibroinflammatory liver microenvironment-associated anti-PD-1 resistance in hepatocellular carcinoma by mediating GSDME-dependent pyroptosis
Source: Nat Commun. 2026 Aug 6;17:9474. doi: 10.1038/s41467-026-75770-7 (PMC13542117; doi:10.1038/s41467-026-75770-7)

Supplementary Information

PPARα activation overcomes fibroinflammatory liver microenvironment-associated anti-PD-1 resistance in hepatocellular carcinoma by mediating GSDME-dependent pyroptosis

Tables of contents

Supplementary Figure 1..1

Supplementary Figure 2..3

Supplementary Figure 3..5

Supplementary Figure 4..6

Supplementary Figure 5..8

Supplementary Figure 6..10

Supplementary Figure 7..12

Supplementary Figure 8..14

Supplementary Figure 9..16

Supplementary Figure 10..18

Supplementary Figure 11..19

Supplementary Figure 12..21

Supplementary Figure 13..22

Supplementary Figure 14..23

Supplementary Figure 15..24

Supplementary Figure 16..26

Supplementary Figure 17.....27
Supplementary Figure 18.....29
Supplementary Figure 19.....31
Supplementary Figure 20.....32
Supplementary Figure 21.....33
Supplementary Figure 22.....35
Supplementary Table 1.....36
Supplementary Table 2.....37
Supplementary Table 3.....38
Supplementary Table 4.....39
Supplementary Table 5.....41
Supplementary Table 6.....43
Supplementary Table 7.....44
Supplementary Table 8.....45
Supplementary Table 9.....46
Supplementary Table 10.....47
Supplementary Table 11.....48
Uncropped western blot images corresponding to Supplementary Figure 2.....49
Uncropped western blot images corresponding to Supplementary Figure 3.....50
Uncropped western blot images corresponding to Supplementary Figure 6.....51
Uncropped western blot images corresponding to Supplementary Figure 7.....52
Uncropped western blot images corresponding to Supplementary Figure 11.....53
Uncropped western blot images corresponding to Supplementary Figure 12.....54
Uncropped western blot images corresponding to Supplementary Figure 14.....55
Uncropped western blot images corresponding to Supplementary Figure 16.....56

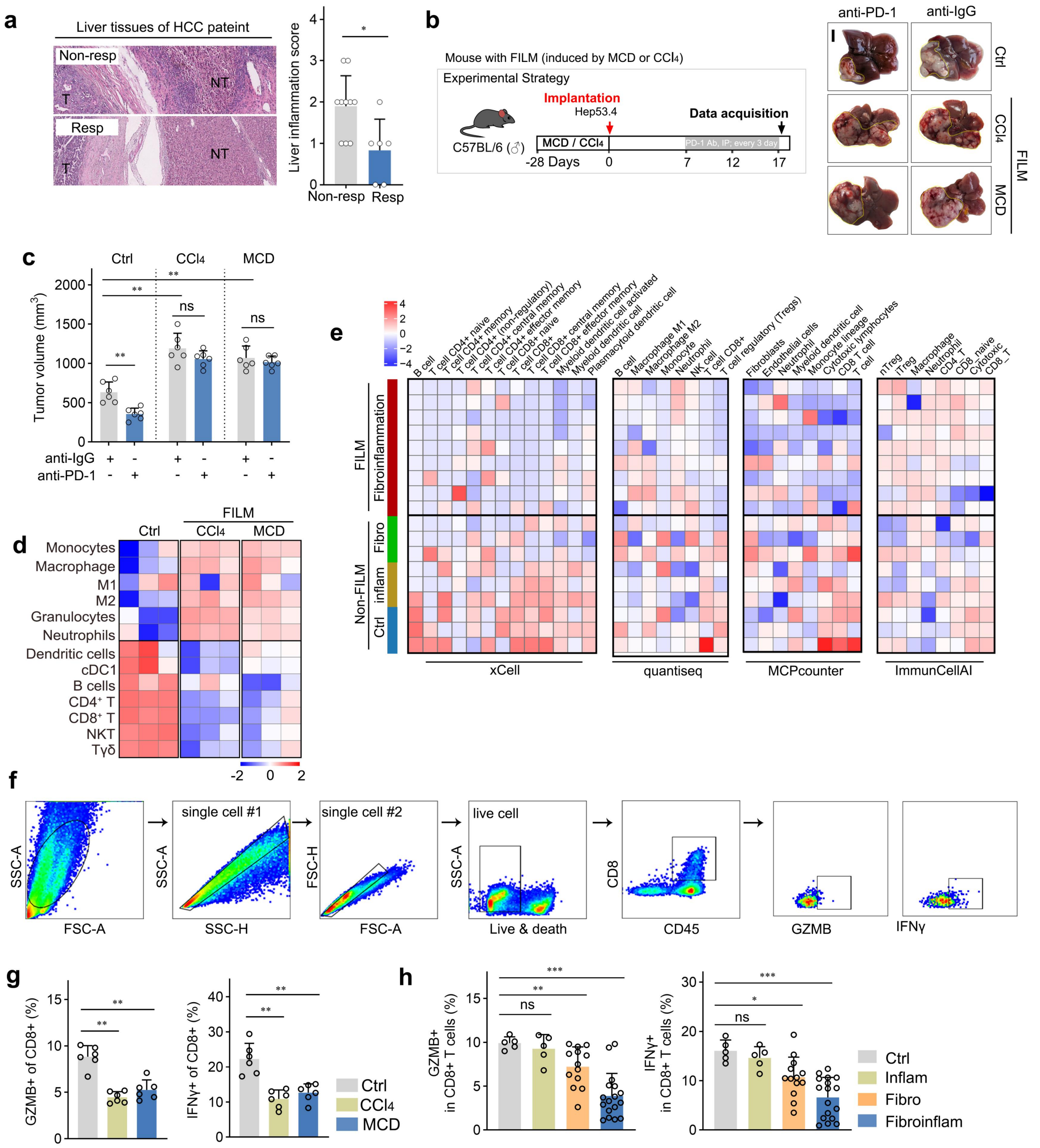

Supplementary Figure 1 Fibroinflammatory liver microenvironment drives anti-PD-1 resistance and immunosuppressive phenotype in HCC. (**a**) Representative H&E images of non-tumor liver tissues from anti-PD-1 responders (*n* = 6) and non-responders (*n* = 10), with liver inflammation scores. T, tumor; NT, non-tumor tissue. (**b**) Experimental scheme and representative gross liver images of orthotopic Hep53.4 HCC models established in control, CCl_4-induced FILM or MCD-induced FILM livers and treated with control IgG or anti-PD-1. (**c**) Tumor volumes in the indicated groups (*n* = 6 mice per group). (**d, e**) Immune-cell

deconvolution of human HCC tissues from control (*n* = 11), inflammation-dominant (*n* = 11), fibrosis-dominant (*n* = 44) and FILM (*n* = 81) groups using xCell, quanTIseq, MCP-counter and ImmuCellAI. (**f**) Sequential gating strategy for $GZMB^+$ and $IFN\gamma^+$ $CD8^+$ T cells, corresponding to Fig. 1k, l and Supplementary Fig1g. (**g**) $GZMB^+$ and $IFN\gamma^+$ $CD8^+$ T cells in control, CCl_4-induced FILM and MCD-induced FILM mouse tumors (*n* = 6 mice per group). (**h**) $GZMB^+$ and $IFN\gamma^+$ $CD8^+$ T cells in human HCC tissues from control, inflammation-dominant, fibrosis-dominant, and fibroinflammation subgroups (Ctrl n=5, Inflam n=5, Fibro n=13, Fibroinflam n=17). Data were shown as Mean ± SD, Each dot represents an independent patient sample or mouse. ns not significant, *p <0.05, **p <0.01, ***p <0.001, exact *P* value was provided in the source data. Statistical significance was determined by Mann-Whitney U test (a) and Kruskal-Wallis test (c, g, h). Source data are provided as a Source Data file.

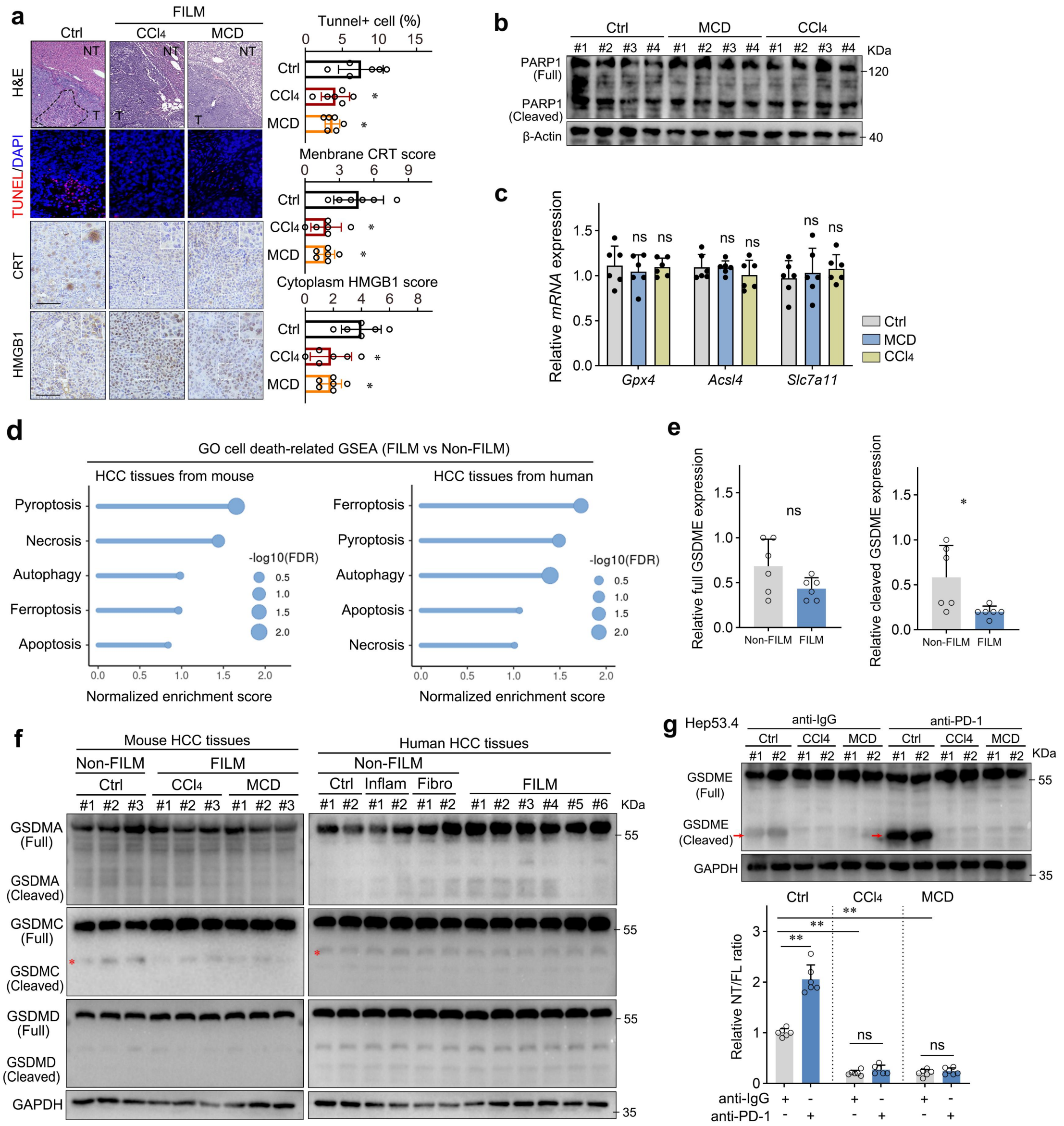

Supplementary Figure 2 FILM preferentially suppresses GSDME activation rather than broadly altering other canonical cell death pathways in HCC. (**a**) Representative H&E, TUNEL/DAPI, CRT IHC and HMGB1 IHC images in HCC tissues from control, CCl_4-induced FILM and MCD-induced FILM mice, with quantification of $TUNEL^+$ cells, membrane CRT scores and cytoplasmic HMGB1 scores (n = 6 mice per group); scale bars, 100 μm). (**b**) Immunoblot analysis of full-length and cleaved PARP1 in the indicated mouse HCC tissues (n = 4 biologically independent mice per group). (**c**) *Gpx4*, *Acsl4* and *Slc7a11* expression in the indicated mouse HCC tissues (n = 6 mice per group). (**d**) GSEA of pyroptosis, necrosis, autophagy, ferroptosis and apoptosis pathways in mouse and human FILM versus non-FILM HCC (mouse FILM n=6, mouse non-FILM n=3, human FILM n=9, human Non-FILM n=9).

(**e**) Full-length and cleaved *GSDME* in non-FILM and FILM human HCC tissues (*n* = 6). (**f**) Immunoblot analysis of full-length and cleaved *GSDMA*, *GSDMC* and *GSDMD* in mouse and human FILM and non-FILM HCC tissues; asterisks indicate nonspecific bands. (**g**) Immunoblot and quantification of *GSDME* cleavage in Hep53.4 tumors from control, CCl_4-induced FILM and MCD-induced FILM mice treated with control IgG or anti-PD-1 (*n* = 6 mice per group); arrows indicate *GSDME*-N. Data are mean ± s.d.; each point represents one biologically independent mouse or patient. ns not significant, *p <0.05, **p <0.01, ***p <0.001, exact *P* value was provided in the source data.Statistical significance was assessed using a two-sided Mann-Whitney U test in (e) and Kruskal-Wallis tests in (a), (c) and (g). Source data are provided as a Source Data file.

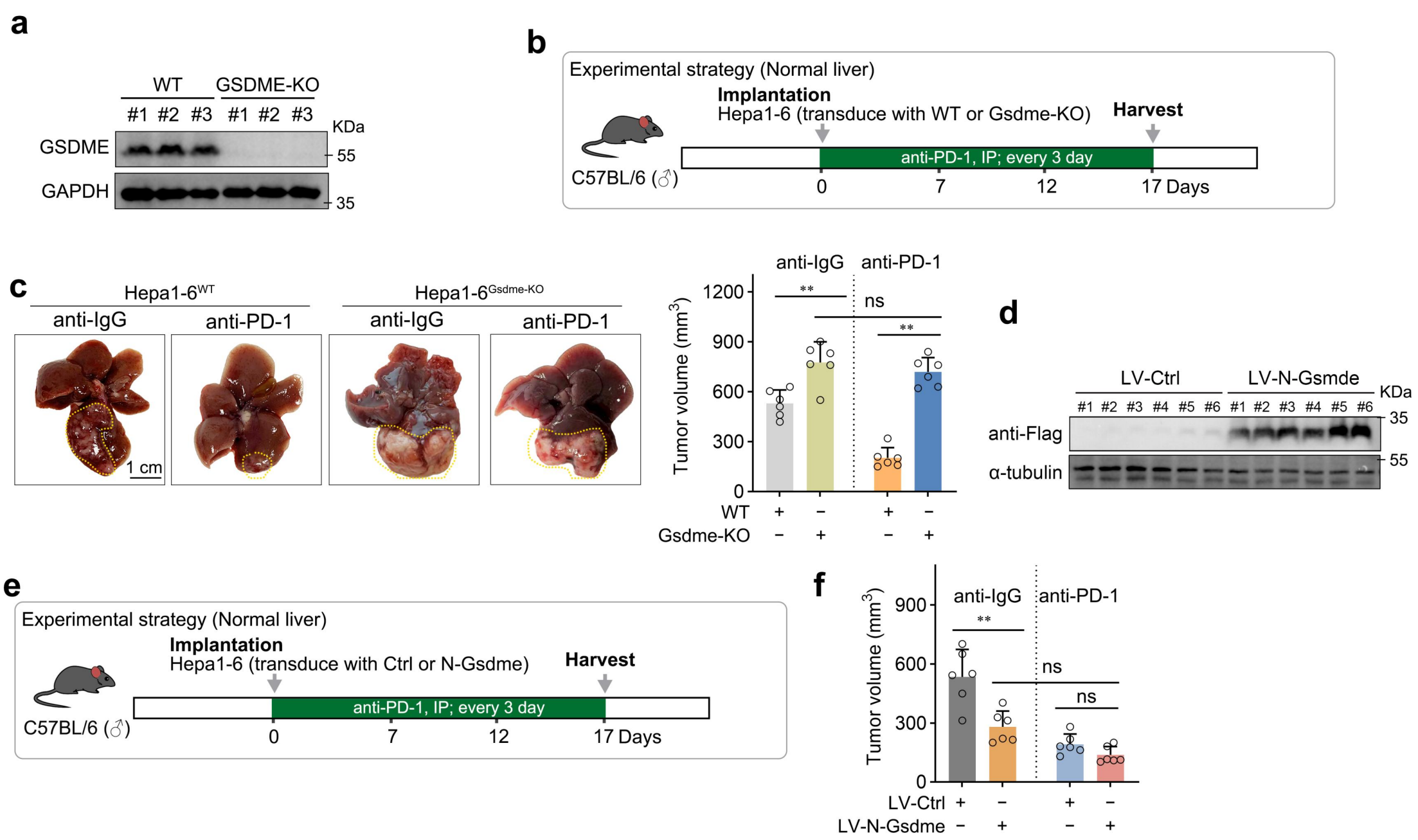

Supplementary Figure 3 GSDME is required for anti-PD-1 efficacy in non-FILM HCC models. (**a**) Immunoblot validation of *GSDME* in WT and *Gsdme*-KO Hepa1-6 cells. (**b**) Experimental scheme for orthotopic non-FILM HCC models established using WT or *Gsdme*-KO Hepa1-6 cells and treated with control IgG or anti-PD-1. (**c**) Representative gross liver images and tumor volumes in the indicated groups (n = 6). (d) Immunoblot validation of Flag-tagged N-*GSDME* in LV-Ctrl- and LV-N-*Gsdme*-transduced Hepa1-6 cells. (e) Experimental scheme for orthotopic non-FILM HCC models established using LV-Ctrl- or LV-N-*Gsdme*-transduced Hepa1-6 cells and treated with control IgG or anti-PD-1. (f) tumor volumes in the indicated groups (n = 6 mice per group). Data are mean ± s.d.; each point represents one biologically independent mouse. ns not significant, *p <0.05, **p <0.01, ***p <0.001, exact P value was provided in the source data. Statistical significance was assessed using Kruskal-Wallis tests in (c) and (f). Source data are provided as a Source Data file.

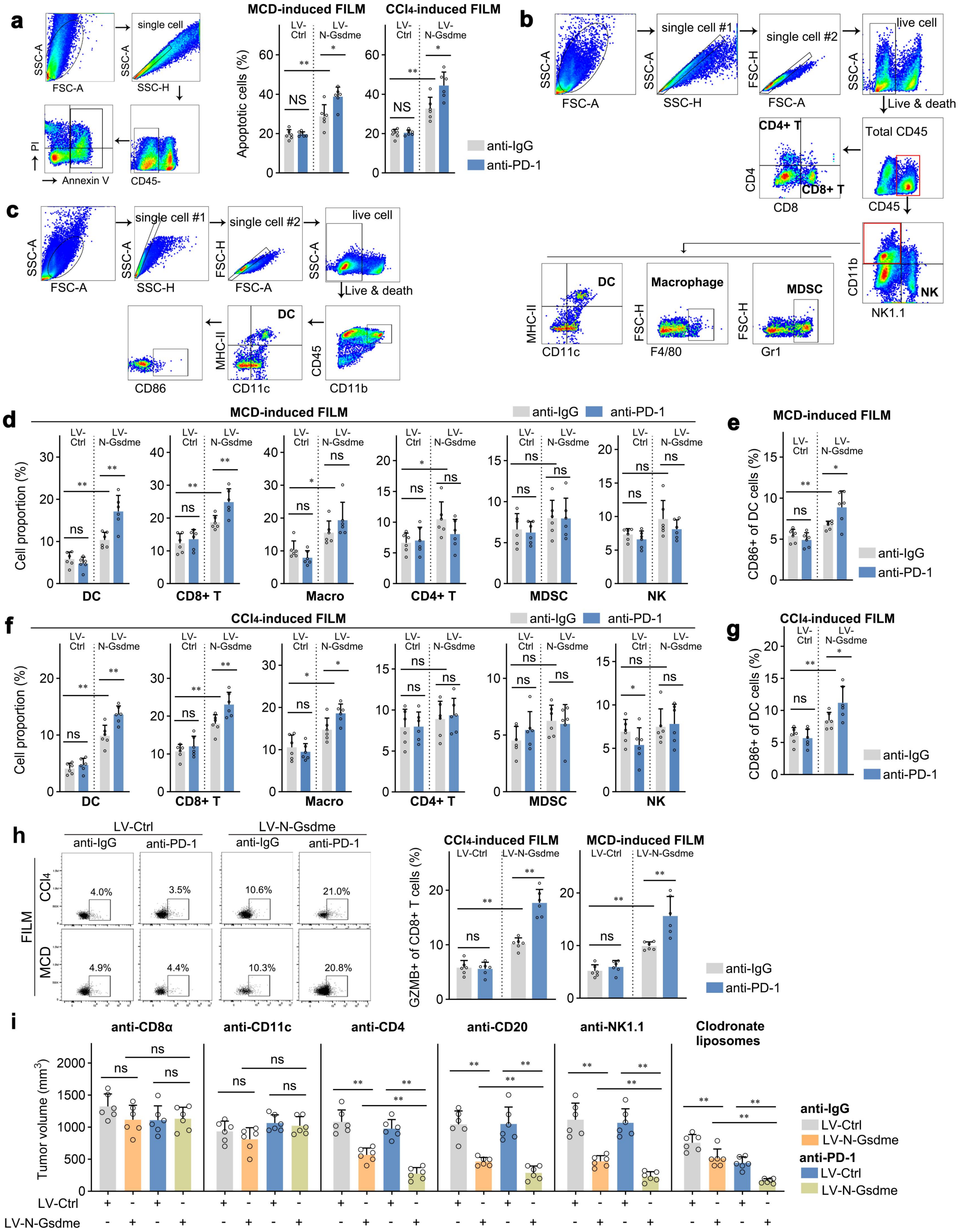

Supplementary Figure 4 N-GSDME expression restores pyroptotic tumor-cell death and enhances antitumor immune activation in FILM-associated HCC. (**a**) Gating strategy and quantification of Annexin $V^{+}PI^{+}CD45^{-}$ terminally membrane-disrupted tumor cells in CCl_4- and MCD-induced FILM tumors ($n = 6$). (**b**) Sequential gating strategy for tumor-infiltrating $CD4^{+}$ T cells, $CD8^{+}$ T cells, NK cells, dendritic cells, macrophages and MDSCs, corresponding to (d)

and (f). (**c**) Sequential gating strategy for $CD86^+$ dendritic cells, corresponding to (e) and (g). (**d-g)** Frequencies of the indicated immune-cell populations in MCD-induced (d, e) or CCl_4-induced (f, g) FILM tumors implanted with LV-Ctrl- or LV-N-*Gsdme*-transduced HCC cells and treated with control IgG or anti-PD-1 (n = 6). (**h**) Gating plots and frequencies of $GZMB^+$ $CD8^+$ T cells in the indicated groups. (**i**) Tumor volumes after depletion of the indicated immune-cell populations (n = 6). Data are mean ± s.d.; each point represents one mouse. ns not significant, *p <0.05, **p <0.01, ***p <0.001, exact P value was provided in the source data. Statistical significance was assessed using Kruskal-Wallis tests. NK, natural killer; MDSC, myeloid-derived suppressor cell. Source data are provided as a Source Data file.

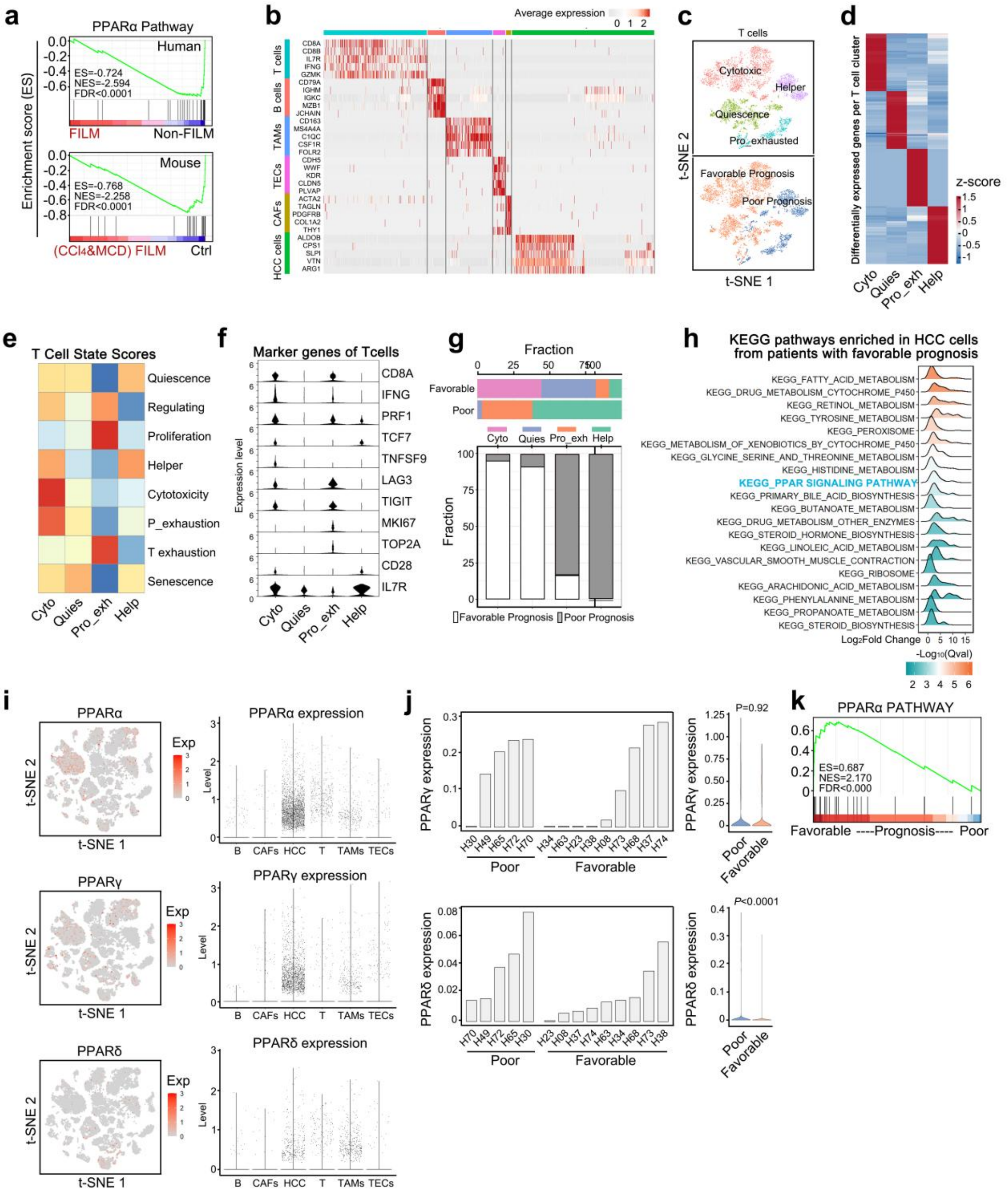

Supplementary Figure 5 Single-cell transcriptomics identifies immune landscape and PPAR signaling differences in HCC prognosis. (**a**) GSEA of PPARα signalling in FILM versus non-FILM human HCC tissues. (**b-k**) Analyses of GSE151530 single-cell RNA-sequencing data. (**b**) Marker genes used for major cell-type annotation. (**c**) t-SNE representation of T-cell subsets. (**d**) Differentially expressed genes across T-cell subsets. (**e**) T-cell state scores.

(**f**) Marker genes for T-cell subsets. (**g**) T-cell subset proportions in favourable- and poor-prognosis samples. (**h**) KEGG pathway enrichment in malignant cells from favourable- versus poor-prognosis samples. (**i**) PPARα, PPARγ and PPARδ expression across cell populations. (**j**) PPARγ and PPARδ expression in malignant cells stratified by prognosis. (**k**) GSEA of PPARα signalling in malignant cells from favourable- versus poor-prognosis samples. The numbers of patients, samples and cells included after quality control are shown in the corresponding panels. Colour scales, symbols and cell-type colours are defined in-panel. Exact *P* values are shown in the corresponding panels or Source Data file. GSEA, gene-set enrichment analysis; KEGG, Kyoto Encyclopedia of Genes and Genomes; t-SNE, t-distributed stochastic neighbour embedding. Source data are provided as a Source Data file.

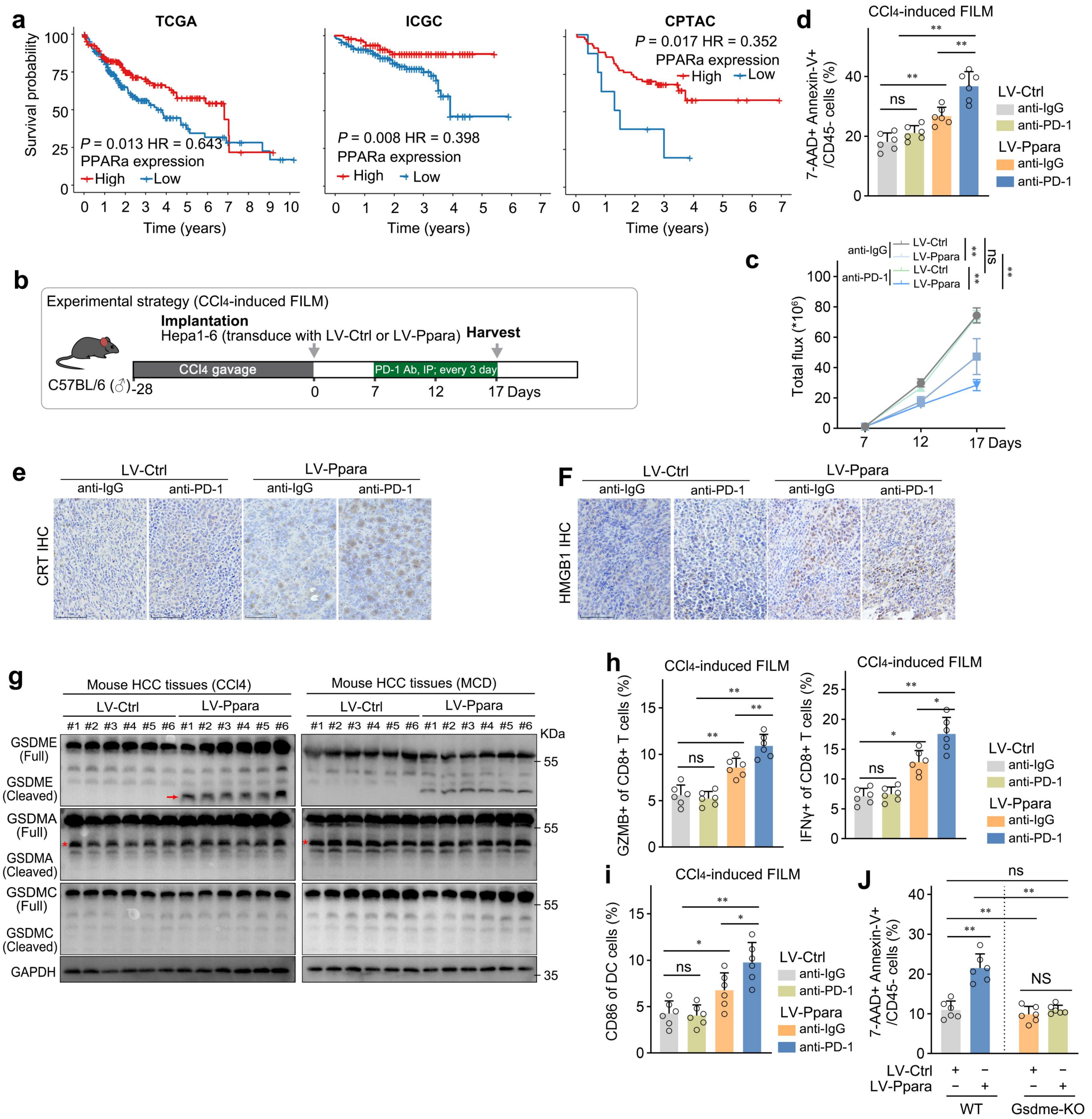

Supplementary Figure 6 PPARα activation restores anti-PD-1 sensitivity in FILM-associated HCC. (**a**) Kaplan-Meier analysis of overall survival according to PPARα expression in TCGA-LIHC (PPARα high, n =182; PPARα low, n =182), ICGC (PPARα high, n =92; PPARα low, n =140) and CPTAC (PPARα high, n =68; PPARα low, n =8) cohorts; cohort sizes are shown in-panel. (**b**) Experimental scheme for CCl_4-induced FILM orthotopic HCC models established using LV-Ctrl- or LV-*PPARA*-transduced cells and treated with control IgG or anti-PD-1. (**c**) Tumor radiance (*n* = 6 mice per group). (**d**) Annexin V^+7-$AAD^+$$CD45^-$ terminally membrane-disrupted tumor cells (*n* = 6 mice per group). (**e, f**) Representative CRT and HMGB1 IHC images (*n* = 6 mice per group; scale bars, 100 μm). (**g**) Immunoblot analysis of full-length and cleaved *GSDME*, *GSDMA* and *GSDMC* in CCl_4- and MCD-induced FILM tumors; arrows indicate *GSDME*-N and asterisks indicate nonspecific bands. (**h**) $GZMB^+$ and $IFN\gamma^+$ $CD8^+$ T

cells. (**i**) CD86⁺ dendritic cells. (**j**) Annexin V⁺7-AAD⁺CD45⁻ tumor cells in WT or *GSDME*-KO tumors with or without PPARα overexpression (h-j, *n* = 6 mice per group). Data are mean ± s.d.; each point represents one biologically independent mouse or patient. ns not significant, *p <0.05, **p <0.01, ***p <0.001, exact *P* value was provided in the source data. Statistical significance was assessed using two-sided log-rank Mantel-Cox tests in (a) and Kruskal-Wallis tests in (c), (d) and (h-j). Source data are provided as a Source Data file.

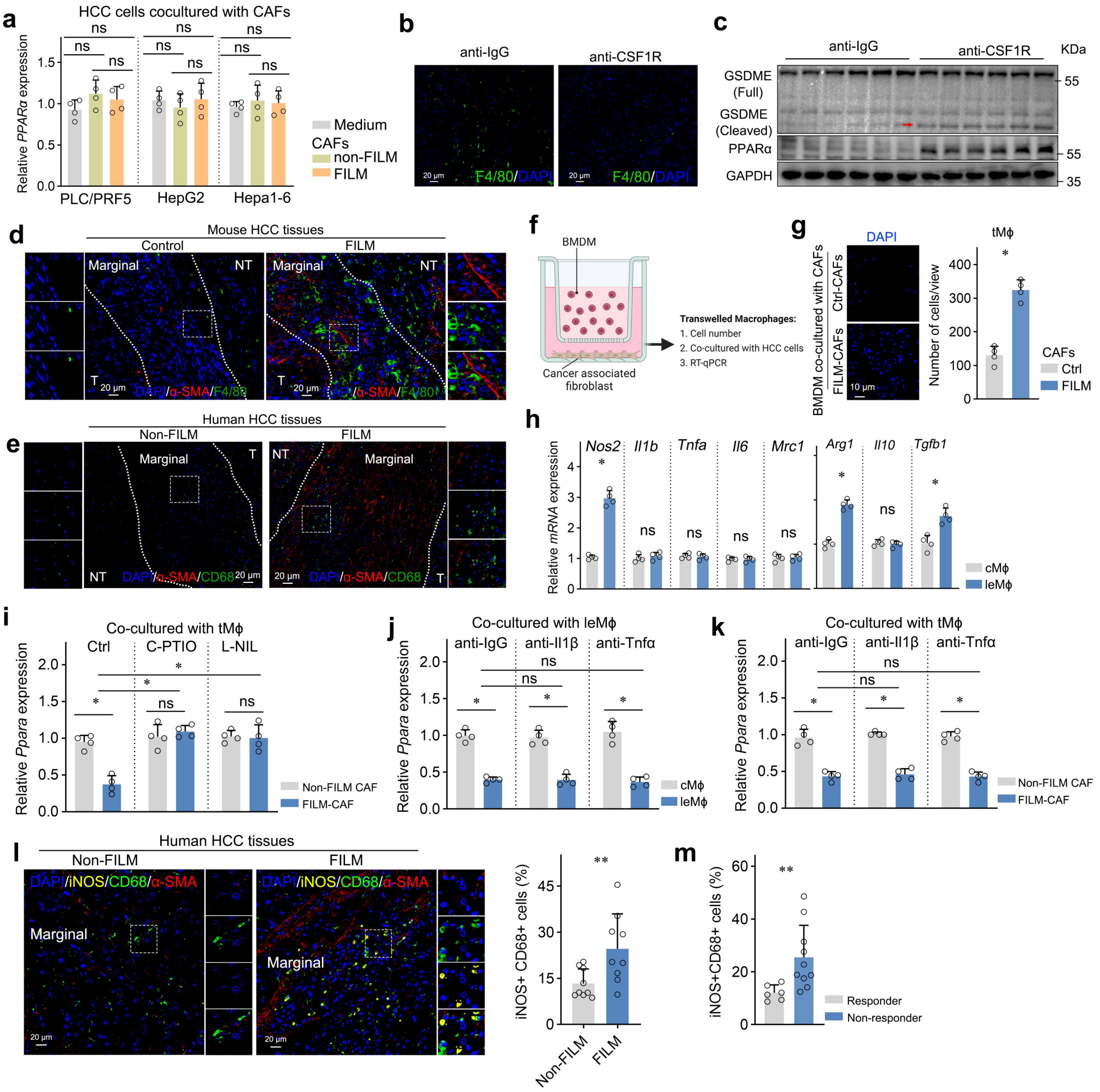

Supplementary Figure 7 FILM-derived CAFs promote NOS2⁺ macrophage accumulation and NO-dependent suppression of tumor-cell PPARα. (**a**) *PPARA* expression in PLC/PRF/5, HepG2 and Hepa1-6 cells co-cultured with CAFs from non-FILM or FILM HCC tissues (n = 4 independent experiments). (**b**) F4/80 immunofluorescence in FILM tumors treated with control IgG or anti-CSF1R (scale bars, 20 μm). (**c**) Immunoblot analysis of *GSDME* cleavage, PPARα and GAPDH in the indicated tumors (n = 6); the arrow indicates *GSDME*-N. (**d, e**) α-SMA and macrophage immunofluorescence at mouse (d) or human (e) tumor margins (n = 6 mice or patients per group; scale bars, 20 μm). (**f**) CAF-BMDM transwell co-culture scheme. (**g**) BMDM recruitment by control or FILM-derived CAFs (n = 4). (**h**) Inflammatory, M2-associated and TAM-associated gene expression in CAF-polarized macrophages (n = 4 independent experiments). (**i-k**) *Ppara* expression in HCC cells co-cultured with the indicated macrophages and treated with C-PTIO, L-NIL or neutralizing antibodies (n = 4). (**l**) iNOS,

CD68, α-SMA and DAPI multiplex immunofluorescence and quantification of $iNOS^+CD68^+$ macrophages in non-FILM and FILM human HCC. (**m**) $iNOS^+CD68^+$ macrophages in anti-PD-1 responders (*n* = 6) and non-responders (*n* = 10). Data are mean ± s.d.; each point represents one independent experiment, mouse or patient. ns not significant, *p <0.05, **p <0.01, ***p <0.001, exact *P* value was provided in the source data. Statistical significance was assessed using two-sided Mann-Whitney U tests in (b), (g), (l) and (m) and Kruskal-Wallis tests in (a) and (i-k). Source data are provided as a Source Data file.

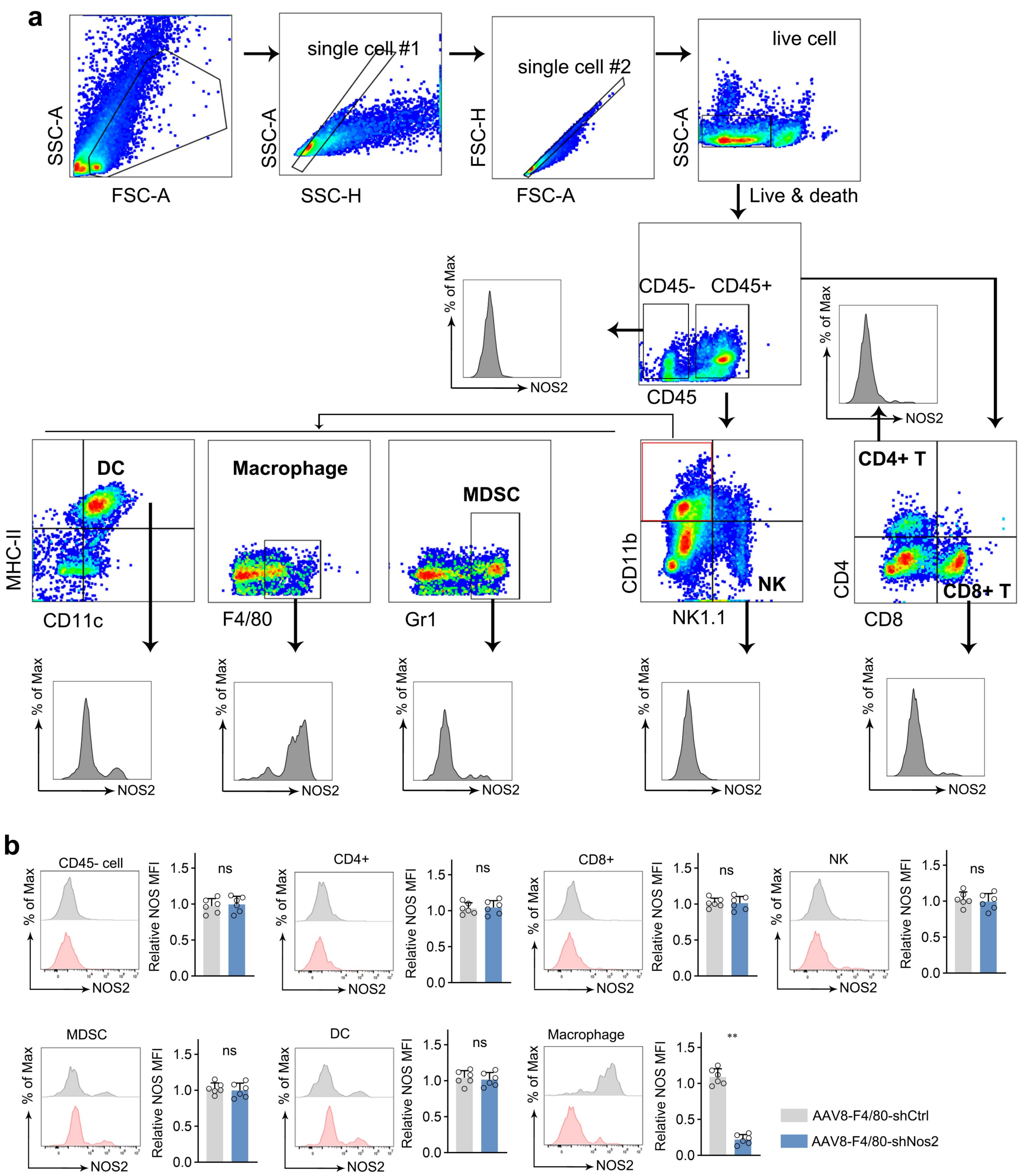

Supplementary Figure 8 AAV8-F4/80-shNos2 specifically knocks down the Nos2 expression in macrophages. (**a**) Sequential gating strategy used to quantify *NOS2* expression in tumor-infiltrating immune-cell populations from mice treated with AAV8-F4/80-shCtrl or AAV8-F4/80-sh*Nos2*, corresponding to (b). (**b**) *NOS2* expression in the indicated immune-cell populations (n = 6 mice per group). Data are mean ± s.d.; each point represents one biologically independent mouse. ns not significant, *p <0.05, **p <0.01, ***p <0.001, exact *P* value was

provided in the source data. Statistical significance was assessed using two-sided Mann-Whitney U tests. Source data are provided as a Source Data file.

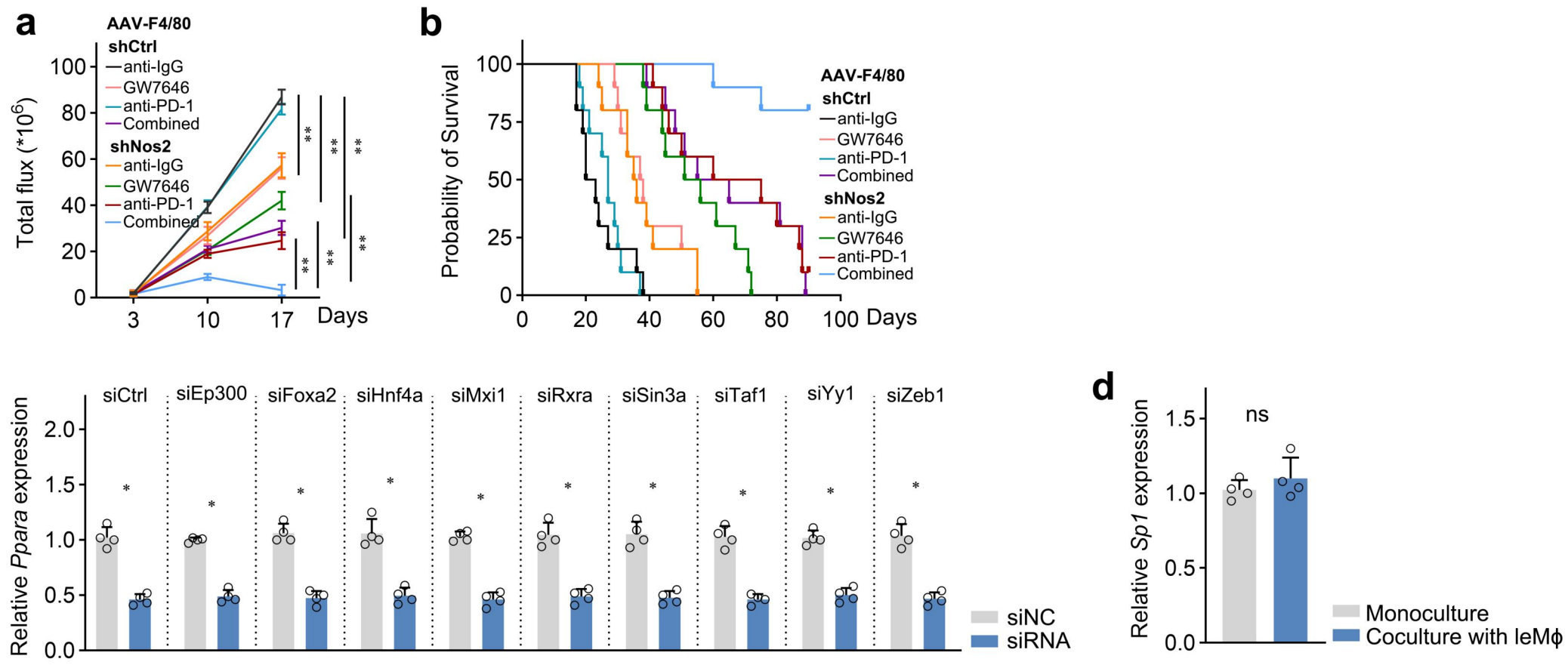

Supplementary Figure 9 NOS2 inhibition restores PPARα/GSDME activation and identifies SP1 as the key transcriptional regulator of PPARα suppression in FILM-associated HCC. (**a**) tumor growth in MCD-induced FILM-HCC models treated with AAV8-F4/80-shCtrl or AAV8-F4/80-sh*Nos2* together with control IgG, GW7647, anti-PD-1 or GW7647 plus anti-PD-1 (n = 6 mice per group). (**b**) Overall survival in the indicated groups (n = 10 mice per group). (**c**) *Ppara* expression after knockdown of candidate transcription factors (*n* = 4 independent experiments). (**d**) *Sp1* expression in HCC cells cultured alone or with leading-edge macrophages (*n* = 4 independent experiments). Data are mean ± s.d.; each point represents one independent experiment or mouse. ns not significant, *p <0.05, **p <0.01, ***p <0.001, exact *P* value was provided in the source data. Statistical significance was assessed using Kruskal-Wallis tests in (a) and (c), a two-sided Mann-Whitney U test in (d), and a two-sided log-rank Mantel-Cox test in (b). Source data are provided as a Source Data file.

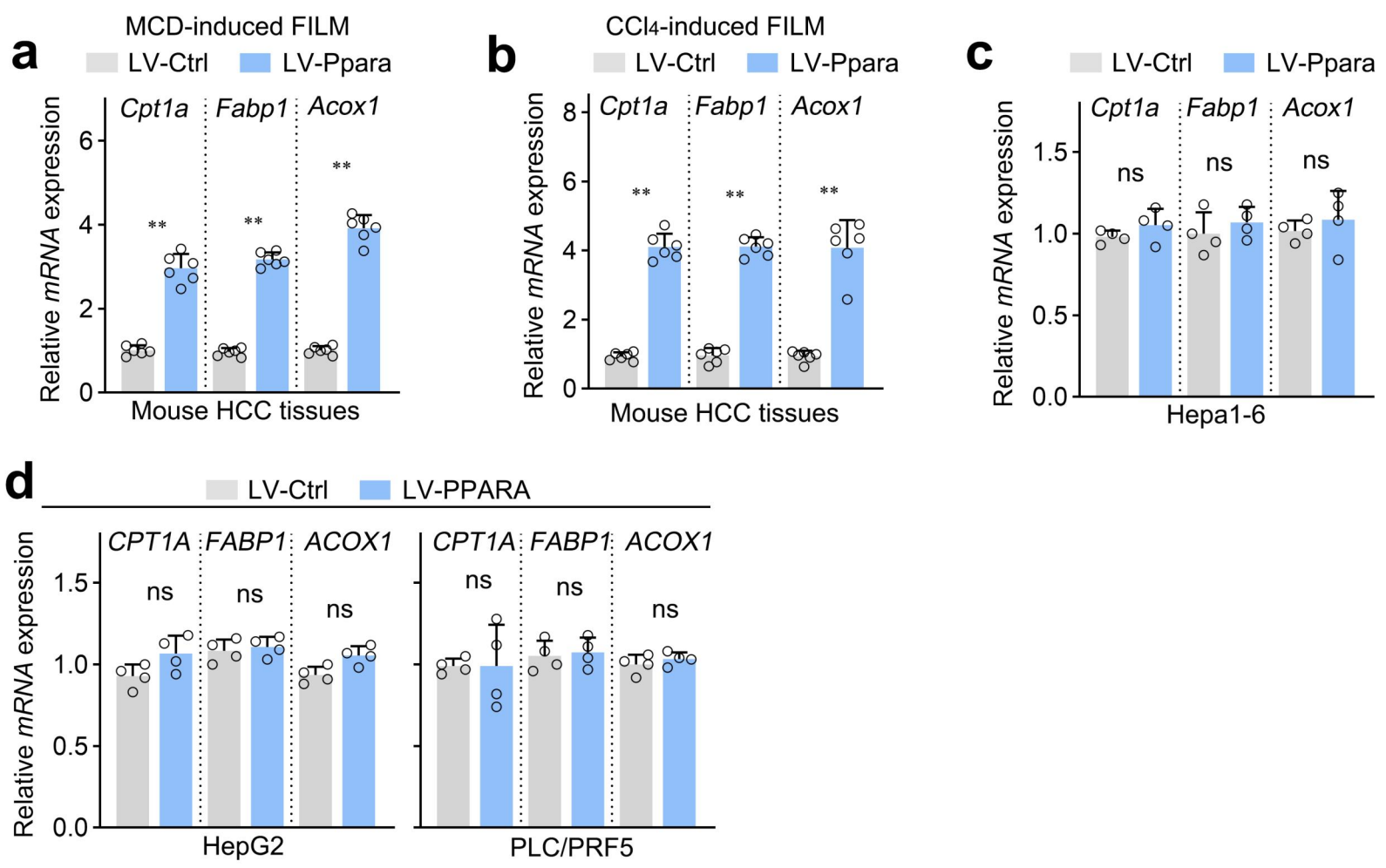

Supplementary Figure 10 Ligand-dependent PPARα activation *in vivo*. (**a, b**) *Cpt1a*, *Fabp1* and *Acox1* expression in mouse HCC tissues with *Ppara* overexpression or vector control (*n* = 6 mice per group). (**c**) *Cpt1a*, *Fabp1* and *Acox1* expression in *Ppara*-overexpressing or control Hepa1-6 cells. (**d**) *CPT1A*, *FABP1* and *ACOX1* expression in *PPARA*-overexpressing or control HepG2 and PLC/PRF/5 cells (c,d, n = 4 independent experiments). Data are mean ± s.d.; each point represents one biologically independent mouse or experiment. ns not significant, *p <0.05, **p <0.01, ***p <0.001, exact *P* value was provided in the source data. Statistical significance was assessed using Kruskal-Wallis tests. Source data are provided as a Source Data file.

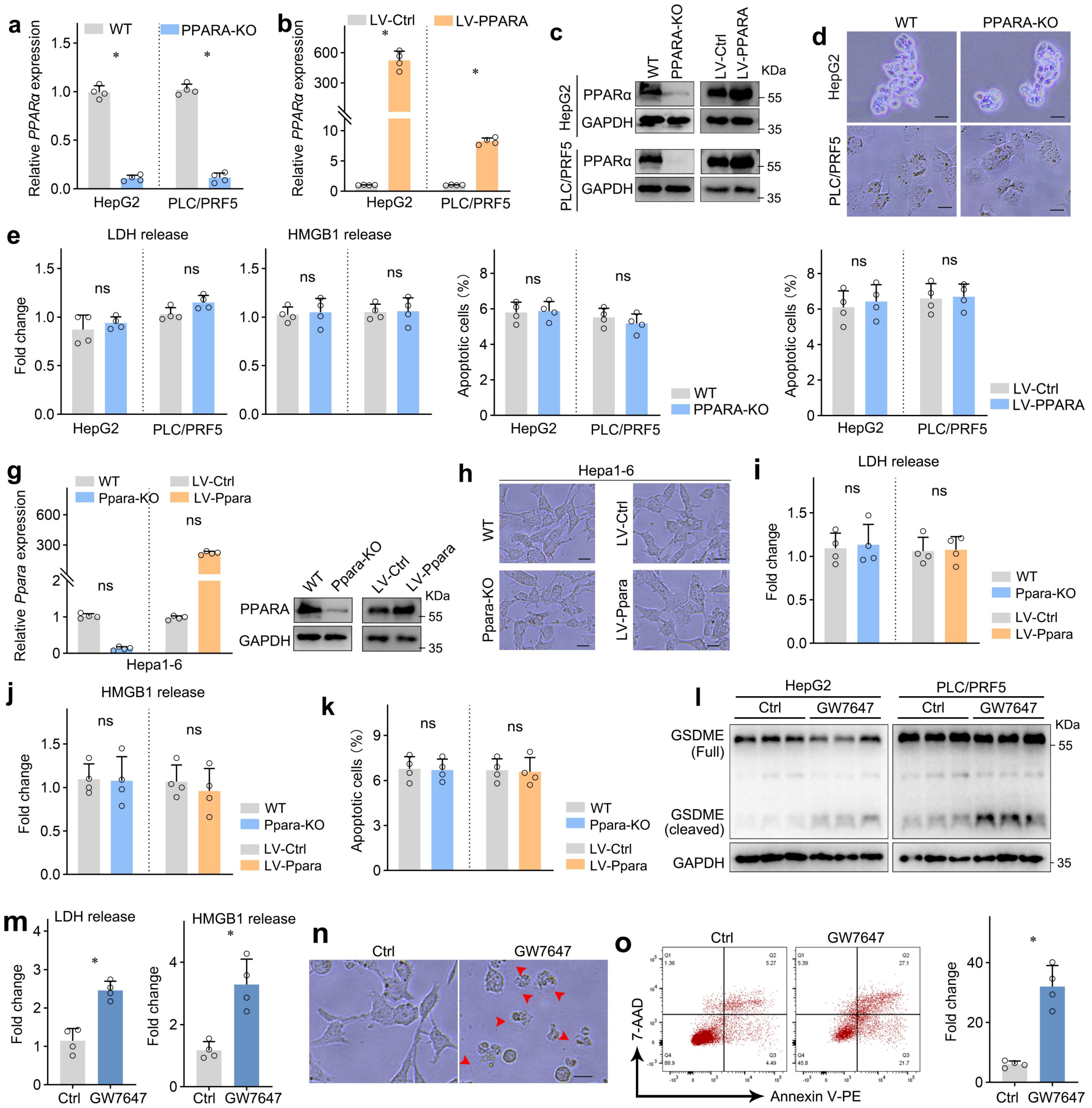

Supplementary Figure 11 The effect of PPARα activation on pyroptosis of HCC cells. (**a, b**) PPARα expression in *PPARA*-KO, LV-*PPARA* and corresponding control HepG2 and PLC/PRF/5 cells. (**c**) Immunoblot validation of PPARα under the indicated conditions. (**d**) Representative images of *PPARA*-WT and *PPARA*-KO cells (scale bars, 20 μm). (**e**) LDH and HMGB1 release from *PPARA*-WT and *PPARA*-KO cells. (**f**) Annexin V-positive cells under the indicated conditions. (**g**) *Ppara* expression and immunoblot validation in *Ppara*-KO, LV-*Ppara* and control Hepa1-6 cells. (**h**) Representative images of the indicated Hepa1-6 cells (scale bars, 20 μm). (**i, j**) LDH and HMGB1 release from the indicated Hepa1-6 cells. (**k**) Annexin V-positive Hepa1-6 cells. (**l**) Immunoblot analysis of *GSDME* cleavage in HepG2 and Huh7 cells treated with 10 μM GW7647 for 48 h. (**m**) LDH and HMGB1 release from Hepa1-6 cells treated with 10 μM GW7647 for 48 h. (**n**) Representative images of Hepa1-6 cells treated with 20 μM

GW7647 (scale bars, 20 μm). (o) Annexin V-positive Hepa1-6 cells after 10 μM GW7647 treatment for 48 h. Data are mean ± s.d.; each point represents one independent experiment. ns not significant, *p <0.05, **p <0.01, ***p <0.001, exact *P* value was provided in the source data. Statistical significance was assessed using two-sided Mann-Whitney U tests in (a), (b), (e), (f), (i-k), (m) and (o). Source data are provided as a Source Data file.

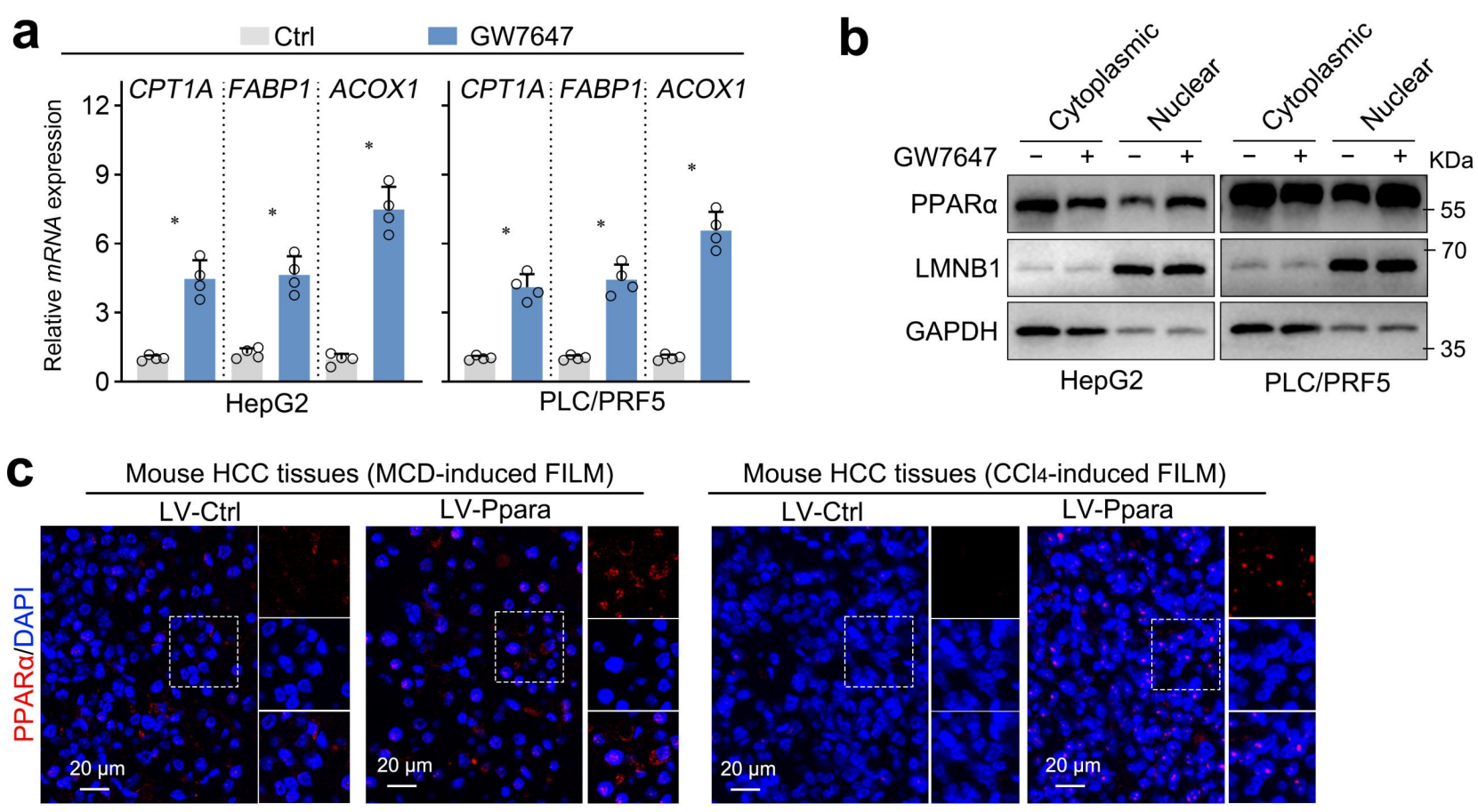

Supplementary Figure 12 Ligand-dependent PPARα activation promotes downstream gene expression. (**a**) *CPT1A*, *FABP1* and *ACOX1* expression in HepG2 and PLC/PRF/5 cells treated with 10 μM GW7647 for 48 h (*n* = 4 independent experiments). (**b**) Immunoblot analysis of cytoplasmic and nuclear PPARα in HepG2 and PLC/PRF/5 cells treated with 10 μM GW7647. (**c**) PPARα immunofluorescence in control and *Ppara*-overexpressing mouse HCC tissues (*n* = 6 mice per group; scale bars, 20 μm). Data are mean ± s.d.; each point represents one independent experiment or mouse. ns not significant, *p <0.05, **p <0.01, ***p <0.001, exact *P* value was provided in the source data. Statistical significance was assessed using two-sided Mann-Whitney U tests. Source data are provided as a Source Data file.

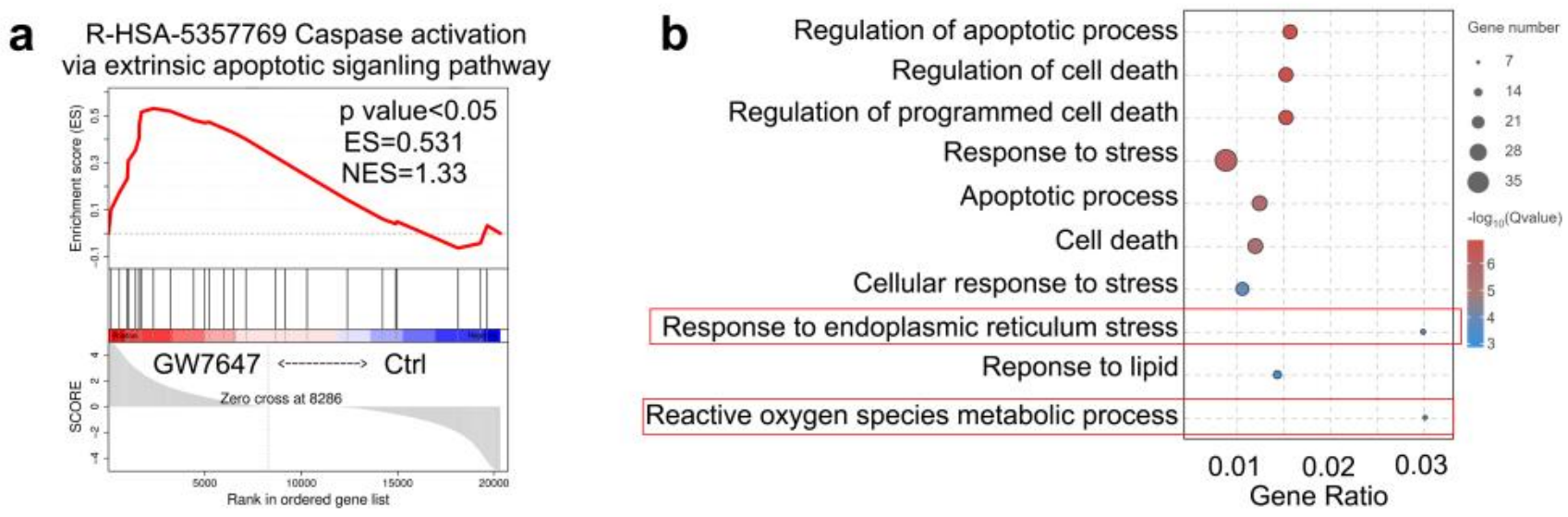

Supplementary Figure 13 Ligand-dependent PPARα activation regulates Caspase and stress-related pathways. (**a**) The association between caspase pathway activation and GW7647 treatment. (**b**) Gene ontology enrichment analysis revealed the change of cell death and stress response related pathway in HCC cells treated with GW7647.

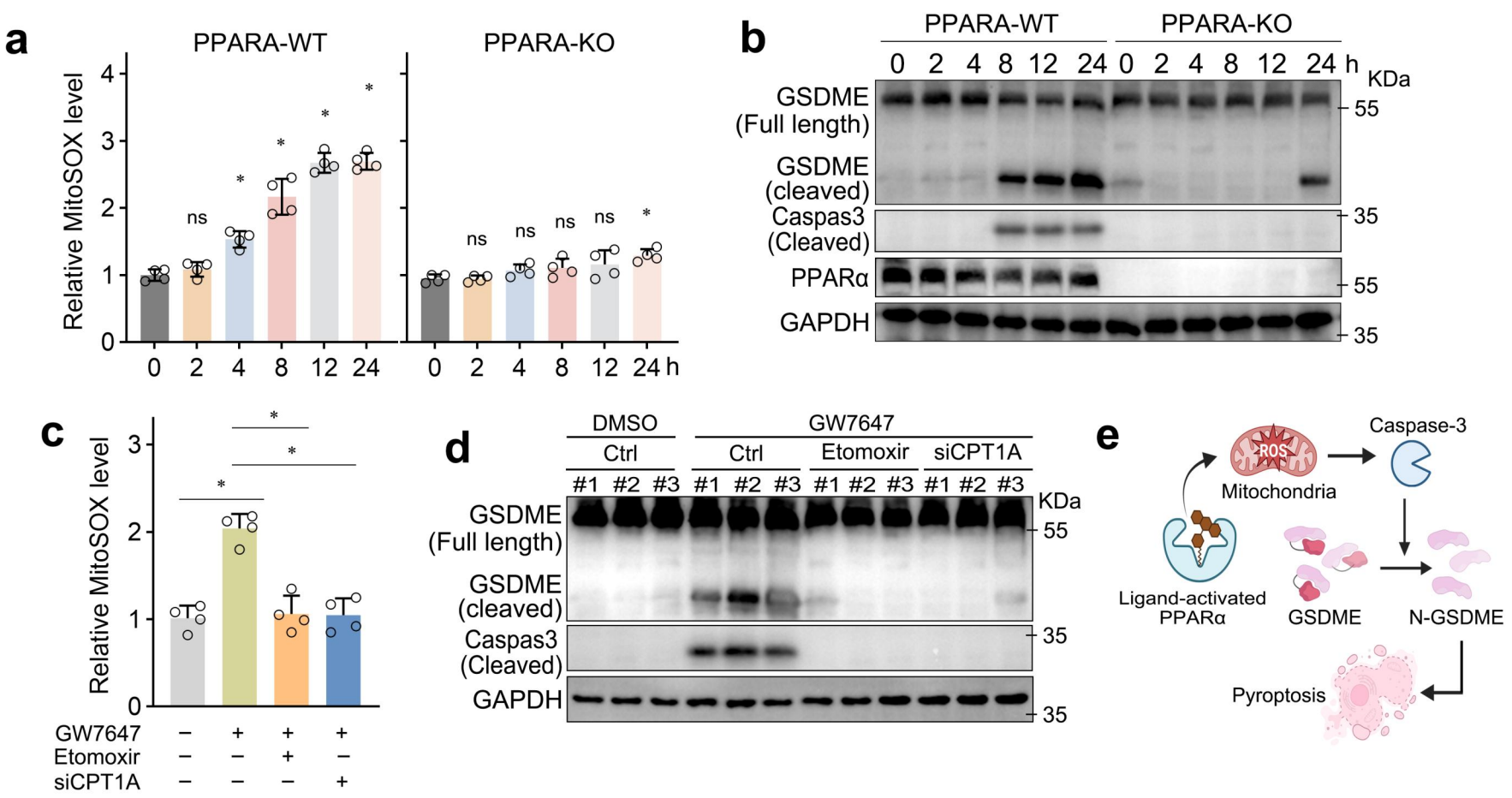

Supplementary Figure 14 PPARα activation induces FAO-dependent mitochondrial ROS accumulation upstream of caspase-3/GSDME activation. (**a**) Time-course analysis of mitochondrial ROS in *PPARA*-WT and *PPARA*-KO HCC cells after GW7647 treatment (n = 4 independent experiments). (**b**) Immunoblot analysis of full-length and cleaved *GSDME*, cleaved caspase-3, PPARα and GAPDH at the indicated time points. (**c**) Mitochondrial ROS in cells treated with GW7647 with or without etomoxir or si*CPT1A* (n = 4 independent experiments). (**d**) Immunoblot analysis of *GSDME* cleavage and caspase-3 activation under the indicated conditions. (**e**) Model of the PPARα-mitochondrial ROS-caspase-3-*GSDME* pathway, Created in BioRender. Zhong, S. (2026) https://BioRender.com/171hfdf. Data are mean ± s.d.; each point represents one independent experiment. ns not significant, *p <0.05, **p <0.01, ***p <0.001, exact P value was provided in the source data. Statistical significance was assessed using Kruskal-Wallis tests. Source data are provided as a Source Data file.

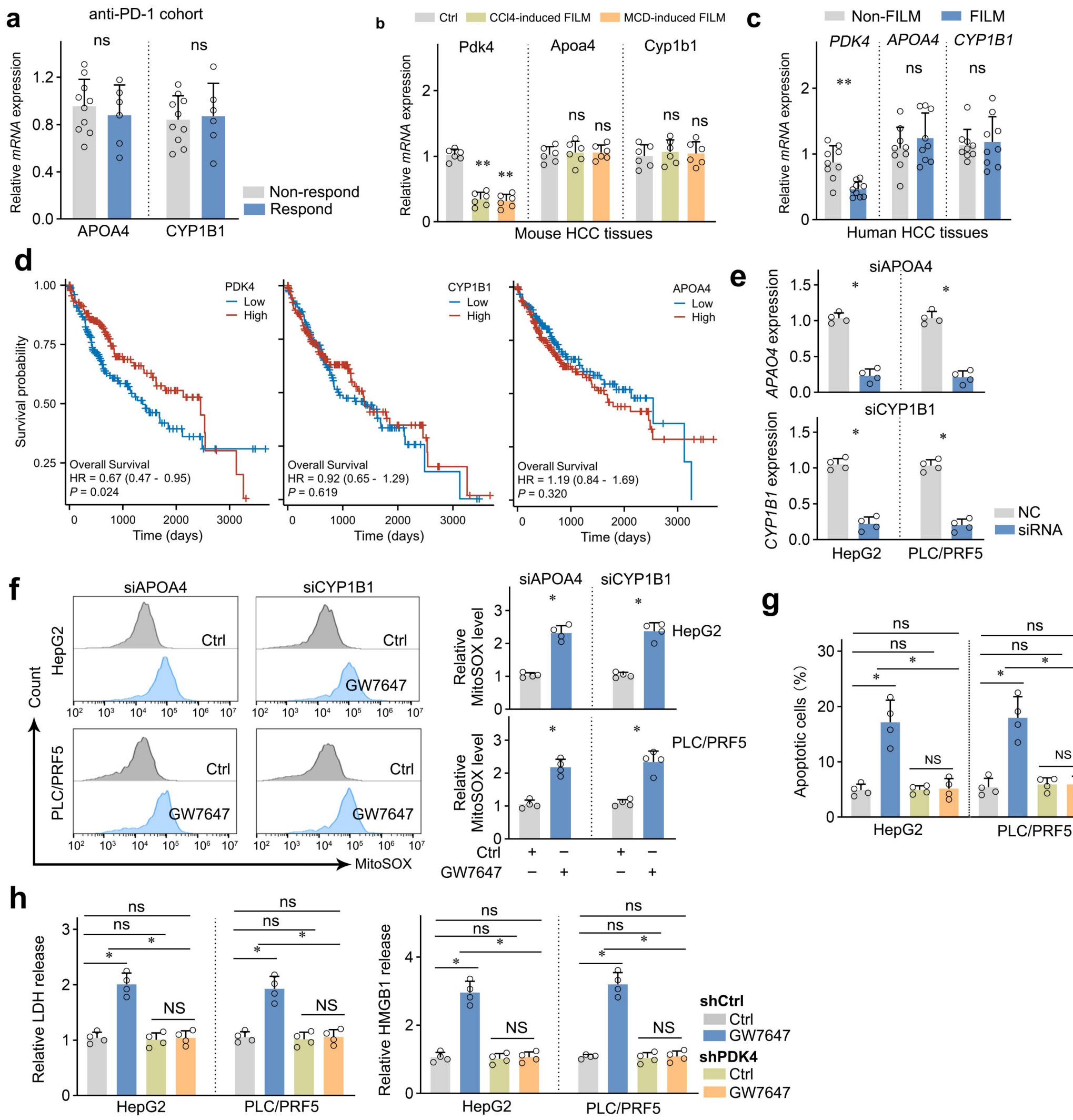

Supplementary Figure 15 PDK4 regulates PPARα activation-mediated pyroptosis. (**a**) *APOA4* and *CYP1B1* expression in HCC tissues from anti-PD-1 responders (*n* = 6) and non-responders (*n* = 10). (**b**) *Pdk4*, *Apoa4* and *Cyp1b* expression in non-FILM and FILM mouse HCC tissues (n = 6 mice per group). (**c**) *PDK4*, *APOA4* and *CYP1B1* expression in non-FILM and FILM human HCC tissues (*n* = 9 patients per group). (**d**) Overall survival according to *PDK4*, *APOA4* or *CYP1B1* expression in TCGA-LIHC (n=364, high = 182, low=182); sample sizes are shown in-panel. (**e**) *APOA4* and *CYP1B1* expression after the indicated siRNA treatments (*n* = 4). (**f**) Mitochondrial ROS in HepG2 and PLC/PRF/5 cells under the indicated conditions (*n* = 4). (**g**) Annexin V-positive cells after sh*PDK4* or shCtrl transduction and 10 μM

GW7647 treatment for 48 h (n = 4). (**h**) LDH and HMGB1 release under the conditions in (g) (e-h, n = 4 independent experiments). Data are mean ± s.d.; each point represents one independent experiment, mouse or patient. ns not significant, *p <0.05, **p <0.01, ***p <0.001, exact P value was provided in the source data. Statistical significance was assessed using two-sided Mann-Whitney U tests in (a), (c), (e) and (f), Kruskal-Wallis tests in (b), (g) and (h), and two-sided log-rank Mantel-Cox tests in (d). Source data are provided as a Source Data file.

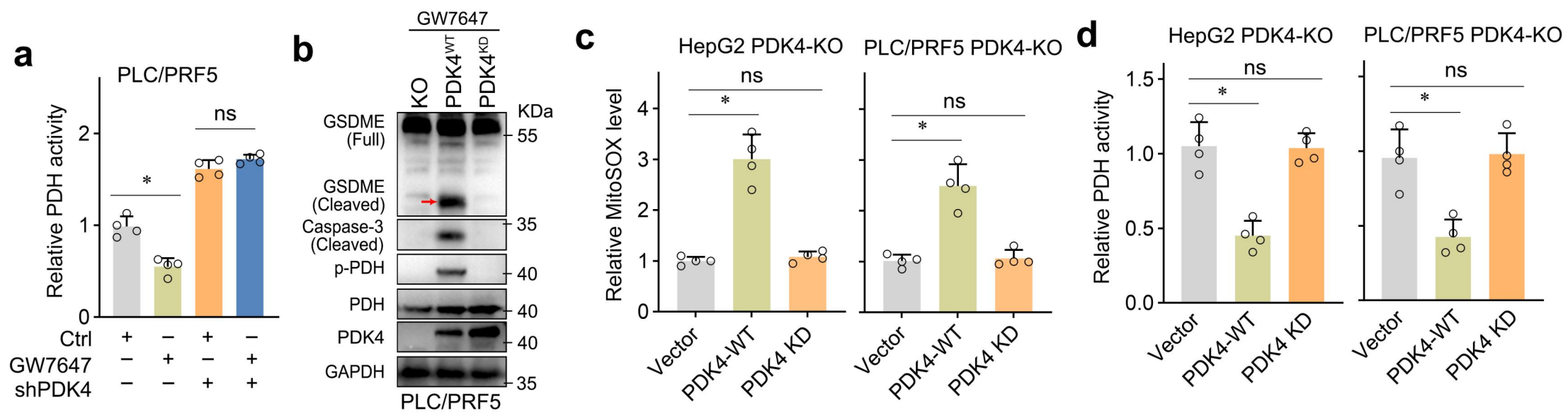

Supplementary Figure 16 PDK4 kinase activity is required for PPARα activation-induced mitochondrial ROS accumulation and GSDME-mediated pyroptosis. (**a**) PDH activity in shCtrl- or sh*PDK4*-transduced PLC/PRF/5 cells treated with vehicle or GW7647. (**b**) Immunoblot analysis of *GSDME* cleavage, caspase-3 activation, phosphorylated and total PDH, and *PDK4* in *PDK4*-KO cells reconstituted with vector, WT *PDK4* or kinase-dead *PDK4* under GW7647 treatment; arrows indicate *GSDME*-N. (**c**) Mitochondrial ROS in the cells described in (b). (**d**) PDH activity in the cells described in (b). For quantitative assays, n = 4 independent experiments. Data are mean ± s.d.; each point represents one independent experiment. ns not significant, *p <0.05, **p <0.01, ***p <0.001, exact *P* value was provided in the source data. Statistical significance was assessed using Kruskal-Wallis tests. Source data are provided as a Source Data file.

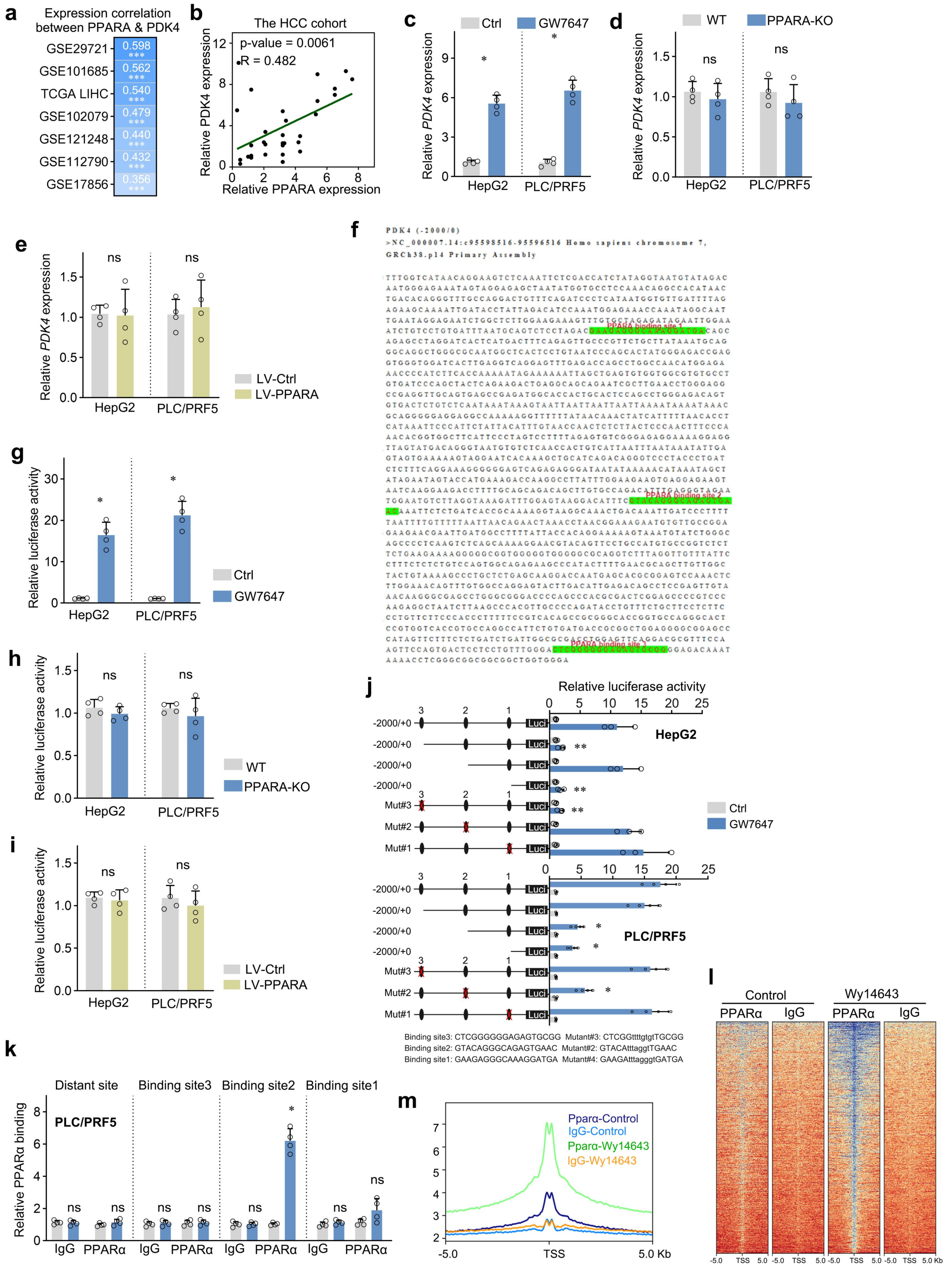

Supplementary Figure 17 Ligand-dependent PPARα activation promotes PDK4 expression. (**a**) Correlations between *PPARA* and *PDK4* expression in GEO and TCGA-LIHC datasets. (**b**) Correlation between *PPARA* and *PDK4* expression in the study HCC cohort (n=31). (**c**) *PDK4* expression after GW7647 treatment (n = 4 independent experiments). (**d**) *PDK4*

expression in *PPARA*-WT and *PPARA*-KO cells (*n* = 4 independent experiments). (**e**) *PDK4* expression in LV-Ctrl- and LV-*PPARA*-transduced cells (*n* = 4 independent experiments). (**f**) Putative PPARα-binding sites in the *PDK4* promoter (*n* = 4 independent experiments); green denotes the indicated sites. (**g-i**) *PDK4* promoter activity after GW7647 treatment, *GSDME* knockout or PPARα overexpression (*n* = 4 independent experiments). (**j**) Luciferase analysis of truncated or mutated *PDK4* promoter constructs in HepG2 and PLC/PRF/5 cells treated with vehicle or GW7647 (*n* = 4 independent experiments). (**k**) PPARα ChIP-qPCR at the *PDK4* promoter. (**l**) PPARα ChIP-seq signal density around transcription start sites with or without Wy14643. (**m**) PPARα ChIP-seq tracks at the *Pdk4* promoter. Data are mean ± s.d.; each point represents one independent experiment or patient. ns not significant, *p <0.05, **p <0.01, ***p <0.001, exact *P* value was provided in the source data. Statistical significance was assessed using two-sided Mann-Whitney U tests in (c-e), (g-i) and (k), and two-sided Pearson correlation tests in (a) and (b). Source data are provided as a Source Data file.

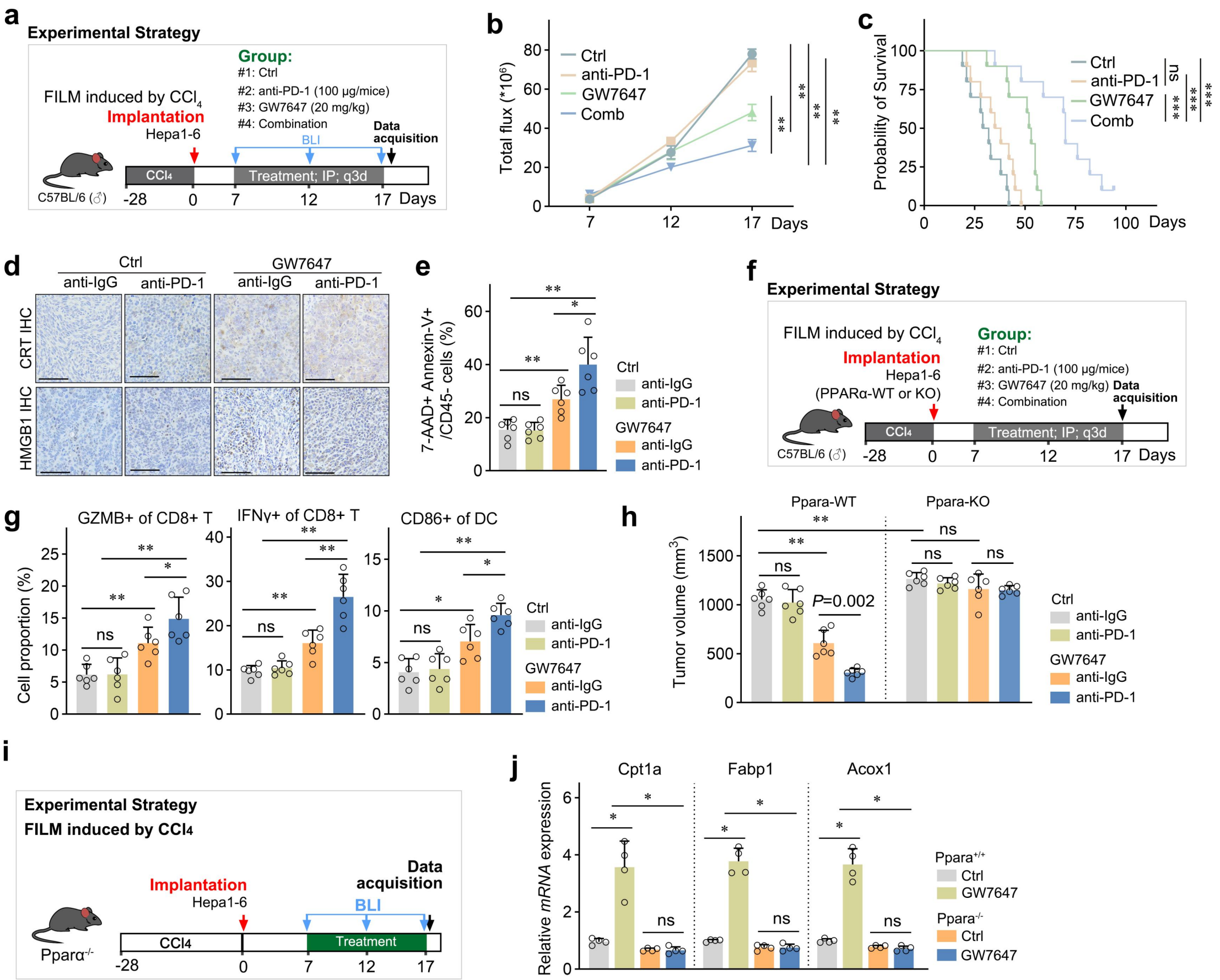

Supplementary Figure 18. Tumor-cell-intrinsic PPARα is required for GW7647-mediated anti-PD-1 sensitization in FILM-associated HCC. (**a**) Experimental scheme for GW7647, anti-PD-1 or combination treatment in CCl_4-induced FILM orthotopic HCC. (**b**) tumor radiance (*n* = 6 mice per group). (**c**) Overall survival (*n* = 10 mice per group). (**d**) Representative CRT and HMGB1 IHC images (*n* = 6 mice per group; scale bars, 100 μm). (**e**) Annexin V^+7-AAD^+CD45^- terminally membrane-disrupted tumor cells (*n* = 6 mice per group). (**f**) Experimental scheme for CCl_4-induced FILM models bearing *PPARA*-WT or *PPARA*-KO Hepa1-6 tumors. (**g**) GZMB+ and IFN γ + CD8+ T cells and CD86+ dendritic cells (n = 6 mice per group). (**h**) Tumor volumes in the indicated groups (*n* = 6 mice per group). (**i**) Experimental scheme for CCl_4-induced FILM models in *Ppara*-/- recipients bearing PPARα-intact Hepa1-6 tumors. (**j**) *Cpt1a*, *Fabp1* and *Acox1* expression in *Ppara*+/+ and *Ppara*-/-

mouse tissues after vehicle or GW7647 treatment (n = 6 mice per group). Data are mean ± s.d.; each point represents one biologically independent mouse. ns not significant, *p <0.05, **p <0.01, ***p <0.001, exact *P* value was provided in the source data. Statistical significance was assessed using Kruskal-Wallis tests in (b), (e), (g), (i) and (k), and a two-sided log-rank Mantel-Cox test in (c). Source data are provided as a Source Data file.

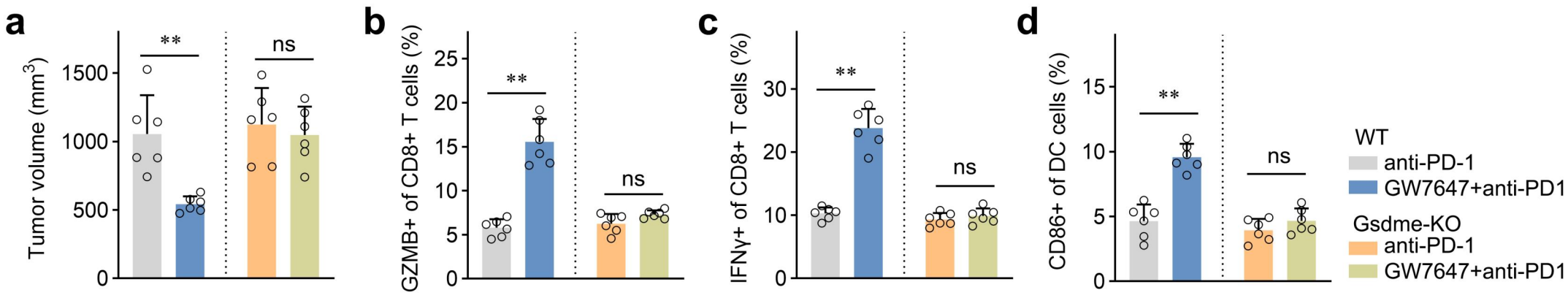

Supplementary Figure 19. Tumor-cell-intrinsic GSDME is required for PPARα agonist-mediated enhancement of anti–PD-1 efficacy and immune activation. (**a**) tumor volumes in orthotopic FILM-HCC models established using WT or *GSDME*-KO HCC cells and treated with anti-PD-1 alone or GW7647 plus anti-PD-1. (**b-d**) GZMB$^+$ CD8$^+$ T cells (b), IFNγ$^+$ CD8$^+$ T cells (c) and CD86$^+$ dendritic cells (d) in the indicated groups. For all panels, n = 6 mice per group. Data are mean ± s.d.; each point represents one mouse. ns not significant, *p <0.05, **p <0.01, ***p <0.001, exact *P* value was provided in the source data. Statistical significance was assessed using Kruskal-Wallis tests. Source data are provided as a Source Data file.

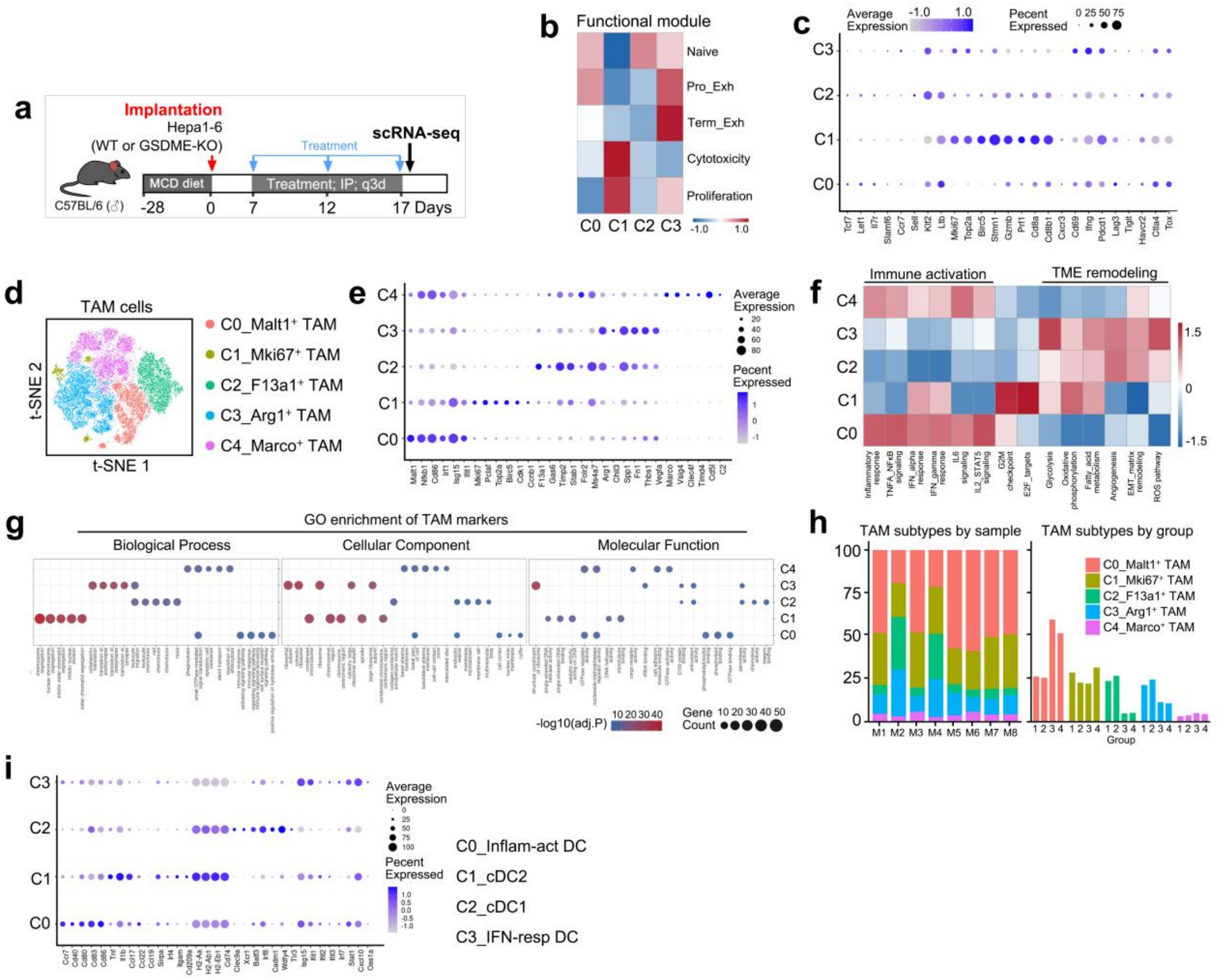

Supplementary Figure 20 CD45⁺ scRNA-seq reveals GSDME-dependent immune remodeling induced by PPARα agonist during anti-PD-1 therapy. (**a**) Experimental scheme, treatment schedule and sample allocation for orthotopic FILM-HCC models; all groups received anti-PD-1. (**b-c**) T-cell reclustering, marker expression, functional scores and subset composition. (**d-h**) tumor-associated macrophage reclustering, marker expression, pathway analysis, Gene Ontology enrichment and subset composition. (**i**) Dendritic-cell reclustering, marker expression and subset composition. Cell numbers, colour scales, symbols and statistical comparisons are defined in-panel. Exact *P* values are shown in the corresponding panels. t-SNE, t-distributed stochastic neighbour embedding. Source data are provided as a Source Data file.

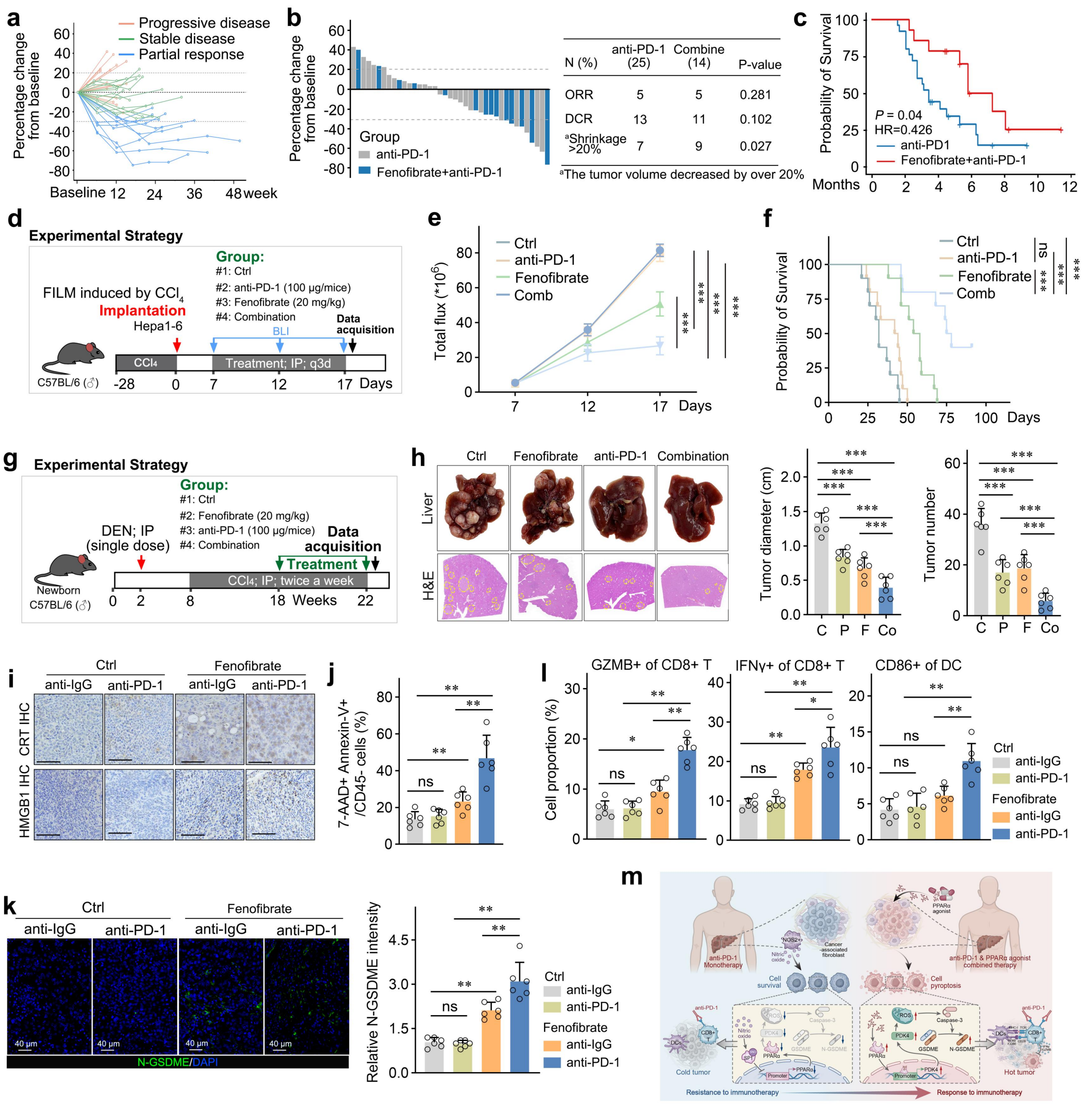

Supplementary Figure 21 Fenofibrate enhances anti-PD-1 efficacy in CCl_4-induced FILM and DEN/CCl_4-induced HCC models. (**a**) Longitudinal changes in tumor burden in patients treated with anti-PD-1 with or without concomitant fenofibrate (Partial response n=10, Progressive disease n=15, Stable disease n=14). (**b**) Best percentage change in tumor burden and summary of objective response rate, disease control rate and >20% tumor shrinkage. (**c**) Progression-free survival according to concomitant fenofibrate use (anti-PD-1 n=25, combined n=14). (**d**) Experimental scheme for fenofibrate, anti-PD-1 or combination treatment in CCl_4-induced FILM orthotopic HCC. (**e**) tumor growth assessed by bioluminescence imaging (n = 6

mice per group). (**f**) Overall survival (n = 10 mice per group). (**g**) Experimental scheme for the DEN-CCl_4-induced HCC model and the indicated treatments. (**h**) Representative gross liver images and H&E staining, tumor diameter and tumor number in the indicated groups (n = 6 mice per group). (**i**) Representative CRT and HMGB1 IHC; scale bars, 100 μm. (**j**) Annexin V^+7-AAD^+CD45^- terminally membrane-disrupted tumor cells (n = 6 mice per group). (**k**) N-GSDME immunofluorescence and quantification (n = 6 mice per group). (**l**) GZMB+ and IFN γ+ CD8+ T cells and CD86+ dendritic cells (n = 6 mice per group). (**m**) Proposed model. Data are mean ± s.d.; each point represents one biologically independent mouse. ns not significant, *p <0.05, **p <0.01, ***p <0.001, exact *P* value was provided in the source data. Statistical significance was assessed using Kruskal-Wallis tests in (e), (h), (j), (k) and (l), and a two-sided log-rank Mantel-Cox test in (c) and (f). Source data are provided as a Source Data file.

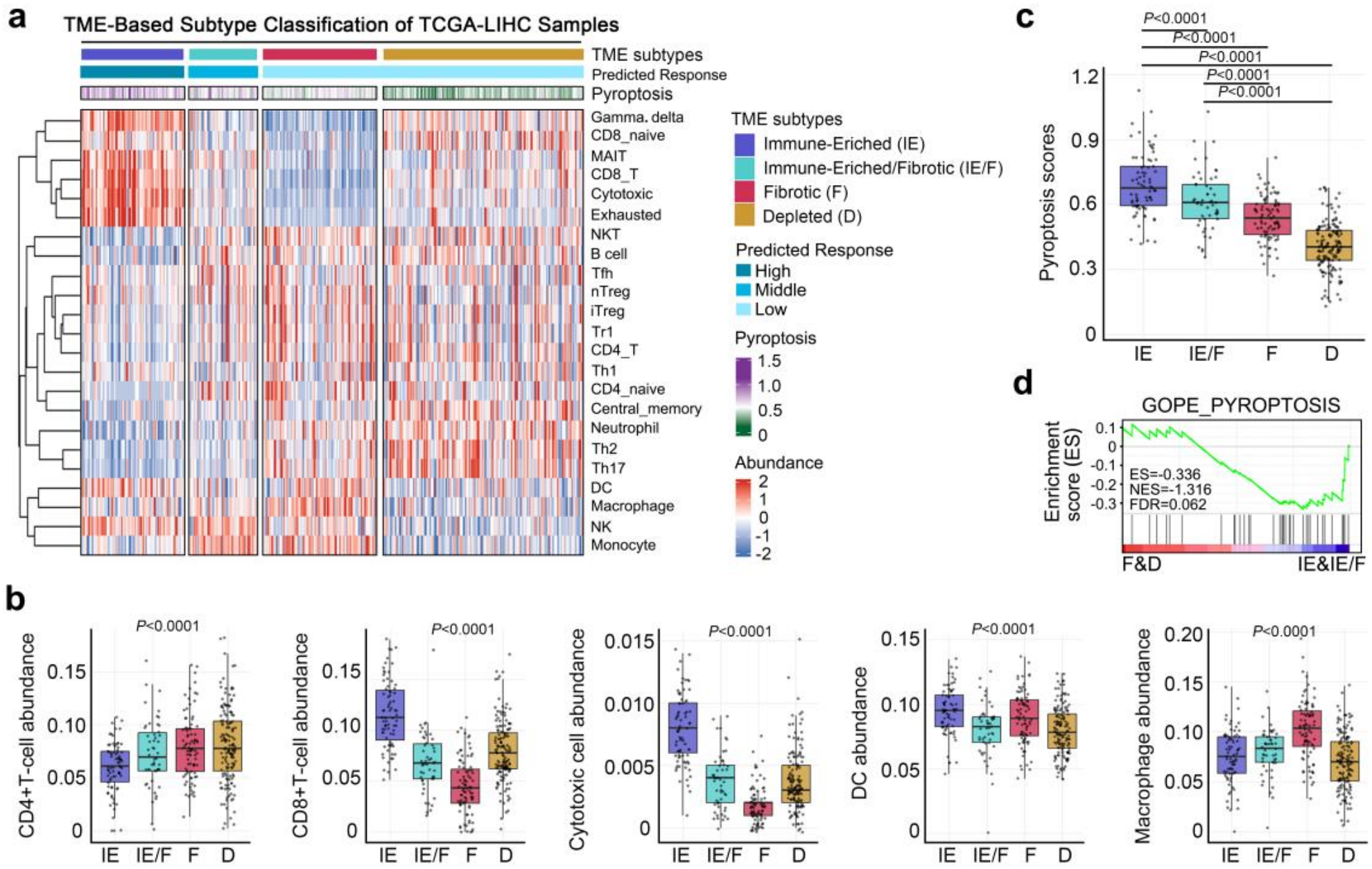

Supplementary Figure 22 TME-Based Subtype Classification and Pyroptosis Signature Analysis in TCGA-LIHC Samples. (**a**) Heatmap showing TME-based subtype classification of TCGA-LIHC samples including Immune-Enriched (IE, n=75), Immune-Enriched/Fibrotic (IE/F, n=50), Fibrotic (F, n=84), and Depleted (D, n=148). Predicted responses to immunotherapy are indicated as high, middle, or low (https://science.bostongene.com/tumor-portrait/samples). The heatmap highlights the pyroptosis signatures calculated by ssGSEA and immune cell abundance obtained by ImmuneCellAI, with color intensity indicating relative abundance. (**b**) Boxplots comparing the abundance of specific immune cell populations across the four TME subtypes. (**c**) Boxplot showing the distribution of pyroptosis scores across the four TME subtypes. (**d**) GSEA of pyroptosis signatures (GOPE_PYROPTOSIS) across TME subtypes.

Supplementary Table 1. Clinical Characteristics of 147 HCC Patients

Variable	Number of patients			
	**FILM** (*n*=81)	**Fibrosis-dominant** (*n*=44)	**Inflammation-dominant** (*n*=11)	**Control** (*n*=11)
**Age, years**				
≤50	40	25	5	6
>50	41	19	6	5
**Gender**				
Female	2	6	4	6
Male	79	38	7	5
**Hepatitis B infection**				
No	2	4	6	8
Yes	79	40	5	3
**ALT (U/L)**				
≤40	9	36	1	11
>40	72	8	10	0
**Cirrhosis**				
No	0	0	11	11
Yes	81	44	0	0
**Tumor size (cm)**				
≤5	30	20	9	11
>5	51	24	2	0
**Tumor number**				
Single	60	35	9	11
Multiple	21	9	2	0
**AFP (ng/ml)**				
≤400	20	16	4	5
>400	61	28	7	6

Supplementary Table 2. Clinical Characteristics of 16 HCC Patients who underwent surgery after PD-1 inhibitor treatment.

Variable	Number of patients			
	**PD** (*n*=1)	**SD** (*n*=9)	**PR** (*n*=6)	**CR** (*n*=0)
**Age, years**				
≤50	0	3	3	0
>50	1	6	3	0
**Gender**				
Female	0	1	2	0
Male	1	8	4	0
**Hepatitis B infection**				
No	0	2	1	0
Yes	1	7	5	0
**Tumor size (cm)**				
≤5	0	3	2	0
>5	1	6	4	0
**Tumor number**				
Single	0	4	3	0
Multiple	1	5	3	0
**AFP (ng/ml)**				
≤400	0	3	1	0
>400	1	6	5	0
**BCLC**				
A	0	4	3	0
B	1	5	3	0
**PD-1 mAbs**				
Camrelizumab	0	2	2	0
Sintilimab	0	0	1	0
Tislelizumab	0	1	1	0
Nivolumab	0	3	1	0
Pembrolizumab	1	3	1	0

Supplementary Table 3. Clinical characteristics of 39 patients with HCC treated with anti-PD-1 therapy with or without concomitant fenofibrate

Variable	Number of patients		
	PD-1 mAb (*n*=25)	PD-1 mAb with fenofibrate (*n*=14)	*P*-value
**Age**			0.109
≤50	3 (12.0%)	5 (35.7%)	
>50	22 (88.0%)	9 (64.3%)	
**Gender**			1
Female	6 (24.0%)	4 (28.6%)	
Male	19 (76.0%)	10 (71.4%)	
**Hepatitis B infection**			0.686
No	4 (16.0%)	3 (21.4%)	
Yes	21 (84.0%)	11 (78.6%)	
**ECOG score**			0.514
0	11 (44.0%)	8 (57.1%)	
1	14 (56.0%)	6 (42.9%)	
**Child-Pugh**			1
A	19 (76.0%)	11 (78.6%)	
B	6 (24.0%)	3 (21.4%)	
**Tumor number**			0.74
Single	9 (36.0%)	6 (42.9%)	
Multiple	16 (64.0%)	8 (57.1%)	
**Tumor size (cm)**			0.225
≤5	3 (12.0%)	4 (28.6%)	
>5	22 (88.0%)	10 (71.4%)	
**BCLC**			0.664
A	1 (4.0%)	2 (14.3%)	
B	7 (28.0%)	3 (21.4%)	
C	17 (68.0%)	9 (64.3%)	
**AFP (ng/ml)**			0.518
≤400	12 (48.0%)	5 (35.7%)	
>400	13 (52.0%)	9 (64.3%)	

Supplementary Table 4. Sequences of primers

Primer name		Sequence
h-GSDMA	Forward	TACGTCCGCACCGACTACA
	Reverse	CAGAGTGCTGTTCTGCGAGA
h-GSDMB	Forward	TGATTGCCGTTAGAAGCCTTG
	Reverse	TCCCGTTGAGTCTACATTATCCA
h-GSDMC	Forward	TCCATGTTGGAACGCATTAGC
	Reverse	CAAACTGACGTAATTTGGTGGC
h-GSDMD	Forward	GTGTGTCAACCTGTCTATCAAGG
	Reverse	CATGGCATCGTAGAAGTGGAAG
h-GSDME	Forward	ACATGCAGGTCGAGGAGAAGT
	Reverse	TCAATGACACCGTAGGCAATG
*h-PPAR*α	Forward	TTCGCAATCCATCGGCGAG
	Reverse	CCACAGGATAAGTCACCGAGG
h-PPARG	Forward	ACCAAAGTGCAATCAAAGTGGA
	Reverse	ATGAGGGAGTTGGAAGGCTCT
h-PPARD	Forward	CAGGGCTGACTGCAAACGA
	Reverse	CTGCCACAATGTCTCGATGTC
h-PDK4	Forward	GGAGCATTTCTCGCGCTACA
	Reverse	ACAGGCAATTCTTGTCGCAAA
h-NOS2	Forward	TCATCCGCTATGCTGGCTAC
	Reverse	CCCGAAACCACTCGTATTTGG
h-APOA4	Forward	CCCAGCAACTCAATGCCCT
	Reverse	CCTTCAGTTTCTCCGAGTCCT
h-CYP1B1	Forward	TGAGTGCCGTGTGTTTCGG
	Reverse	GTTGCTGAAGTTGCGGTTGAG
h-GAPDH	Forward	GGAGCGAGATCCCTCCAAAAT
	Reverse	GGCTGTTGTCATACTTCTCATGG
m-Gsdma	Forward	ACGCGGGGAGAACCTCTAT
	Reverse	GCCGTTGACCATCAGTTGTCT
m-Gsdmc	Forward	CCAACTGCTCGAAGCTAGAGA
	Reverse	AGCGCACGGTCATTTCCAG
m-Gsdmd	Forward	TTCCAGTGCCTCCATGAATGT

	Reverse	GCTGTGGACCTCAGTGATCT
m-Gsdme	Forward	TGCAACTTCTAAGTCTGGTGACC
	Reverse	CTCCACAACCACTGGACTGAG
*m-Ppar*α	Forward	TTTCGGCGAACTATTCGGCTG
	Reverse	GGCATTTGTTCCGGTTCTTCTT
m-Pparg	Forward	GGAAGACCACTCGCATTCCTT
	Reverse	GTAATCAGCAACCATTGGGTCA
m-Pdk4	Forward	AGGGAGGTCGAGCTGTTCTC
	Reverse	GGAGTGTTCACTAAGCGGTCA
m-Nos2	Forward	GTTCTCAGCCCAACAATACAAGA
	Reverse	GTGGACGGGTCGATGTCAC
m-Gapdh	Forward	AGGTCGGTGTGAACGGATTTG
	Reverse	TGTAGACCATGTAGTTGAGGTCA
PDK4 site #1	Forward	GCTCTTGGAAGAAAGTTTGTGCT
	Reverse	CCTAGGCTCTGCTGTCATCC
PDK4 site#2	Forward	TGTGCCAGACATTTGAGGGT
	Reverse	TGCCTTACCTTTTGCGGTGA
PDK4 site#3	Forward	GATCTGATTGGCGCGACCT
	Reverse	GCCGCCCGAGGTTTTATTTG

h represents human, m represents mouse

Supplementary Table 5. Primary antibodies for WB, IHC, IF, multiplex IF, and related assays

Antibody	Application	Supplier	Catalog No.	Clone	Dilution
PPARα	WB	Abcam	ab126285	Polyclonal	1：1000
PPARα	IHC/IF	Abcam	ab126285	Polyclonal	1：200
PDK4	WB	Proteintech	12949-1-AP	Polyclonal	1：1000
PDK4	IHC	Proteintech	12949-1-AP	Polyclonal	1：100
HMGB1	IHC	Abcam	ab18256	Polyclonal	1：200
Calreticulin	IHC	Abcam	ab92516	Monoclonal	1：200
PPARγ	WB	Abcam	ab45036	Polyclonal	1：1000
PPARδ	WB	Abcam	Ab23673	Polyclonal	1：1000
GSDMA	WB	Cell Signaling Technology	49307	Polyclonal	1：1000
GSDMC	WB	Cell Signaling Technology	61921	Monoclonal	1：1000
GSDMD	WB	Cell Signaling Technology	39754	Monoclonal	1：1000
GSDME	WB	Cell Signaling Technology	19453	Monoclonal	1：1000
Cleaved caspase-3	WB	Cell Signaling Technology	9661	Not specified	1：1000
PARP1	WB	Cell Signaling Technology	9532	Monoclonal	1：1000
p-PDH E1α	WB	Proteintech	29582-1-AP	Polyclonal	1：1000
PDH E1α	WB	Proteintech	18068-1-AP	Polyclonal	1：1000
SP1	WB	Cell Signaling Technology	9389	Monoclonal	1：1000
SP1	ChIP-qPCR	Cell Signaling Technology	9389	Monoclonal	1：100
Flag	WB	Sigma	F1804	Monoclonal	1：1000
GAPDH	WB	Cell Signaling Technology	2118	Monoclonal	1：1000

**Antibody**	**Application**	**Supplier**	**Catalog No.**	**Clone**	**Dilution**
β-actin	WB	Cell Signaling Technology	4970	Monoclonal	1：1000
α-tubulin	WB	Cell Signaling Technology	3873	Monoclonal	1：1000
LAMB1	WB	Proteintech	23498-1-AP	Polyclonal	1：1000
CD8	Multiplex IF	Abcam	ab237709	Monoclonal	1:100
CD11c	Multiplex IF	Cell Signaling Technology	97585	Monoclonal	1:200
α-SMA	Multiplex IF	Abcam	ab242395	Monoclonal	1:500
F4/80	Multiplex IF	Abcam	ab300421	Monoclonal	1:200
CD68	Multiplex IF	Abcam	ab283654	Monoclonal	1:100
iNOS	Multiplex IF	Abcam	ab178945	Monoclonal	1:100
N-GSDME	IF	Abcam	ab256122	Monoclonal	1:100
Ki67	IHC	Abcam	ab16667	Monoclonal	1:200
Anti-rabbit IgG (HRP)	WB secondary	Cell Signaling Technology	7074	N/A	1：2000
Anti-mouse IgG (H+L)	WB secondary	Cell Signaling Technology	7076	N/A	1：2000

Supplementary Table 6. Flow cytometry antibodies

**Target**	**Fluorophore**	**Supplier**	**Catalog No.**	**Clone**	**Dilution** (per 1 × 10^6 cells in 100 µl)
CD16/32 Fc block	Unconjugated	BioLegend	#156603	S17011E	1 µl
CD45	PerCP/Cy5.5	BioLegend	#103132	30-F11	1 µl
CD4	FITC	BioLegend	#100406	GK1.5	2 µl
CD8a	PE/Cy7	BioLegend	#162312	S18018E	1 µl
CD11b	BV510	BD Pharmingen	562950	Not specified	2 µl
CD11c	BV605	BioLegend	#117334	N418	2 µl
NK1.1	APC	BioLegend	#156506	S17016D	2 µl
F4/80	BV421	BioLegend	#123137	BM8	1 µl
I-A/I-E	BV785	BioLegend	#107645	M5/114.15.2	1 µl
Gr-1	PE	BD Pharmingen	553128	RB6-8C5	1 µl
CD80	APC	BioLegend	#104714	16-10A1	1 µl
CD86	BV421	BioLegend	#105123	PO3	2 µl
IFN-γ	Alexa Fluor 700	BioLegend	#505823	XMG1.2	2 µl
Granzyme B	BV421	BioLegend	#396414	QA18A28	2 µl

Supplementary Table 7. In vivo treatment, and depletion antibodies

Antibody	Supplier	Catalog No.	Clone	Schedule
anti-PD-1 mAb	BioXCell	BE0146	RMP1-14;rat IgG2a, κ	100 µg/mouse, i.p., q3d
anti-CSF1R	BioXCell	BE0213	AFS98; rat IgG2a κ	300 µg/mouse, i.p., q3d
anti-CD8α	BioXCell	BE0061	2.43; rat IgG2b, κ	200 µg/mouse; i.p.; q3d
anti-CD4	BioXCell	BE0003-1	GK1.5; rat IgG2b, κ	200 µg/mouse; i.p.; q3d
anti-NK1.1	BioXCell	BE0036	PK136; mouse IgG2a, κ	200 µg/mouse; i.p.; q3d
anti-Ly6G	BioXCell	BE0075-1	1A8; rat IgG2a, κ	200 µg/mouse; i.p.; q3d
anti-CD20	BioXCell	BE0356	MB20-11; mouse IgG2c, κ	250 µg/mouse; i.p.; q5d
anti-CD11c	BioXCell	BE0038	N418; Armenian hamster IgG2	200 µg/mouse; i.p.; q3d

Supplementary Table 8. siRNA sequence

siRNA	Target gene	Sense sequence, 5′→3′
siNC	Negative control	TTCTCCGAACGTGTCACGT
siCPT1A	CPT1A	GCAAGCACAUCGUCGUGUA
siAPOA4	APOA4	AGGACUCGGAGAAACUGAA
siCYP1B1	CYP1B1	GCAUGAUGCGCAACUUCUU
siEp300	Ep300	CCAGCCTCAAACTACAATAAA
siFoxa2	Foxa2	CTACGCCAACATGAACTCGAT
siHnf4a	Hnf4a	GACAATGTGTGGTAGACAAAG
siMxi1	Mxi1	TTGGAAAGAGTACAGTTTCTAAC
siRxra	Rxra	GTCCTTATGGGCCCAAAAGATGC
siSin3a	Sin3a	GCTGGTGTACCTGTTGTCAAA
siTaf1	Taf1	TTCTATCTTGGAGTCTATTAT
siYy1	Yy1	CCCTAAGCAACTGGCAGAATT
siZeb1	Zeb1	GCATCATTTGATTGAGCACAT

Supplementary Table 9. Pharmacological compounds, inhibitors and donors

Reagent	Supplier	Catalog No.
GW7647	MedChemExpress	HY-13861
Fenofibrate	MedChemExpress	HY-17356
C-PTIO	MedChemExpress	HY-18734A
L-NIL/NIL	MedChemExpress	HY-12116
SNAP	MedChemExpress	HY-121526
z-VAD-FMK	MedChemExpress	HY-16658B
z-DEVD-FMK	MedChemExpress	HY-12466
Ac-DEVD-CHO	MedChemExpress	HY-P1001
VX-765	MedChemExpress	HY-13205
z-LEVD-FMK	MedChemExpress	HY-128707
NAC	MedChemExpress	HY-B0215
MitoTEMPO	MedChemExpress	HY-112879
Etomoxir	MedChemExpress	HY-50202
GSK2606414	MedChemExpress	HY-18072
4μ8C	MedChemExpress	HY-19707
MitoSOX Red	MedChemExpress	HY-D1055
Clodronate liposomes	MedChemExpress	HY-172202
CCl_4	Macklin	KAW425
DEN	Sigma	N0756

Supplementary Table 10. Reporter and promoter constructs

Construct	Backbone	Mutation	Supplier
pGL3-PDK4 promoter −2000/0	pGL3-Basic	None	This study
pGL3-PDK4 −1945/0	pGL3-Basic	None	This study
pGL3-PDK4 −1188/0	pGL3-Basic	None	This study
pGL3-PDK4 −307/0	pGL3-Basic	None	This study
PDK4 promoter Mut1	pGL3-Basic	Binding site 1 mutation	This study
PDK4 promoter Mut2	pGL3-Basic	Binding site 2 mutation	This study
PDK4 promoter Mut3	pGL3-Basic	Binding site 3 mutation	This study
pRL-TK	Renilla control plasmid	/	Promega
PPRE-luciferase reporter	PPAR response element reporter	/	Promega

Supplementary Table 11. shRNAs, CRISPR sgRNAs, lentiviral vectors, and AAV vectors.

Construct	Target	Source	Sequence
shPDK4	Human PDK4 knockdown	This study	AACATTCTGAAGGAAATTGATAT
shPdk4	Mouse Pdk4 knockdown	This study	CTGGACTTTGGTTCAGAAAATGC
sgGsdme	Mouse Gsdme knockout	This study	CGGGGCTATTGGGACAGTCG
sgGSDME	Human GSDME knockout	This study	GTATAACTCAATGACACCGTAGG
sgPparα	Mouse Pparα knockout	This study	TCTGTCGGGATGTCACACAA
sgPPARα	Human PPARα knockout	This study	AGCGTGGACGAGTCTCCCAGTGG
LV-Ctrl	Control lentivirus	This study	Empty vector
LV-PPARA	Human PPARα overexpression	This study	Human PPARα cDNA
LV-PPARA	Mouse Pparα overexpression	This study	Mouse Pparα cDNA
LV-PDK4	Human PDK4 overexpression	This study	Human PDK4 cDNA
LV-Flag-N-Gsdme	Flag-tagged N-GSDME	This study	N-terminal fragment of mouse GSDME
sgPDK4	PDK4 knockout	This study	GAGGCGGTCAATAATTCTCAGGG
PDK4-WT rescue	Wild-type PDK4	This study	PDK4-WT cDNA
PDK4-KD rescue	Kinase-dead PDK4 mutant	This study	D394A/W395A
pMD2.G	Lentiviral packaging plasmid	addgene	/
psPAX2	Lentiviral packaging plasmid	addgene	/
AAV8-F4/80-shCtrl	/	HanBio	empty vector
AAV8-F4/80-shNos2	Nos2	HanBio	AAAGAACACCACTTTCACCAA

Uncropped western blot images corresponding to Supplementary Figure 2

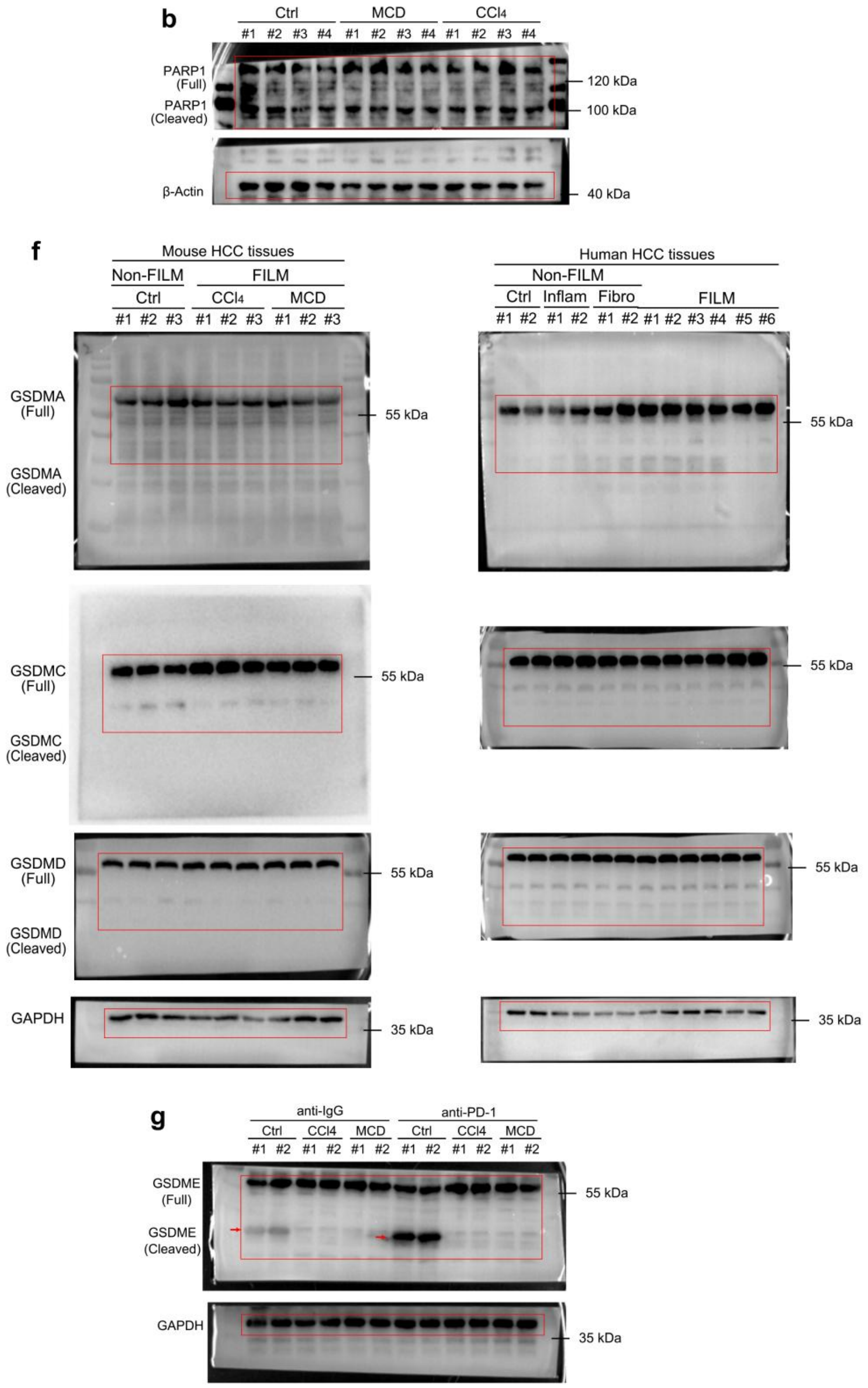

Uncropped western blot images corresponding to Supplementary Figure 3

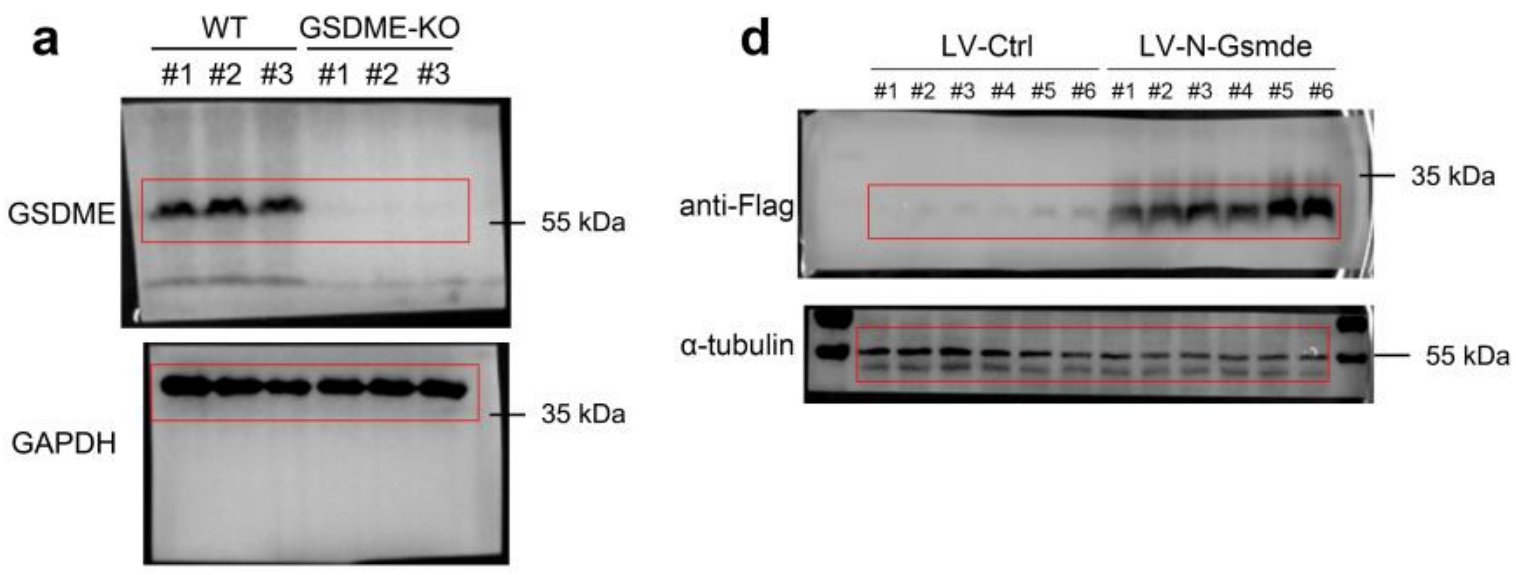

Uncropped western blot images corresponding to Supplementary Figure 6

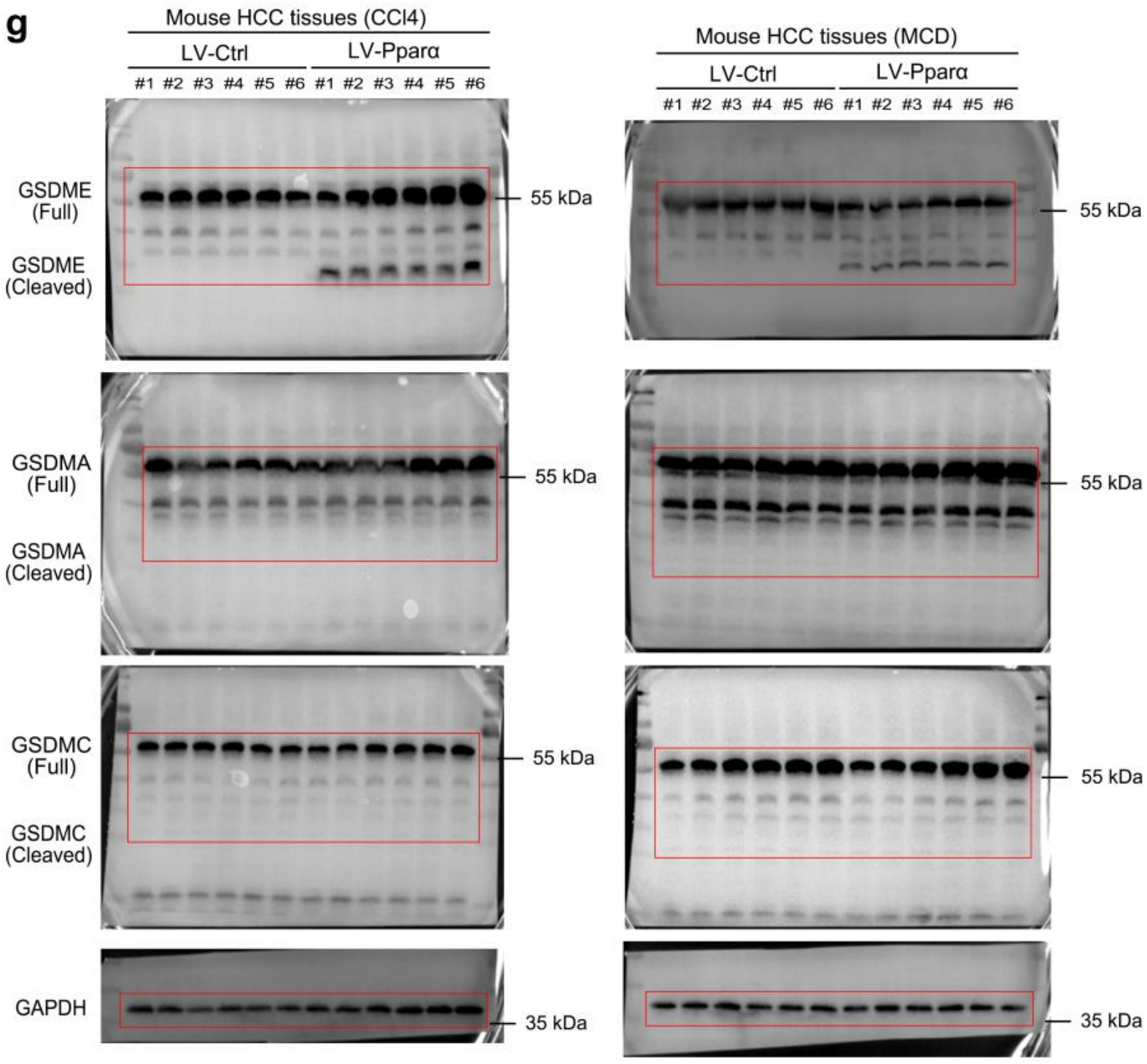

Uncropped western blot images corresponding to Supplementary Figure 7

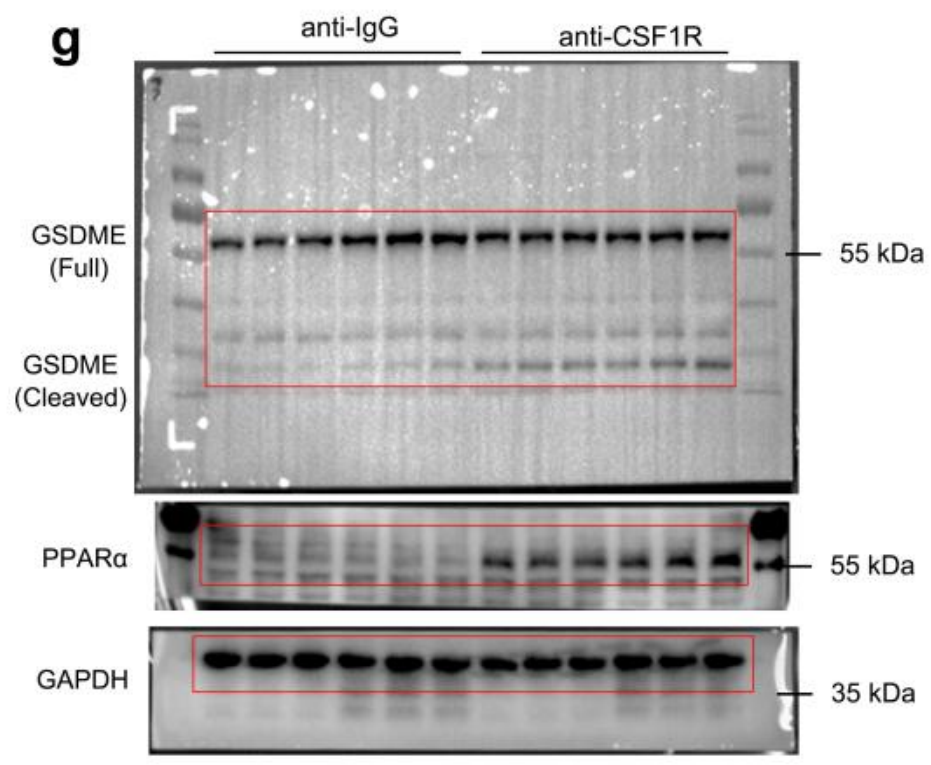

Uncropped western blot images corresponding to Supplementary Figure 11

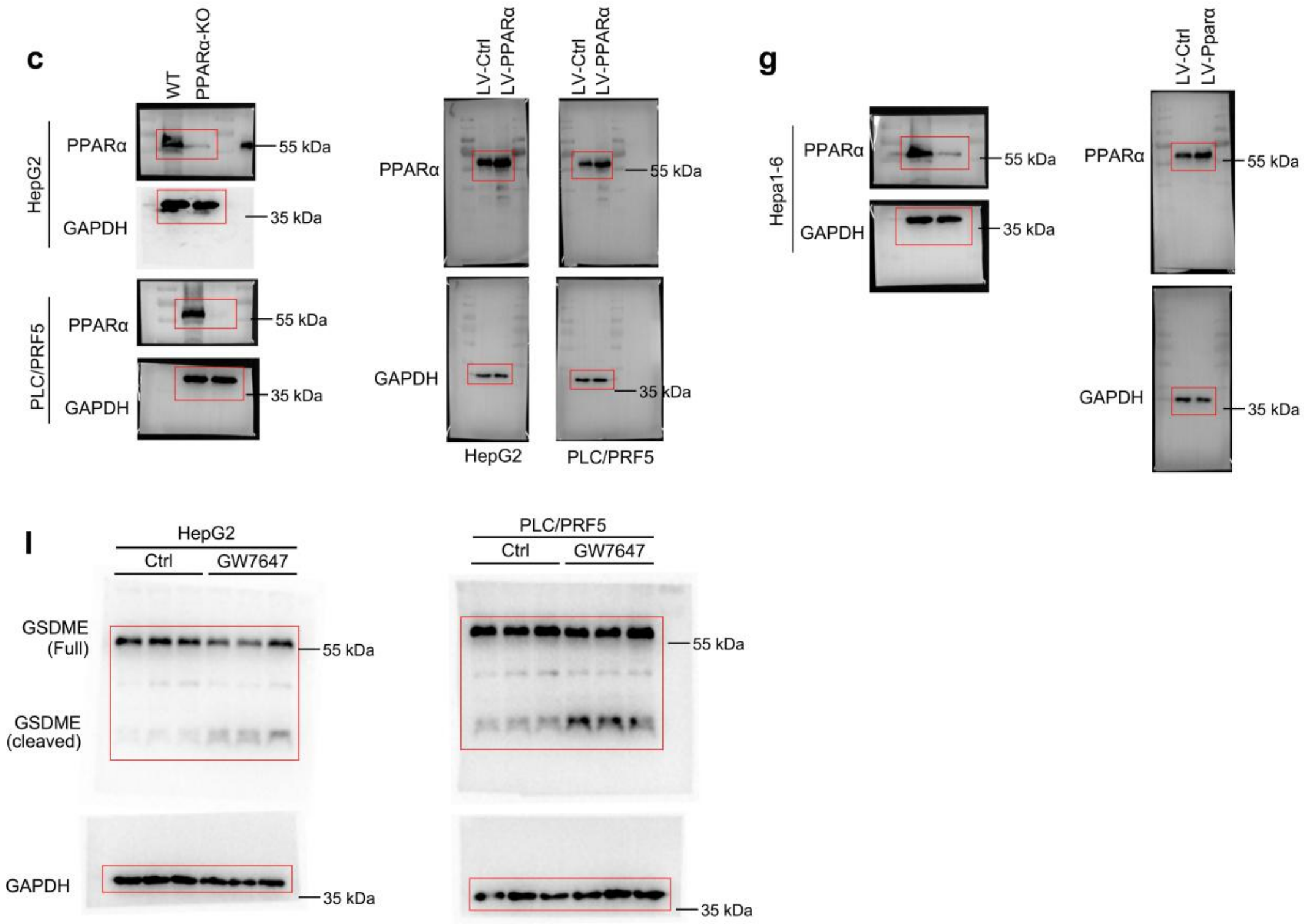

Uncropped western blot images corresponding to Supplementary Figure 12

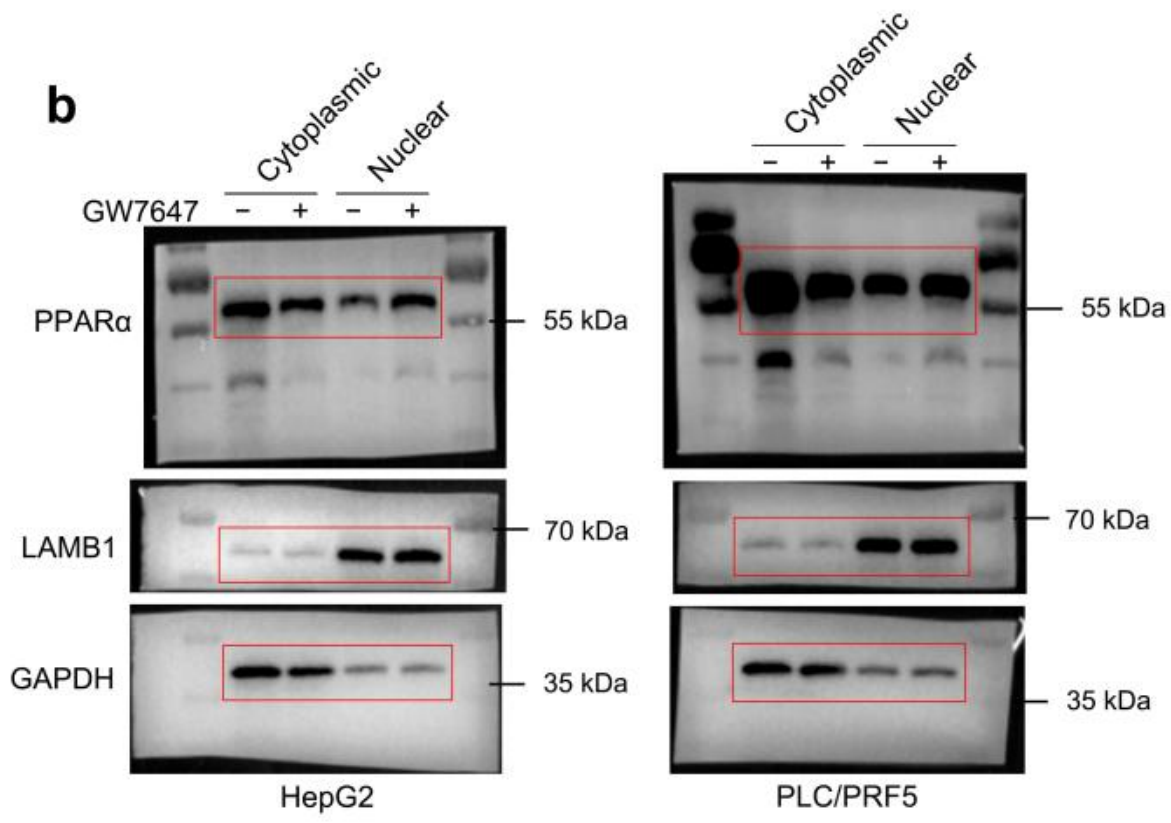

Uncropped western blot images corresponding to Supplementary Figure 14

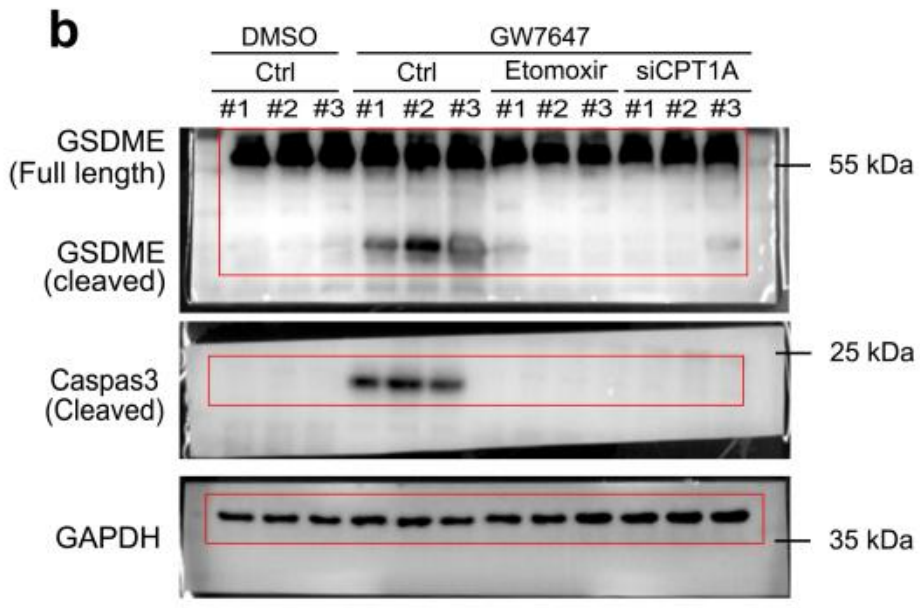

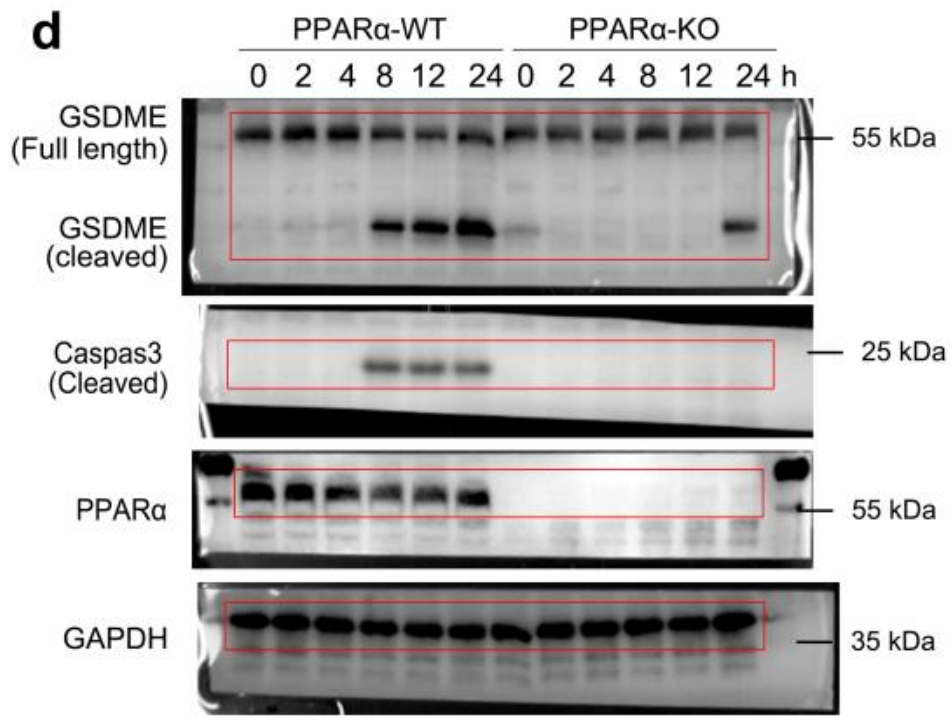

Uncropped western blot images corresponding to Supplementary Figure 16

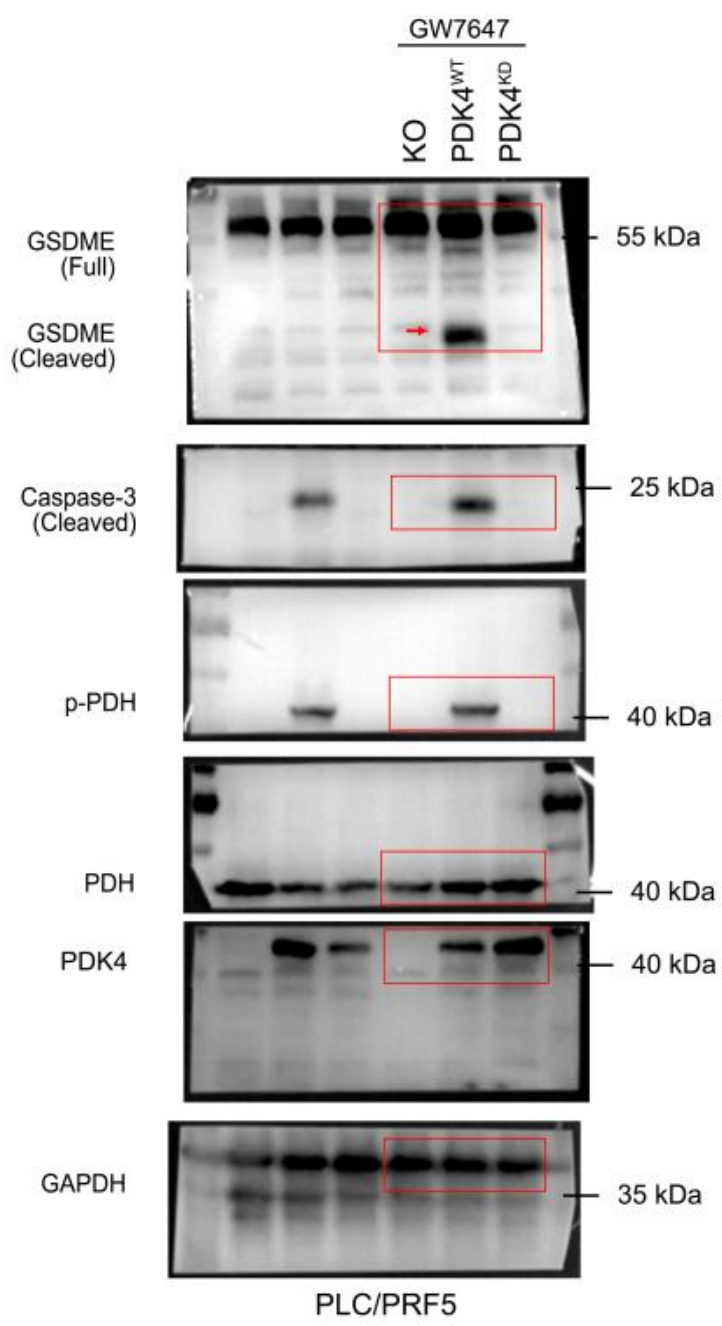

Supplement: Supplementary file 1 [file 41467_2026_75770_MOESM1_ESM.pdf]
