# Supplementary material for: PPARα activation overcomes fibroinflammatory liver microenvironment-associated anti-PD-1 resistance in hepatocellular carcinoma by mediating GSDME-dependent pyroptosis
Source: Nat Commun. 2026 Aug 6;17:9474. doi: 10.1038/s41467-026-75770-7 (PMC13542117; doi:10.1038/s41467-026-75770-7)
Supplement: Supplementary file 2 [file 41467_2026_75770_MOESM2_ESM.pdf]

nature portfolio

Corresponding author(s): Yuchuan Jiang

Last updated by author(s): May 24, 2026

Reporting Summary

Nature Portfolio wishes to improve the reproducibility of the work that we publish. This form provides structure for consistency and transparency in reporting. For further information on Nature Portfolio policies, see our Editorial Policies and the Editorial Policy Checklist.

Statistics

For all statistical analyses, confirm that the following items are present in the figure legend, table legend, main text, or Methods section.

n/a	Confirmed	
[ ]	[x]	The exact sample size (*n*) for each experimental group/condition, given as a discrete number and unit of measurement
[ ]	[x]	A statement on whether measurements were taken from distinct samples or whether the same sample was measured repeatedly
[ ]	[x]	The statistical test(s) used AND whether they are one- or two-sided<br>*Only common tests should be described solely by name; describe more complex techniques in the Methods section.*
[x]	[ ]	A description of all covariates tested
[ ]	[x]	A description of any assumptions or corrections, such as tests of normality and adjustment for multiple comparisons
[ ]	[x]	A full description of the statistical parameters including central tendency (e.g. means) or other basic estimates (e.g. regression coefficient) AND variation (e.g. standard deviation) or associated estimates of uncertainty (e.g. confidence intervals)
[ ]	[x]	For null hypothesis testing, the test statistic (e.g. *F*, *t*, *r*) with confidence intervals, effect sizes, degrees of freedom and *P* value noted<br>*Give P values as exact values whenever suitable.*
[x]	[ ]	For Bayesian analysis, information on the choice of priors and Markov chain Monte Carlo settings
[x]	[ ]	For hierarchical and complex designs, identification of the appropriate level for tests and full reporting of outcomes
[ ]	[x]	Estimates of effect sizes (e.g. Cohen's *d*, Pearson's *r*), indicating how they were calculated

Our web collection on statistics for biologists contains articles on many of the points above.

Software and code

Policy information about availability of computer code

Data collection	Flow-cytometry data were acquired using a Fortessa flow cytometer operated with BD FACSDiva software version 9.0.1 (BD Biosciences, San Jose, CA, USA). Bulk RNA-seq libraries were sequenced on an Illumina NovaSeq 6000 platform by Gene Denovo Biotechnology Co. (Guangzhou, China). RNA quality was assessed using an Agilent 2100 Bioanalyzer (Agilent Technologies, Palo Alto, CA, USA). Immunohistochemical images were captured using an upright microscope, and multiplex immunohistochemistry images were scanned using the Vectra 3 Imaging System (PerkinElmer, USA). Static bright-field images of apoptotic and pyroptotic cells were captured using a Leica XSP-8CA microscope. Luciferase activity was measured using a Modulus TD20/20 luminometer (Turner BioSystems, USA). In vivo tumor bioluminescence imaging was performed using an FX Pro in vivo imaging system (Bruker, Germany). qPCR data were collected using a LightCycler 480 II system (Roche, USA). Western blot chemiluminescent images were acquired using an enhanced chemiluminescent imaging system (Sage Creation, Beijing, China).
Data analysis	Flow-cytometry data were analyzed using FlowJo version 10.8.2 (BD Biosciences). Multiplex immunohistochemistry images were analyzed using inForm v2.4 software. BFor bulk RNA-sequencing analysis, raw reads were filtered using fastp version 0.23.4 and aligned to the corresponding reference genome using HISAT2 version 2.2.1. Transcript abundance was quantified using StringTie version 2.2.1 and expressed as fragments per kilobase of transcript per million mapped reads. Values were converted to transcripts per million where required. Differential-expression analysis was performed using DESeq2 version 1.42.0. Immune-cell deconvolution analyses were performed using xCell, MCP-counter, quanTIseq and ImmuCellAI. Gene-set enrichment analysis was performed using GSEA version 4.3.3 and Kyoto Encyclopedia of Genes and Genomes gene sets obtained from the Molecular Signatures Database version 2023.2.Hs. P values were adjusted for multiple testing using the Benjamini–Hochberg procedure. Single-cell RNA-sequencing data were analysed in R version 4.3.2 using Seurat version 5.0.1 for quality control, normalization, dimensionality reduction, clustering and differential-expression analysis. T-cell state scores were calculated using TCellSI version 1.2.0, and functional enrichment analysis was performed using clusterProfiler version 4.10.0. Spatial transcriptomic data were analysed using RCTD implemented in spacexr version 2.2.0 for cell-type deconvolution and visualized using ggplot2 version 3.5.1.

Statistical analyses were performed using GraphPad Prism version 9.0, IBM SPSS Statistics version 27.0 and R version 4.3.2. Details of the statistical tests are provided in the Methods and corresponding figure legends.

For manuscripts utilizing custom algorithms or software that are central to the research but not yet described in published literature, software must be made available to editors and reviewers. We strongly encourage code deposition in a community repository (e.g. GitHub). See the Nature Portfolio guidelines for submitting code & software for further information.

Data

Policy information about availability of data

All manuscripts must include a data availability statement. This statement should provide the following information, where applicable:

- Accession codes, unique identifiers, or web links for publicly available datasets
- A description of any restrictions on data availability
- For clinical datasets or third party data, please ensure that the statement adheres to our policy

Human bulk RNA-seq data generated in this study have been deposited in the Genome Sequence Archive for Human (GSA-Human) under accession number HRA019045 (https://ngdc.cncb.ac.cn/gsa-human/browse/HRA019045). These data are available under restricted access because they contain human-derived sequencing data linked to patient clinical information, and access is restricted to protect patient privacy and comply with ethical requirements. The restricted dataset includes raw and processed human bulk RNA-seq data and associated de-identified clinical metadata required to interpret the analyses. Access may be requested through the GSA-Human access system for scientific research purposes. Requests should include a brief research proposal, intended use of the data, applicant affiliation and evidence of institutional or ethics approval where applicable. Access requests will be reviewed by the relevant data access committee and corresponding authors. Requests will be reviewed within 7 days, and approved data will be made available within 2 weeks. Access will be granted for the approved research purpose and for the period permitted by the data access agreement and applicable institutional requirements. Mouse bulk RNA-seq data have been deposited in the Genome Sequence Archive under accession number CRA044550 (https://ngdc.cncb.ac.cn/gsa/browse/CRA044550). The raw and processed single-cell RNA-seq data generated in this study have been deposited in the Genome Sequence Archive under accession number CRA043152 (https://ngdc.cncb.ac.cn/gsa/browse/CRA043152). Publicly available datasets used in this study were obtained from TCGA-LIHC via the UCSC Xena Browser (https://xenabrowser.net/), ICGC-LIRI-JP (https://dcc.icgc.org/releases/current/Projects/LIRI-JP), CPTAC (https://proteomic.datacommons.cancer.gov/pdc/study/PDC000198), and the Gene Expression Omnibus (GEO, https://www.ncbi.nlm.nih.gov/geo), including GSE151530, GSE154277 and GSE238264. The remaining data supporting the findings of this study are available within the Article, Supplementary Information and Source Data files. Source data are provided with this paper.

Research involving human participants, their data, or biological material

Policy information about studies with human participants or human data. See also policy information about sex, gender (identity/presentation), and sexual orientation and race, ethnicity and racism.

Reporting on sex and gender	Sex and gender were not considered in our study design.
Reporting on race, ethnicity, or other socially relevant groupings	Race, ethnicity and other socially relevant groupings were not systematically collected or analyzed in this study, because these variables were not part of the prespecified study design and were not used for patient selection, grouping, or outcome assessment.
Population characteristics	The study included three independent HCC cohorts. Cohort 1 comprised 147 patients (129 male and 18 female; 76 aged ≤50 years and 71 aged >50 years; 127 with and 20 without HBV infection). Cohort 2 comprised 16 patients who underwent surgery after anti-PD-1 treatment (13 male and 3 female; 6 aged ≤50 years and 10 aged >50 years; 13 with and 3 without HBV infection). Cohort 3 comprised 39 patients treated with anti-PD-1 alone or anti-PD-1 plus fenofibrate (29 male and 10 female; 8 aged ≤50 years and 31 aged >50 years; 32 with and 7 without HBV infection). Additional covariates included liver function, cirrhosis, tumour size and number, AFP level, BCLC stage, ECOG performance status, Child–Pugh class and treatment regimen, as applicable. Full characteristics are provided in Supplementary Tables 1–3.
Recruitment	For cohort 1, archived HCC specimens were obtained from patients who underwent surgical resection at Nanfang Hospital between 2006 and 2010 and were histopathologically and clinically diagnosed with HCC. Paired adjacent non-tumor liver tissues were collected at a standard distance from the resected tumor tissues and confirmed by pathological evaluation. For cohort 2, patients with HCC who underwent surgical resection after receiving anti–PD-1 therapy between January 2020 and January 2023 were included, and treatment response was assessed according to iRECIST based on preoperative imaging and pathological materials. For cohort 3, patients with clinically or pathologically diagnosed HCC who received anti–PD-1-based systemic therapy with or without concomitant fenofibrate between January 2020 and January 2024 were retrospectively enrolled. Eligible patients had documented anti–PD-1 treatment records, available baseline and follow-up radiological imaging, and sufficient follow-up information for response assessment. Because all three cohorts were retrospectively assembled and required the availability of adequate tissue, clinical records and follow-up data, selection bias cannot be excluded. In addition, cohort 3 was exploratory and included only patients with documented anti-PD-1 treatment and evaluable radiological follow-up, which may limit the generalizability of the clinical associations. These potential biases may affect the estimated strength of the observed associations but are unlikely to alter the mechanistic conclusions, which were validated using independent datasets, cell models and mouse models.
Ethics oversight	All human studies were approved by the Ethics Committee of the Second Affiliated Hospital of Nanchang University (O-Yi Yan Lun Shen [2025] No. 104), the Ethics Committee of the First Affiliated Hospital of Jinan University (KY-2024-206), and the Ethics Committee of Nanfang Hospital, Southern Medical University (NFEC-202110-K5-01). The studies were conducted in accordance with the Declaration of Helsinki and relevant institutional guidelines. Written informed consent was obtained from all participants, or the requirement for informed consent was waived by the relevant ethics committee for the retrospective use of archived, de-identified specimens and clinical data, as applicable. All animal experiments were approved by the Bioethics Committee of Southern Medical University (IACUC-LAC-20210925-002) and were performed in accordance with the approved institutional guidelines and relevant regulations.

Note that full information on the approval of the study protocol must also be provided in the manuscript.

Field-specific reporting

Please select the one below that is the best fit for your research. If you are not sure, read the appropriate sections before making your selection.

☒ Life sciences ☐ Behavioural & social sciences ☐ Ecological, evolutionary & environmental sciences

For a reference copy of the document with all sections, see nature.com/documents/nr-reporting-summary-flat.pdf

Life sciences study design

All studies must disclose on these points even when the disclosure is negative.

Sample size	No formal statistical power calculation was performed to predetermine sample size. For animal experiments, group sizes were selected based on prior experience with orthotopic HCC and DEN/CCl_4-induced HCC models and were considered sufficient to detect biologically meaningful differences while minimizing animal use. For in vitro experiments, at least three independent biological replicates were included unless otherwise indicated. Human cohort sizes were determined by the number of eligible samples available that met the predefined inclusion criteria, and all qualifying samples were included. Sample sizes for public datasets were determined by the original studies and data availability. Exact sample sizes are provided in the corresponding figure legends and Supplementary Tables.
Data exclusions	For animal experiments, mice were excluded before randomization if tumor implantation failed or if procedure-related death occurred before treatment initiation. No animals were excluded after randomization unless predefined humane endpoint criteria were met. For clinical cohorts and in vitro experiments, no data were excluded from the analysis unless samples failed prespecified quality-control criteria or lacked required clinical or experimental information.
Replication	Details of experimental replicates are given in the figures or figure legends. Within each experimental group the reproducibility was successful, although a degree of variability was detected due to inter-individual diversity.
Randomization	All allocations were random in this study.
Blinding	All data collection and analysis was blinded.

Reporting for specific materials, systems and methods

We require information from authors about some types of materials, experimental systems and methods used in many studies. Here, indicate whether each material, system or method listed is relevant to your study. If you are not sure if a list item applies to your research, read the appropriate section before selecting a response.

Materials & experimental systems

n/a	Involved in the study	
☐	☒	Antibodies
☐	☒	Eukaryotic cell lines
☒	☐	Palaeontology and archaeology
☐	☒	Animals and other organisms
☐	☒	Clinical data
☒	☐	Dual use research of concern
☒	☐	Plants

Methods

n/a	Involved in the study	
☒	☐	ChIP-seq
☐	☒	Flow cytometry
☒	☐	MRI-based neuroimaging

Antibodies

Antibodies used	For western blot:PPARα (Abcam, ab126285, 1:1000), PPARγ (Abcam, ab45036, 1:1000), PPARδ (Abcam, ab23673, 1:1000), PDK4 (Proteintech, 12949-1-AP, 1:1000), GSDMA (Cell Signaling Technology, 49307, 1:1000), GSDMC (Cell Signaling Technology, 4147S, 1:1000), GSDMD (Cell Signaling Technology, 39754, 1:1000), GSDME (Cell Signaling Technology, 19453, 1:1000), cleaved caspase-3 (Cell Signaling Technology, 9661, 1:1000), PARP1 (Cell Signaling Technology, 9532, 1:1000), phospho-PDH E1α (Proteintech, 29582-1-AP, 1:1000), PDH E1α (Proteintech, 18068-1-AP, 1:1000), SP1 (Cell Signaling Technology, 9389, 1:1000), Flag tag (Sigma, F1804, 1:1000), GAPDH (Cell Signaling Technology, 2118, 1:1000), β-actin (Cell Signaling Technology, 4970, 1:1000), α-tubulin (Cell Signaling Technology, 3873, 1:1000), and LAMB1 (Proteintech, 23498-1-AP, 1:1000). HRP-conjugated anti-rabbit IgG (Cell Signaling Technology, 7074, 1:2000) and HRP-conjugated anti-mouse IgG (Cell Signaling Technology, 14709, 1:2000) were used as secondary antibodies.For IHC:PPARα (Abcam, ab126285, 1:200), PDK4 (Proteintech, 12949-1-AP, 1:100), HMGB1 (Abcam, ab18256, 1:200), Calreticulin (Abcam, ab92516, 1:200), and Ki67 (Abcam, ab16667, 1:200).For immunofluorescence and multiplex immunofluorescence:PPARα (Abcam, ab126285, 1:200), CD8 (Abcam, ab237709, 1:100), CD11c (Cell Signaling Technology, 97585, 1:200), α-SMA (Abcam, ab242395, 1:500), F4/80 (Abcam, ab300421, 1:200), CD68 (Abcam, ab283654, 1:100), iNOS (Abcam, ab178945, 1:100), and N-GSDME (Abcam, ab256122, 1:100).For ChIP-qPCR:SP1 (Cell Signaling Technology, 9389, 1:100).For flow cytometry:TruStain FcX PLUS anti-mouse CD16/32 antibody (BioLegend, 156603, clone S17011E; 1 μl per 1 × 10^6 cells in 100 μl), CD45-PerCP/Cy5.5 (BioLegend, 103132, clone 30-F11; 1 μl per 1 × 10^6 cells in 100 μl), CD4-FITC (BioLegend, 100406, clone GK1.5; 2 μl per 1 × 10^6 cells in 100 μl), CD8a-PE/Cy7 (BioLegend, 162312, clone S18018E; 1 μl per 1 × 10^6 cells in 100 μl), CD11b-BV510 (BD Pharmingen, 562950; 2 μl per 1 × 10^6 cells in 100 μl), CD11c-BV605 (BioLegend, 117334, clone N418; 2 μl per 1 × 10^6 cells in 100 μl), NK1.1-APC (BioLegend, 156506,

clone S17016D; 2 μl per 1 × 10⁶ cells in 100 μl), F4/80-BV421 (BioLegend, 123137, clone BM8; 1 μl per 1 × 10⁶ cells in 100 μl), I-A/I-E-BV785 (BioLegend, 107645, clone M5/114.15.2; 1 μl per 1 × 10⁶ cells in 100 μl), Gr-1-PE (BD Pharmingen, clone RB6-8C5, 553128; 1 μl per 1 × 10⁶ cells in 100 μl), CD80-APC (BioLegend, 104714, clone 16-10A1; 1 μl per 1 × 10⁶ cells in 100 μl), CD86-BV421 (BioLegend, 105123, clone PO3; 2 μl per 1 × 10⁶ cells in 100 μl), IFN-γ-Alexa Fluor 700 (BioLegend, 505823, clone XMG1.2; 2 μl per 1 × 10⁶ cells in 100 μl), and Granzyme B-BV421 (BioLegend, 396414, clone QA18A28; 2 μl per 1 × 10⁶ cells in 100 μl). For in vivo treatment and immune-cell depletion:anti-PD-1 mAb (Bio X Cell, BE0146, clone RMP1-14, 100 μg/mouse, i.p., every 3 days), anti-CSF1R antibody (Bio X Cell, BE0213, clone AFS98, 300 μg/mouse, i.p., every 3 days), anti-CD8α antibody (Bio X Cell, BE0061, clone 2.43, 200 μg/mouse, i.p., every 3 days), anti-CD4 antibody (Bio X Cell, BE0003-1, clone GK1.5, 200 μg/mouse, i.p., every 3 days), anti-NK1.1 antibody (Bio X Cell, BE0036, clone PK136, 200 μg/mouse, i.p., every 3 days), anti-Ly6G antibody (Bio X Cell, BE0075-1, clone 1A8, 200 μg/mouse, i.p., every 3 days), anti-CD20 antibody (Bio X Cell, BE0356, clone MB20-11, 250 μg/mouse, i.p., every 5 days), and anti-CD11c antibody (Bio X Cell, BE0038, clone N418, 200 μg/mouse, i.p., every 3 days).

Validation

PPARα, Abcam ab126285
https://www.abcam.com/en-us/products/primary-antibodies/ppar-alpha-antibody-ab126285
PPARγ, Abcam ab45036
https://www.abcam.com/en-us/products/primary-antibodies/ppar-gamma-antibody-ab45036
PPARδ, Abcam ab23673
https://www.abcam.com/en-us/products/primary-antibodies/ppar-delta-antibody-ab23673
PDK4, Proteintech 12949-1-AP
https://www.ptglab.com/products/PDK4-Antibody-12949-1-AP.htm
HMGB1, Abcam ab18256
https://www.abcam.com/en-us/products/primary-antibodies/hmgb1-antibody-ab18256
Calreticulin, Abcam ab92516
https://www.abcam.com/en-us/products/primary-antibodies/calreticulin-antibody-epr3924-er-marker-ab92516
GSDMA, CST 49307
https://www.cellsignal.com/products/primary-antibodies/gasdermin-a-antibody/49307
GSDMC, CST 61921
https://www.cellsignal.com/products/primary-antibodies/gasdermin-c-f9r3m-rabbit-monoclonal-antibody/61921
GSDMD, CST 39754
https://www.cellsignal.com/products/primary-antibodies/gasdermin-d-e9s1x-rabbit-monoclonal-antibody/39754
GSDME, CST 19453
https://www.cellsignal.com/products/primary-antibodies/gasdermin-e-e2x7e-rabbit-monoclonal-antibody/19453
Cleaved caspase-3, CST 9661
https://www.cellsignal.com/products/primary-antibodies/cleaved-caspase-3-asp175-antibody/9661
PARP1, CST 9532
https://www.cellsignal.com/products/primary-antibodies/parp-46d11-rabbit-monoclonal-antibody/9532
Phospho-PDH E1α, Proteintech 29582-1-AP
https://www.ptglab.com/products/Phospho-PDH-E1-Alpha-Ser232-Antibody-29582-1-AP.htm
PDH E1α, Proteintech 18068-1-AP
https://www.ptglab.com/products/PDHA1-Antibody-18068-1-AP.htm
SP1, CST 9389
https://www.cellsignal.com/products/primary-antibodies/sp1-d4c3-rabbit-monoclonal-antibody/9389
Flag tag, Sigma-Aldrich F1804
https://www.sigmaaldrich.com/US/en/product/sigma/f1804
GAPDH, CST 2118
https://www.cellsignal.com/products/primary-antibodies/gapdh-14c10-rabbit-monoclonal-antibody/2118
β-actin, CST 4970
https://www.cellsignal.com/products/primary-antibodies/beta-actin-13e5-rabbit-monoclonal-antibody/4970
α-tubulin, CST 3873
https://www.cellsignal.com/products/primary-antibodies/alpha-tubulin-dm1a-mouse-monoclonal-antibody/3873
Lamin B1/LAMB1, Proteintech 23498-1-AP
https://www.ptglab.com/products/LAMB1-Antibody-23498-1-AP.htm
Ki67, Abcam ab16667
https://www.abcam.com/en-us/products/primary-antibodies/ki67-antibody-sp6-ab16667
CD8α, Abcam ab237709
https://www.abcam.com/en-us/products/primary-antibodies/cd8-alpha-antibody-cal66-ab237709
CD11c, CST 97585
https://www.cellsignal.com/products/primary-antibodies/cd11c-d1v9y-rabbit-monoclonal-antibody/97585
α-SMA, Abcam ab242395
https://www.abcam.com/en-us/products/primary-antibodies/alpha-smooth-muscle-actin-antibody-sp171-bsa-and-azide-free-ab242395
F4/80, Abcam ab300421
https://www.abcam.com/en-us/products/primary-antibodies/f4-80-antibody-epr26545-166-ab300421
CD68, Abcam ab283654
https://www.abcam.com/en-us/products/primary-antibodies/cd68-antibody-epr23917-164-ab283654
iNOS, Abcam ab178945
https://www.abcam.com/en-us/products/primary-antibodies/inos-antibody-epr16635-ab178945
Cleaved N-terminal GSDME, Abcam ab256122
https://www.abcam.com/en-us/products/primary-antibodies/cleaved-n-terminal-dfna5-gsdme-antibody-epr20866-160-bsa-and-azide-free-ab256122

PPARα ChIP antibody, Thermo Fisher MA1-822
https://www.thermofisher.com/antibody/product/PPAR-alpha-Antibody-clone-3B6-PPAR-Monoclonal/MA1-822
HRP-linked anti-rabbit IgG, CST 7074
https://www.cellsignal.com/products/secondary-antibodies/anti-rabbit-igg-hrp-linked-antibody/7074
HRP-linked anti-mouse IgG, CST 7076
https://www.cellsignal.com/products/secondary-antibodies/anti-mouse-igg-hrp-linked-antibody/7076
HRP-conjugated goat anti-rabbit IgG, Abcam ab205718
https://www.abcam.com/en-us/products/secondary-antibodies/goat-rabbit-igg-h-l-hrp-ab205718
CD16/32 Fc block, BioLegend 156603
https://www.biolegend.com/en-us/products/trustain-fcx-plus-anti-mouse-cd16-32-antibody-17085
CD45-PerCP/Cy5.5, BioLegend 103132
https://www.biolegend.com/en-us/products/percp-cyanine5-5-anti-mouse-cd45-antibody-4264
CD4-FITC, BioLegend 100406
https://www.biolegend.com/en-us/products/fitc-anti-mouse-cd4-antibody-248

CD8a-PE/Cy7, BioLegend 162312
https://www.biolegend.com/en-us/products/pe-cyanine7-anti-mouse-cd8a-antibody-23697
CD11b-BV510, BD 562950
https://www.bdbiosciences.com/en-us/products/reagents/flow-cytometry-reagents/research-reagents/single-color-antibodies-ruo/bv510-rat-anti-cd11b.562950
CD11c-BV605, BioLegend 117334
https://www.biolegend.com/en-us/products/brilliant-violet-605-anti-mouse-cd11c-antibody-7865
NK1.1-APC, BioLegend 156506
https://www.biolegend.com/en-us/products/apc-anti-mouse-nk-11-antibody-19843
F4/80-BV421, BioLegend 123137
https://www.biolegend.com/en-us/products/brilliant-violet-421-anti-mouse-f4-80-antibody-7199
I-A/I-E-BV785, BioLegend 107645
https://www.biolegend.com/en-us/products/brilliant-violet-785-anti-mouse-i-a-i-e-antibody-12087
Gr-1-PE, BD 553128
https://www.bdbiosciences.com/en-us/products/reagents/flow-cytometry-reagents/research-reagents/single-color-antibodies-ruo/pe-rat-anti-mouse-ly-6g-and-ly-6c.553128
CD80-APC, BioLegend 104714
https://www.biolegend.com/en-us/products/apc-anti-mouse-cd80-antibody-2340
CD86-BV421, BioLegend 105123
https://www.biolegend.com/en-us/products/brilliant-violet-421-anti-mouse-cd86-antibody-20728
IFN-γ–Alexa Fluor 700, BioLegend 505823
https://www.biolegend.com/en-us/products/alexa-fluor-700-anti-mouse-ifn-gamma-antibody-12538
Granzyme B-BV421, BioLegend 396414
https://www.biolegend.com/en-us/products/brilliant-violet-421-anti-humanmouse-granzyme-b-recombinant-antibody-17577
LIVE/DEAD viability dye, Invitrogen L34994
https://www.thermofisher.com/order/catalog/product/L34994
Anti-PD-1, Bio X Cell BE0146
https://bioxcell.com/invivomab-anti-mouse-pd-1-cd279-be0146
Anti-CSF1R, Bio X Cell BE0213
https://bioxcell.com/invivomab-anti-mouse-csf1r-cd115-be0213
Anti-CD8α, Bio X Cell BE0061
https://bioxcell.com/invivomab-anti-mouse-cd8-alpha-be0061
Anti-CD4, Bio X Cell BE0003-1
https://bioxcell.com/invivomab-anti-mouse-cd4-be0003-1
Anti-NK1.1, Bio X Cell BE0036
https://bioxcell.com/invivomab-anti-mouse-nk1-1-be0036
Anti-Ly6G, Bio X Cell BE0075-1
https://bioxcell.com/invivomab-anti-mouse-ly6g-be0075-1
Anti-CD20, Bio X Cell BE0356
https://bioxcell.com/invivomab-anti-mouse-cd20-be0356
Anti-CD11c, Bio X Cell BE0038
https://bioxcell.com/invivomab-anti-mouse-cd11c-be0038

Eukaryotic cell lines

Policy information about cell lines and Sex and Gender in Research

Cell line source(s)	The human liver cancer cell lines HepG2 (catalog no. SCSP-510; male donor) and PLC/PRF/5 (catalog no. SCSP-5095; male donor) were obtained from the Cell Bank of the Chinese Academy of Sciences (Shanghai, China). The mouse HCC cell line Hepa1-6 (catalog no. SCSP-512; C57L/J background) was obtained from the Cell Bank of the Chinese Academy of Sciences. Hep53.4 cells (catalog no. SAc0678; C57BL/6J background; male) were obtained from Warner Bio (China). HEK293T cells (catalog no. SCSP-502; female donor) used for lentiviral packaging were obtained from the Cell Bank of the Chinese Academy of Sciences. Cells were cultured in Dulbecco's modified Eagle medium supplemented with 10% fetal bovine serum at 37 °C in a humidified incubator containing 5% CO_2. The cell lines have been authenticated by short tandem repeats (STRs) DNA

	profiling. All cells were tested for mycoplasma contamination before use with the Universal Mycoplasma Detection Kit (LT07-218-Lonza) and were not contaminated by mycoplasma.
Authentication	Cell lines were authenticated.
Mycoplasma contamination	All cell lines tested negative for mycoplasma contamination.
Commonly misidentified lines (See ICLAC register)	No commonly misidentified cell lines were used in the study.

Animals and other research organisms

Policy information about studies involving animals; ARRIVE guidelines recommended for reporting animal research, and Sex and Gender in Research

Laboratory animals	Male C57BL/6J mice (Mus musculus; 6–8 weeks old; C57BL/6J background) and male BALB/c nude mice (Mus musculus; 4–6 weeks old; BALB/c-Foxn1nu/nu background) were obtained from Beijing HFK Bioscience Co., Ltd. C57BL/6J mice were used for the orthotopic Hepa1-6/Hep53.4, CCl_4-induced and methionine- and choline-deficient diet-induced models. Male C57BL/6J pups were used for the DEN/CCl_4 model, with DEN administered at postnatal day 14. Ppara−/− mice (C57BL/6JCya-Pparaem1/Cya; strain no. S-KO-16218; C57BL/6JCya background) were obtained from Cyagen Biosciences; male mice aged 6–8 weeks and age- and sex-matched wild-type controls were used. Only male mice were used to maintain consistency across the established HCC models used in this study. Sex was therefore not included as an experimental variable, and no sex-stratified analyses were performed.
Wild animals	No wild animals involved in this study.
Reporting on sex	Male mice were used in the animal experiments to minimize the potential influence of sex-related hormonal variation on inflammation-associated hepatocarcinogenesis and to maintain consistency with commonly used DEN/CCl_4- and orthotopic HCC-model protocols. Sex was therefore not included as an experimental variable. The use of male mice is stated in the Methods and corresponding figure legends where applicable.
Field-collected samples	No field-collected animal samples were used in this study. All animal experiments were performed using laboratory mice maintained under controlled animal-facility conditions.
Ethics oversight	All animal experiments were approved by the Bioethics Committee of Southern Medical University and were performed in accordance with institutional guidelines and applicable animal welfare regulations. Mice were monitored during treatment and were sacrificed at the indicated experimental endpoints or when predefined humane endpoint criteria were met.

Note that full information on the approval of the study protocol must also be provided in the manuscript.

Clinical data

Policy information about clinical studies

All manuscripts should comply with the ICMJE guidelines for publication of clinical research and a completed CONSORT checklist must be included with all submissions.

Clinical trial registration	*Provide the trial registration number from ClinicalTrials.gov or an equivalent agency.*
Study protocol	*Note where the full trial protocol can be accessed OR if not available, explain why.*
Data collection	*Describe the settings and locales of data collection, noting the time periods of recruitment and data collection.*
Outcomes	*Describe how you pre-defined primary and secondary outcome measures and how you assessed these measures.*

Plants

Seed stocks	*Report on the source of all seed stocks or other plant material used. If applicable, state the seed stock centre and catalogue number. If plant specimens were collected from the field, describe the collection location, date and sampling procedures.*
Novel plant genotypes	*Describe the methods by which all novel plant genotypes were produced. This includes those generated by transgenic approaches, gene editing, chemical/radiation-based mutagenesis and hybridization. For transgenic lines, describe the transformation method, the number of independent lines analyzed and the generation upon which experiments were performed. For gene-edited lines, describe the editor used, the endogenous sequence targeted for editing, the targeting guide RNA sequence (if applicable) and how the editor was applied.*
Authentication	*Describe any authentication procedures for each seed stock used or novel genotype generated. Describe any experiments used to assess the effect of a mutation and, where applicable, how potential secondary effects (e.g. second site T-DNA insertions, mosiacism, off-target gene editing) were examined.*

Flow Cytometry

Plots

Confirm that:

☒ The axis labels state the marker and fluorochrome used (e.g. CD4-FITC).

☒ The axis scales are clearly visible. Include numbers along axes only for bottom left plot of group (a 'group' is an analysis of identical markers).

☒ All plots are contour plots with outliers or pseudocolor plots.

☒ A numerical value for number of cells or percentage (with statistics) is provided.

Methodology

Sample preparation	Fresh orthotopic HCC tissues were carefully dissected from the liver, and necrotic areas, visible blood clots and surrounding non-tumor tissues were removed. Tumors were washed with cold PBS, mechanically minced into small fragments, and digested in RPMI-1640 medium containing 2% fetal bovine serum, 1 mg/mL collagenase IV and 200 µg/mL DNase I at 37°C for 30 min with gentle rotation. The digested cell suspension was passed through a 70 µm cell strainer to remove undigested tissue fragments and debris. Cells were centrifuged, resuspended in 30% Percoll and layered onto 70% Percoll. After density-gradient centrifugation, mononuclear cells at the interface were collected, washed and subjected to red blood cell lysis when necessary. Cell number and viability were determined before staining.For flow-cytometry staining, cells were stained with LIVE/DEAD™ viability dye and blocked with TruStain FcX PLUS anti-mouse CD16/32 antibody before incubation with fluorochrome-conjugated antibodies against lineage and activation markers. For intracellular cytokine staining of CD8⁺ T cells, cells were stimulated for 4 h with cell stimulation cocktail plus protein transport inhibitors, followed by fixation/ permeabilization and staining for IFNγ and Granzyme B.
Instrument	Flow-cytometry data were acquired using a BD Fortessa flow cytometer (BD Biosciences, San Jose, CA, USA).
Software	Flow-cytometry data were analyzed using FlowJo 10.8.2 software.
Cell population abundance	Cell population abundance was calculated as the percentage of cells within the indicated parent population. Dead cells were excluded using LIVE/DEAD™ viability staining, and doublets were excluded based on FSC-A/FSC-H and SSC-A/SSC-H parameters. Tumor-infiltrating immune cells were first gated from viable singlets, followed by CD45⁺ immune-cell gating. CD8⁺ T cells, CD4⁺ T cells, dendritic cells, macrophages, NK cells and myeloid populations were quantified within the CD45⁺ population using the indicated lineage markers. CD45⁻ tumor cells were used for analyses of tumor-cell death where applicable. Frequencies of IFNγ⁺ and Granzyme B⁺ CD8⁺ T cells were calculated within the CD8⁺ T-cell gate. DC maturation was assessed by the proportion of CD80⁺ or CD86⁺ cells within the dendritic-cell gate.
Gating strategy	Representative gating strategies are shown in the corresponding figures. Briefly, debris was excluded using FSC/SSC parameters, doublets were excluded using FSC-A/FSC-H and SSC-A/SSC-H gating, and dead cells were excluded using LIVE/ DEAD™ staining. Viable singlets were then gated into CD45⁺ immune cells and CD45⁻ tumor-cell fractions. Immune-cell subsets were identified using the following markers: CD4⁺ T cells, CD8a⁺ T cells, CD11c⁺MHC-II⁺ dendritic cells, F4/80⁺ macrophages, NK1.1⁺ NK cells and Gr-1⁺ myeloid cells. CD8⁺ T-cell function was assessed by intracellular IFNγ and Granzyme B staining after stimulation. The same gating strategy was applied to all samples within each experiment, and gating was performed blinded to group allocation whenever feasible.

☒ Tick this box to confirm that a figure exemplifying the gating strategy is provided in the Supplementary Information.
