# Supplementary material for: PPARα activation overcomes fibroinflammatory liver microenvironment-associated anti-PD-1 resistance in hepatocellular carcinoma by mediating GSDME-dependent pyroptosis
Source: Nat Commun. 2026 Aug 6;17:9474. doi: 10.1038/s41467-026-75770-7 (PMC13542117; doi:10.1038/s41467-026-75770-7)
Supplement: Supplementary file 3 [file 41467_2026_75770_MOESM3_ESM.pdf]

nature portfolio

Peer Review File

PPARα activation overcomes fibroinflammatory liver microenvironment-associated anti-PD-1 resistance in hepatocellular carcinoma by mediating GSDME-dependent pyroptosis

Corresponding Author: Dr Yuchuan Jiang

This file contains all reviewer reports in order by version, followed by all author rebuttals in order by version.

A version of this paper was originally rejected for publication by Nature Communications, however that decision was reconsidered after appeal by the authors.

Version 0:

Reviewer comments:

Reviewer #1

(Remarks to the Author)
This study investigates a fibroinflammatory liver microenvironment (FILM) common in hepatocellular carcinoma and connects it to defective immunogenic cell death and resistance to anti–PD-1 therapy. The authors delineate a FILM–NOS2–PPARα axis: cancer-associated fibroblasts recruit and polarize macrophages toward a $NOS2^+$ phenotype; macrophage-derived nitric oxide represses tumor-cell PPARα, which lowers PDK4, dampens mitochondrial ROS and caspase-3 activation, and thereby blocks GSDME-dependent pyroptosis. Pharmacologic activation of PPARα restores pyroptosis, enhances dendritic-cell and $CD8^+$ T-cell function, and sensitizes tumors to PD-1 blockade in mice, with fenofibrate providing synergy and retrospective clinical support. Conceptually, the work links stromal inflammation, nuclear-receptor signaling, metabolism, and cell-death biology to motivate a translational strategy centered on PPARα activation.
Overall, this work innovatively proposes that activation of PPARα promotes GSDME-mediated pyroptosis; moreover, it advances a clinically meaningful combination strategy using PPARα agonists to overcome FILM-induced resistance to immunotherapy. However, several issues remain to be addressed.
Major
1.Many prior reports indicate that PPARα enhances metabolic homeostasis and suppresses cell death (including apoptosis, pyroptosis, and ferroptosis), whereas here PPARα activation is concluded to promote GSDME-mediated pyroptosis in HCC. Please reconcile this with the literature and define the HCC-specific context (e.g., FAO dependency, redox state, or transcriptional co-regulators) that explains the divergent outcome.
2.Fig. 1F–G relies on GSDME activation, yet GSDMD is widely considered the principal executioner of pyroptosis. Please justify prioritizing GSDME over GSDMD. If cleavage was observed only for GSDME but not for GSDMD, what mechanism drives selective GSDME activation in this setting?
3.In Fig. 1E–F, reduced TUNEL signal and GSEA are used to infer suppression of pyroptosis by FILM. Because TUNEL positivity can also reflect apoptosis or ferroptosis, please clarify whether FILM concurrently suppresses other death modalities and provide discriminating evidence.
4.Fig. 1I–J shows that GSDME overexpression enhances immunotherapy efficacy in the FILM model; however, enforced GSDME is inherently expected to increase pyroptosis and immunogenicity. These groups alone do not establish that FILM-mediated suppression of pyroptosis causes resistance. Please include a negative-control arm in a non-FILM model to demonstrate FILM-specific causality.
5.Fig. 2A identifies multiple pathways differing between FILM and non-FILM tumors. Beyond PPARα, if other pathways contribute to treatment resistance? Moreover, if FILM suppresses GSDME-mediated pyroptosis, why are pyroptosis/GSDME signatures not among the enriched pathways? Please clarify this apparent inconsistency or provide additional analyses.
6.Figure 3A–C show that PPARα overexpression or knockout does not significantly affect GSDME cleavage, whereas treatment with PPARα agonists effectively induces GSDME cleavage, indicating a ligand-dependent regulation of GSDME by PPARα. In the patient samples and mouse in vivo models studied here, what are the operative ligands of PPARα?
7.Fig. 5A uses clodronate liposomes to deplete macrophages. For a more selective assessment, please add an anti-

CSF1R–based depletion strategy with appropriate controls and compare the two approaches.
8.Fig. 5I profiles M1-associated genes in FILM-CAF–recruited macrophages. Please expand phenotyping to include M2 markers and other polarization signatures. Given that Il1b and Tnfa are also induced alongside Nos2, the potential synergy between these cytokines and NOS2-derived NO merits further investigation.
9.NOS2⁺ macrophages can arise under non-FILM stimuli (e.g., TLR ligation). Do NOS2⁺ macrophages increase in abundance specifically within FILM? Please quantify NOS2⁺ macrophages in both FILM patients and mouse models to establish their prevalence and link to disease course.
10.Fig. 5L implicates NO in repressing PPARα expression, but the transcriptional mechanism is not defined. Please delineate the upstream factors and promoter/chromatin events by which NO down-regulates PPARA (e.g., involvement of NF-κB/FOXO/HDACs, S-nitrosylation effects, or enhancer accessibility).
Minor
1.Fig. 2A legend: five NES dot sizes are shown but only four numerical values are provided; please correct the legend.
2.Fig. 4A labeling: the "GW9578" designation is inconsistent with the main text and caption; please standardize the nomenclature.
3.Fig. 5A vs. Fig. 5B: FILM-derived tumor-infiltrating lymphocytes appear to reduce tumor-cell PPARα, yet lymphocyte depletion shows no effect. Please clarify this discrepancy, including timing, subsets targeted, and experimental conditions.

Reviewer #2

(Remarks to the Author)
The authors of this study try to demonstrate that the fibro-inflammatory liver microenvironment (FILM) fosters NOS2+ macrophages, which suppress PPARα in tumor cells, down-regulating the PDK4–ROS–caspase-3 axis and blocking GSDME-mediated pyroptosis, thereby conferring anti–PD-1 resistance. Ligand activation of PPARα with fenofibrate restores pyroptosis, ignites CD8+ T-cell immunity, and synergizes with anti–PD-1 therapy in multiple murine models. In principle this study would be a good contribution to the field, however, more data and interpretation are needed to support their conclusions and improve the readability of this manuscript.
Major comments:
1. For Figure 1F, could the complete GSEA results be displayed? Is the pyroptosis pathway among the most or one of the most significantly altered pathways?
2. In Figure 1G, the more prominent GSDME cleavage in Non-FILM tumors seems to result from higher total GSDME protein expression; tumors with low total GSDME also show correspondingly low GSDME-N levels.
Inflammatory conditions may activate STAT3 and other pathways to up-regulate GSDME expression, whose abundance is a prerequisite for tumor-cell pyroptosis; therefore, the authors should compare total GSDME expression levels between Non-FILM and FILM environments.
3. In Figure 2A, the PPAR pathway is not among the most prominently altered pathways. Why did the authors choose to pursue this pathway for subsequent investigation?
4. Hepa 1-6 is not an ideal cell line for studying HCC tumor immunology. Hepa1-6 was derived from a C57L mice, which has minor MHC mismatch with C57BL/6 mice. Therefore, at least one additional model—such as the RIL-175 cell line—should be used to validate the conclusions presented in this study.
5. The manuscript first establishes that FILM lowers PPARα expression and that this reduction cripples GSDME-mediated pyroptosis and immunotherapy response. Logically, the next question should be how FILM down-regulates PPARα; instead, the authors pivot to detailing how PPARα activation in Non-FILM settings induces pyroptosis, relegating the mechanism of FILM-driven PPARα suppression to a short section at the end. This disrupts narrative flow and weakens overall coherence.
6. The middle section of this manuscript demonstrates that activation, rather than expression level, of PPARα is the decisive trigger for downstream GSDME-pyroptosis, which appears to contradict the earlier claim that FILM suppresses PPARα expression to suppress pyroptosis and promote anti-PD-1 resistance. Is FILM linked to PPARα activation, and if so, how? Clarifying this interplay is essential for coherence.
7. FILM lowers PPARα expression and thereby causes anti-PD-1 resistance, and restoring PPARα expression can reverse this resistance. Translational logic would therefore favor agents that re-induce PPARα expression. However, because the authors emphasize that activation—rather than absolute expression level—of PPARα is the critical determinant for inducing tumor-cell pyroptosis, they opt to use PPARα agonists to enhance immunotherapeutic efficacy. And they also predict benefit only in patients whose tumors already express high PPARα. This creates a paradox: if FILM has already suppressed PPARα, how could activation of the residual low-level of PPARa be sufficient to active GSDME-pyroptosis?
8. Although not explicitly stated, the authors' data imply that tumor-cell-intrinsic PPARα is the pivotal switch for therapy-induced apoptosis and pyroptosis, whereas it is clear that PD-1 blockade drives tumor-cell death largely through other immune effector mechanisms.
Minor comments:
9. For the right-hand panel of Figure 1G, two tumors per group are insufficient to represent the cohort; results from at least five tumors per group should be presented and statistically analyzed.
10. The n number of sample size should be stated in the corresponding figure legends.

Reviewer #3

(Remarks to the Author)
This manuscript investigates the role of PPARα activation in promoting GSDME-mediated pyroptosis and enhancing anti–PD-1 responsiveness in hepatocellular carcinoma. The topic is conceptually attractive, and the proposed link between metabolic signaling and immunogenic cell death has potential translational significance. However, despite intriguing

preliminary findings, the study in its current form lacks the mechanistic depth and experimental transparency required to support its major claims. The data provide correlations rather than causality, and critical methodological information is missing, which limits reproducibility and interpretability.

Major Concerns

1. The central conceptual gap lies in the unproven necessity and mechanistic positioning of GSDME within the PPARα–PD-1 pathway. While the authors propose that PPARα activation induces ROS/caspase-3–dependent GSDME cleavage to enhance anti–PD-1 efficacy, there is no direct evidence demonstrating that GSDME is required rather than a byproduct of metabolic stress. A comprehensive scRNA-seq combined with CITE-seq analysis comparing Gsdme-KO and WT tumor-bearing mice under PPARα agonist treatment is essential to delineate how GSDME loss alters immune-cell composition, activation, and transcriptional networks (e.g., macrophage polarization, dendritic-cell activation, T-cell exhaustion).
This analysis would also reveal the cell-type preference of PPARα activation and the cellular susceptibility to GSDME deletion, providing insight into whether pyroptosis selectively affects particular immune or tumor populations. In addition, UMAP plots visualizing Gsdme expression and KO-dependent clustering shifts should be presented to confirm both the efficiency and cell-type specificity of GSDME deletion within the tumor microenvironment. Please also ensure open access to the raw scRNA-seq and CITE-seq datasets.

2. The manuscript lacks critical details regarding mouse strains, sex, age, and genotyping of Gsdme-KO mice, as well as sources, catalog numbers, and doses of anti–PD-1 antibodies, GW7647, fenofibrate, and viral vectors (LV, AAV). These omissions preclude evaluation of reproducibility and raise concerns about data reliability. All key materials and their validation (e.g., knockout confirmation, vector construction maps, titers) should be explicitly stated.

3. This work lacks raw data and data-sharing transparency. No accession numbers are provided for RNA-seq or other omics datasets. Raw Western blot images, original flow-cytometry files, and unprocessed microscopy data are not included. Public deposition of sequencing data (GEO/ArrayExpress) and provision of uncropped images are mandatory for reproducibility.

4. The linear signaling cascade is overstated. Current evidence shows correlation but not causation between PPARα activation, PDK4 induction, ROS accumulation, and GSDME cleavage. Rigorous loss- and gain-of-function validation (PDK4 knockout, kinase-dead rescue, PDH activity, mitochondrial respiration, and metabolic-flux assays) is required to establish PDK4 as a mechanistic intermediary.

5. ROS is interpreted as an upstream trigger of caspase-3 activation, yet PPARα primarily regulates transcriptional programs rather than acute oxidative stress. Without time-course or mitochondrial functional data, the claim that ROS precedes caspase-3 activation is speculative. Specific genetic and temporal assays should be used to define this hierarchy.

6. The identification of Annexin V^+/7-AAD^+/$CD45^-$ cells as pyroptotic is not justified, as this phenotype typically marks late apoptosis. Definitive evidence—such as co-localization of cleaved GSDME, IL-1β/IL-18 release, or real-time membrane-integrity assays—is required to confirm true pyroptosis.

7. The identification of $NOS2^+$ macrophages as the major immunosuppressive population relies on immunostaining and limited in-vitro assays. Without single-cell profiling or multiplex spatial validation, it remains unclear whether $NOS2^+$ cells form a distinct macrophage subset or a transient activation state. Moreover, the mechanism by which NO suppresses PPARα (direct S-nitrosylation vs. secondary cytokine effects) is not experimentally addressed.

8. Because PPARα agonists affect multiple cell types, it is unclear whether the observed pyroptosis and PD-1 sensitization originate from tumor-intrinsic or immune-cell–intrinsic PPARα activity. Tumor-specific versus host-specific knockout or bone-marrow transplantation experiments are required to delineate cell-type specificity.

9. Randomization, blinding, and sample-size justification are not described. Several figures display only mean ± SD without individual data points, hindering evaluation of variance and significance. Statistical parameters and exact n values should be clearly reported.

Minor Points
1. Inadequate citation support in the Introduction. Key statements rely on review articles rather than primary mechanistic studies (e.g., Refs 17–18 and 22–23). Original research citations should replace reviews to substantiate claims regarding GSDME's role in immunotherapy and expression in HCC.
2. Typographical and nomenclature errors. For example, "PAPRγ" should read "PPARγ." Check all figures and legends carefully for consistency.
3. Missing methodological details. Include antibody sources and dilution, RNA-seq analysis pipeline, normalization method, and statistical tests used for gene-expression comparisons.
4. Data presentation. Provide individual datapoints in bar graphs, specify number of biological replicates, and show representative uncropped Western blots.
5. Clinical cohort limitations. The retrospective analysis (14 vs 25 patients) is underpowered; authors should tone down translational claims and describe inclusion criteria and potential confounders.
6. Drug selectivity. Discuss pharmacological differences between GW7647 and fenofibrate and clarify whether both act via identical mechanisms.

Reviewer #4

(Remarks to the Author)
Peng Chen et al. describe an interesting study of the mechanisms underlying the fibroinflammatory liver microenvironment (FLIM)-driven immunosuppression in hepatocellular carcinoma. They provide evidence that NOS2+ macrophages suppress PARP expression/activation, which lowers PKD4 levels, leading to decreased ROS levels, further suppressing caspase-3/GSDME-mediated pyroptosis. This is a well-designed study using omics analyses of available clinical samples as well as the validation of the mechanisms in multiple preclinical mouse models. By retrospective analysis of clinical cohorts and utilizing preclinical FLIM-HCC models, the authors demonstrate that PARP activator fenofibrate improves anti-PD1 therapy. This is a timely study, demonstrating an interesting mechanism by which even an inflammatory environment can lead to suppression of immunogenic cell death. I only have some minor comments.
1. Fig 1G: In human samples, it seems that full-length GSDME as well as GSDMA (Fig. S2C) expression is higher in non-FLIM samples 1-3, which then directly correlates with increased levels of the NT fragment of GSDME. The author should analyze the effect of FLIM on FL GSDME expression and the effect of anti-PD1 therapy on it as well. The effect of anti-PD1 therapy in the mouse model is more telling, although one cannot miss that anti-PD-1 treatment boosts FL GSDME levels.
2. A surprising result is relatively low cytotoxicity due to exogenously provided NT-GSDME. Is FLIM also suppressing NT-GSDME pore-forming activity? This experiment, however, requires a bit more detailed technical description. It is unclear how the transduced cell line was prepared. Introduction of constitutively expressed NT-GSDME would lead to immediate cell death. Please provide a detailed description in the methods (plasmid preparation, cell line transduction and animal model).
3. Fig. 5A: The labelling GW7647/Fenofibrate is misleading. Figs. 5F and 5O: The authors should provide quantification of cleaved GSDME levels, if possible, also by western blot. Fig. 5I: It seems that fenofibrate worsens the effect of antiPD1 therapy, or are the curves mislabeled?
4. If the ROS are behind the activation of caspase-3/GSDME pyroptosis, could ROS-inducing drugs provide the same effect (skipping PARP /PKD4 steps)?
5. Perhaps the steps downstream of PKD4 could be elaborated a bit more, at least in the discussion? How does ROS induce activation of caspase-3? Is this caspase-9 initiated cascade?

Version 1:

Reviewer comments:

Reviewer #1

(Remarks to the Author)
The authors have addressed all of my concerns, and the manuscript is now suitable for publication.

Reviewer #2

(Remarks to the Author)
The author's revisions have addressed most of the issues I raised, the overall quality of the revised manuscript has improved significantly, and I recommend acceptance for publication.

Reviewer #3

(Remarks to the Author)
The authors did a great job. The revision is thorough. The paper is much stronger now. I have a few remaining suggestions about wording and figure placement.

1. The data support immunogenic cell death, but total GSDME levels don't change—only its cleavage does. More importantly, GSDME is highly expressed in normal tissues and often plays protective roles there (see PMIDs: 41638192, 41407670, 40707439, 41701674). This means "abundance" does not equal "activation." FILM mainly acts on upstream metabolic thresholds (ROS and caspase-3 levels), not on GSDME itself. The authors should present GSDME as a "downstream executor." This neatly explains why giving fenofibrate systemically is safe in normal tissues—they don't have the FILM-induced metabolic stress. The above-mentioned publications should be added into the references list for discussion.

2. The paper focuses on FILM, but the key datasets that define it, especially the $CD45^+$ immune scRNA-seq and spatial transcriptomics, are stuck in the Supplementary Materials. For a journal like Nature Communications, these foundational data are critical for showing the study's impact. The authors should combine these panels and move them to the main figures, so readers can immediately see the multi-omics strength of the study.

3. The PDK4 kinase-dead rescue experiment (proving that kinase activity drives pyroptosis) and the Ppara-/- reciprocal transplants (proving the phenotype is tumor-intrinsic) are the most important mechanistic findings in the paper. But they are still hidden in Figures S16 and S18. These need to be in the main figures. To make room, move the repetitive or purely descriptive phenotypic panels to the supplement.

Reviewer #4

(Remarks to the Author)
The authors addressed all my concerns. I have no further suggestions.

Open Access This Peer Review File is licensed under a Creative Commons Attribution 4.0 International License, which permits use, sharing, adaptation, distribution and reproduction in any medium or format, as long as you give appropriate credit to the original author(s) and the source, provide a link to the Creative Commons license, and indicate if changes were made.
In cases where reviewers are anonymous, credit should be given to 'Anonymous Referee' and the source.
The images or other third party material in this Peer Review File are included in the article's Creative Commons license, unless indicated otherwise in a credit line to the material. If material is not included in the article's Creative Commons license and your intended use is not permitted by statutory regulation or exceeds the permitted use, you will need to obtain permission directly from the copyright holder.
To view a copy of this license, visit https://creativecommons.org/licenses/by/4.0/

Reviewers' comments:

Reviewer #1 (Remarks to the Author):

This study investigates a fibroinflammatory liver microenvironment (FILM) common in hepatocellular carcinoma and connects it to defective immunogenic cell death and resistance to anti–PD-1 therapy. The authors delineate a FILM–NOS2–PPARα axis: cancer-associated fibroblasts recruit and polarize macrophages toward a $NOS2^{+}$ phenotype; macrophage-derived nitric oxide represses tumor-cell PPARα, which lowers PDK4, dampens mitochondrial ROS and caspase-3 activation, and thereby blocks GSDME-dependent pyroptosis. Pharmacologic activation of PPARα restores pyroptosis, enhances dendritic-cell and $CD8^{+}$ T-cell function, and sensitizes tumors to PD-1 blockade in mice, with fenofibrate providing synergy and retrospective clinical support. Conceptually, the work links stromal inflammation, nuclear-receptor signaling, metabolism, and cell-death biology to motivate a translational strategy centered on PPARα activation.

Overall, this work innovatively proposes that activation of PPARα promotes GSDME-mediated pyroptosis; moreover, it advances a clinically meaningful combination strategy using PPARα agonists to overcome FILM-induced resistance to immunotherapy. However, several issues remain to be addressed.

Major
1.Many prior reports indicate that PPARα enhances metabolic homeostasis and suppresses cell death (including apoptosis, pyroptosis, and ferroptosis), whereas here PPARα activation is concluded to promote GSDME-mediated pyroptosis in HCC. Please reconcile this with the literature and define the HCC-specific context (e.g., FAO dependency, redox state, or transcriptional co-regulators) that explains the divergent outcome.

Response:
We sincerely thank the reviewer for raising this important point. PPARα has been widely reported to maintain metabolic homeostasis and, in many non-malignant or metabolic injury settings, to protect against apoptosis, ferroptosis, or inflammatory cell death. This apparent discrepancy highlights the context-dependent nature of PPARα signaling (PMID: 24944896; 35703725; 30020428; 35703725). PPARα is a ligand-activated nuclear receptor whose biological output depends on cell type, ligand availability, substrate utilization, redox-buffering capacity, and the transcriptional targets engaged in a given cellular state (PMID: 16322072; 23802046; 20936127). We have now revised the manuscript to better reconcile our findings with the existing literature and to emphasize the context-dependent nature of PPARα signaling.

In the original submission, we had shown that PPARα expression alone was insufficient to induce pyroptosis in vitro. Instead, ligand-dependent activation by GW7647 was required to induce PPARα nuclear translocation, upregulate FAO-related target genes, promote mitochondrial ROS

accumulation, activate caspase-3, and trigger GSDME cleavage. We further showed that blockade of CPT1-dependent FAO, mitochondrial ROS, or caspase-3 abolished GW7647-induced LDH/HMGB1 release, membrane blebbing, and GSDME cleavage (See revised Figure 4F-H; Figure S14). In addition, our original data identified PDK4 as a direct transcriptional target of ligand-activated PPARα and demonstrated that PDK4 knockdown or kinase-dead PDK4 reconstitution impaired mitochondrial ROS accumulation and GSDME cleavage (See revised Figure 5D; Figure S6).

To further define the HCC-specific context and the cellular source of PPARα responsible for this effect *in vivo*, we have now added reciprocal genetic experiments. Genetic deletion of Pparα in HCC cells markedly impaired the ability of GW7647 to suppress tumor growth and enhance anti-PD-1 efficacy. Conversely, in Pparα−/− recipient mice bearing PPARα-intact HCC tumors, GW7647 retained its antitumor and anti-PD-1–sensitizing effects. These new data support a predominant role of tumor-cell-intrinsic PPARα in this therapeutic response (See revised Figure S18F-K).

Based on these findings, we have revised the Discussion to clarify that we do not propose PPARα as a universally pro-pyroptotic factor. Rather, our data define a specific HCC context in which ligand-activated tumor-cell PPARα transcriptionally upregulates PDK4 and engages a PDK4–FAO–mitochondrial ROS–caspase-3–GSDME axis. When this metabolic-redox program is coupled to intact GSDME expression, PPARα activation converts metabolic rewiring into pyroptotic immunogenic cell death, thereby enhancing anti-PD-1 responsiveness (See revised Main Text page 19 line 18-26).

2.Fig. 1F–G relies on GSDME activation, yet GSDMD is widely considered the principal executioner of pyroptosis. Please justify prioritizing GSDME over GSDMD. If cleavage was observed only for GSDME but not for GSDMD, what mechanism drives selective GSDME activation in this setting?

Response:
We thank the reviewer for raising this important point. We agree that GSDMD is the canonical executioner of inflammasome-driven pyroptosis, particularly downstream of inflammatory caspases such as caspase-1/4/5/11 [PMID: 26375003; 26375259; 26611636; 27383986]. However, pyroptotic membrane rupture can also be executed by other gasdermin family members depending on the upstream protease and cellular context. In particular, GSDME is a well-established substrate of activated caspase-3 and can convert caspase-3 activation from apoptotic cell death into pyroptotic membrane rupture in GSDME-expressing cells [PMID: 28045099; 28459430]. GSDME-mediated

pyroptosis has also been shown to enhance antitumor immunity and suppress tumor growth [PMID: 32188940].

In our study, we prioritized GSDME over GSDMD based on both cleavage pattern and functional evidence. In the original submission, we showed that FILM markedly reduced GSDME cleavage without altering total GSDME expression, whereas cleavage of other examined gasdermins including GSDMD, was not detectably altered. Moreover, anti-PD-1 therapy enhanced GSDME cleavage only in tumors implanted in control livers, but not in FILM tumors. Functionally, GSDME knockout impaired anti-PD-1 efficacy, whereas cleaved GSDME overexpression restored anti-PD-1 responsiveness in FILM tumors, accompanied by enhanced tumor-cell membrane disruption and immune activation. These results indicate that GSDME cleavage is not merely correlative, but functionally required for the immunotherapeutic response in our model (See revised Figure 1F-M; Figure S2E, F; Figure S3).

Mechanistically, our data support selective GSDME activation because the upstream death signal in this setting is caspase-3-dependent rather than canonical inflammasome/GSDMD-dependent. Ligand-activated PPARα induced FAO-linked mitochondrial ROS accumulation, followed by caspase-3 activation and GSDME cleavage. Blocking pan-caspase activity or caspase-3 abolished GW7647-induced LDH/HMGB1 release and membrane blebbing, whereas inhibition of caspase-1 or caspase-11 had little effect (See revised Figure 4F-H). In addition, inhibition of mitochondrial ROS or CPT1-dependent FAO prevented caspase-3 activation, GSDME cleavage, and pyroptotic cell death (See revised Figure 4G, H; Figure S14B-D). These findings indicate that the relevant pyroptotic program in this HCC context is mediated by a PPARα–PDK4/FAO–mitochondrial ROS–caspase-3–GSDME cascade, rather than the canonical inflammasome–GSDMD pathway.

3. In Fig. 1E–F, reduced TUNEL signal and GSEA are used to infer suppression of pyroptosis by FILM. Because TUNEL positivity can also reflect apoptosis or ferroptosis, please clarify whether FILM concurrently suppresses other death modalities and provide discriminating evidence.

Response:
We thank the reviewer for this important comment. We agree that TUNEL positivity is not specific for pyroptosis and may also reflect apoptosis or other forms of regulated cell death. We have therefore revised the manuscript to clarify that reduced TUNEL staining was used only as an initial indication of altered tumor cell death, rather than as direct evidence of pyroptosis suppression.

To address the reviewer's concern in the revised manuscript, we added discriminating analyses of other major cell death modalities. Specifically, we examined apoptosis- and ferroptosis-related markers and found that cleaved PARP1 and ferroptosis-related markers, including GPX4, ACSL4, and SLC7A11, were not significantly decreased in FILM tumors compared with control tumors (See revised Figure S2B, C). Additionally, we also clarified this logic in the Results section, emphasizing that TUNEL staining prompted further investigation but was not used as pyroptosis-specific evidence (See revised Main Text page 7 line 25-27).

In the original submission, we showed that FILM tumors exhibited reduced TUNEL positivity together with suppressed pyroptosis pathway enrichment by GSEA. We also found that FILM selectively reduced GSDME cleavage without markedly affecting total GSDME expression, and that anti-PD-1 therapy enhanced GSDME cleavage only in tumors implanted in control livers but not in FILM tumors. Functionally, GSDME knockout impaired anti-PD-1 efficacy, whereas cleaved GSDME overexpression restored anti-PD-1 responsiveness in FILM tumors, accompanied by increased terminal membrane-disrupted tumor cells and immune activation. These data support a functional requirement for GSDME-mediated pyroptosis in anti-PD-1 responsiveness.

Collectively, these original and newly added data demonstrate that although TUNEL itself is not pyroptosis-specific, FILM preferentially suppresses GSDME-mediated pyroptotic immunogenic cell death rather than broadly suppressing apoptosis or ferroptosis.

4. Fig. 1I–J shows that GSDME overexpression enhances immunotherapy efficacy in the FILM model; however, enforced GSDME is inherently expected to increase pyroptosis and immunogenicity. These groups alone do not establish that FILM-mediated suppression of pyroptosis causes resistance. Please include a negative-control arm in a non-FILM model to demonstrate FILM-specific causality.

Response:

We thank the reviewer for this constructive suggestion. In the original submission, we had shown that FILM tumors displayed reduced endogenous GSDME cleavage and impaired anti-PD-1 responsiveness, and that cleaved GSDME expression restored anti-PD-1 efficacy in FILM tumors. To directly test whether this effect reflects FILM-specific causality rather than nonspecific enhancement by enforced GSDME activation, we have now added a non-FILM/control-liver negative-control arm. In control-liver tumors, where anti-PD-1 monotherapy was already effective, cleaved GSDME overexpression reduced tumor growth but did not further enhance the efficacy of

anti-PD-1 therapy (See revised Figure S3E, F). These data support a causal role for FILM-mediated suppression of GSDME-dependent pyroptosis in anti-PD-1 resistance, rather than a nonspecific consequence of forced GSDME activation. We have revised the Results section accordingly (See revised Main Text page 8 line 14-17).

5. Fig. 2A identifies multiple pathways differing between FILM and non-FILM tumors. Beyond PPARα, if other pathways contribute to treatment resistance? Moreover, if FILM suppresses GSDME-mediated pyroptosis, why are pyroptosis/GSDME signatures not among the enriched pathways? Please clarify this apparent inconsistency or provide additional analyses.

Response:

We thank the reviewer for these important questions regarding pathway selection and enrichment analysis. We agree that FILM is a complex microenvironmental state and that multiple pathways altered between FILM and non-FILM tumors may cooperate to promote anti-PD-1 resistance. Our focus on PPARα was based on experimental prioritization rather than the assumption that other pathways are irrelevant. Specifically, we have revised Figure 2A to display KEGG enrichment results from both human HCC tissues and mouse HCC models, which showed consistent downregulation of PPAR signaling in FILM HCC across species. We then prioritized PPARα because FILM selectively suppressed PPARα expression and pathway activity, but not PPARγ or PPARδ, and because PPARα expression was reduced in anti-PD-1 non-responders. Moreover, single-cell RNA-seq analysis linked higher PPARα expression and pathway activity in HCC cells to higher pyroptosis scores and cytotoxic T-cell infiltration, and functional experiments showed that PPARα restoration promoted GSDME cleavage, immunogenic cell death, and anti-PD-1 responsiveness, whereas GSDME loss abrogated the sensitizing effect of PPARα. Thus, PPARα was selected as the functionally validated upstream regulator connecting FILM to suppressed pyroptotic immunogenicity and anti-PD-1 resistance, while we acknowledge that additional FILM-altered pathways may also contribute and warrant further investigation.

Regarding the apparent absence of pyroptosis/GSDME signatures among the enriched pathways in Figure 2A, this reflects both database-specific pathway annotation and the post-translational nature of GSDME activation. Figure 2A was based on KEGG pathway enrichment, which is better suited for canonical signaling and metabolic networks but does not specifically resolve the caspase-3–GSDME pyroptotic execution axis examined in this study. In contrast, Gene Ontology (GO) focuses on biological processes with higher functional granularity and includes pyroptosis-related gene sets. Accordingly, the GO-based gene-set analysis in Figure S2 directly assessed pyroptosis-related activity and showed its consistent suppression in FILM tumors across both human and mouse

datasets. Importantly, FILM did not markedly reduce total GSDME expression but selectively suppressed GSDME cleavage, indicating that the key event is impaired post-translational activation of GSDME rather than transcriptional loss of GSDME. This explains why pyroptosis/GSDME may not appear as a top KEGG pathway in Figure 2A, while GSDME-mediated pyroptosis is nevertheless strongly supported by gasdermin cleavage profiling, ICD markers, GSDME loss- and gain-of-function experiments, and the downstream PPARα–PDK4–ROS–caspase-3 mechanism.

6.Figure 3A–C show that PPARα overexpression or knockout does not significantly affect GSDME cleavage, whereas treatment with PPARα agonists effectively induces GSDME cleavage, indicating a ligand-dependent regulation of GSDME by PPARα. In the patient samples and mouse in vivo models studied here, what are the operative ligands of PPARα?

Response:

We thank the reviewer for this important question. We agree that our data indicate ligand-dependent regulation of GSDME cleavage by PPARα, which raises the question of which endogenous PPARα-activating ligands operate *in vivo*. We would like to clarify that the precise molecular identities of the endogenous PPARα ligands in patient samples or mouse tumors remain to be defined in the present study. Unlike classical hormone receptors with a restricted ligand spectrum, PPARα can be activated by a broad range of lipid species, including fatty acids, eicosanoids, leukotriene B4, and liver-derived phospholipid ligands [PMID: 9113986; 9113987; 9171241; 8900274; 19646743; 20400503]. Therefore, the operative PPARα-activating signal in vivo is likely to reflect a composite lipid-ligand milieu rather than one unique ligand species.

In the original submission, our evidence for ligand-dependent PPARα activation was based on several observations. In mouse HCC tissues, PPARα overexpression markedly upregulated canonical PPARα target genes, including Cpt1a, Fabp1, and Acox1, suggesting that endogenous ligand-like cues are available in vivo. By contrast, PPARα overexpression or deletion alone did not alter these targets or GSDME cleavage under standard in vitro culture conditions. In contrast, pharmacological activation with GW7647 induced PPARα nuclear translocation, upregulated FAO-related PPARα target genes, and triggered the PDK4–mitochondrial ROS–caspase-3–GSDME cascade. These data support the conclusion that PPARα requires ligand-dependent activation to promote GSDME cleavage, but they do not identify the precise endogenous ligand species responsible *in vivo*.

To further address this issue in the revised manuscript, we added a tumor lipid extract–based PPARα activity assay. Total lipids were extracted from FILM and non-FILM mouse HCC tissues by using

Lipid Extraction Kit (Chloroform-Free) (ab211044) and applied to a PPRE-driven luciferase reporter system. Lipid extracts from both FILM and non-FILM tumors activated PPRE reporter activity and induced canonical PPARα target genes, including *Cpt1a*, *Fabp1*, and *Acox1* in Hepa1-6 cells. This activity was attenuated by the PPARα antagonist GW6471, supporting the presence of endogenous PPARα-activating lipid cues in the HCC microenvironment.

Importantly, FILM and non-FILM tumor lipid extracts showed no significant difference in PPRE reporter activation. These findings suggest that FILM-associated suppression of PPARα signaling is unlikely to be caused primarily by reduced endogenous ligand-like activity. Rather, together with our mechanistic data showing that FILM-associated $NOS2^+$ macrophage-derived NO induces SP1 S-nitrosylation and suppresses PPARα transcription in HCC cells (See revised Figure 3J-L), the new reporter assay supports a model in which endogenous PPARα-activating lipid cues remain available, but FILM tumors fail to respond efficiently because tumor-cell PPARα expression is transcriptionally repressed.

7.Fig. 5A uses clodronate liposomes to deplete macrophages. For a more selective assessment, please add an anti-CSF1R–based depletion strategy with appropriate controls and compare the two approaches.

Response:

We thank the reviewer for this constructive suggestion. We agree that although clodronate liposomes are widely used to deplete phagocytic macrophages, an orthogonal receptor-directed depletion strategy would provide a more selective assessment of macrophage involvement.

To further address the reviewer's concern in the revised manuscript, we have now added an anti-CSF1R (BioXcell: BE0213, clone AFS98)–based macrophage depletion strategy with an isotype IgG control. The efficiency of macrophage depletion was validated by immunostaining of tumor-infiltrating $F4/80^+$ macrophages. Consistent with the clodronate liposome results, anti-CSF1R treatment markedly reduced tumor-associated macrophages, restored tumor-cell PPARα expression, increased GSDME cleavage, and enhanced anti-PD-1 responsiveness in FILM tumors (See revised Figure S7B, C). Thus, clodronate liposomes and anti-CSF1R deplete macrophages through distinct mechanisms, namely uptake-mediated cytotoxicity and blockade of CSF1R-dependent survival signaling, respectively. Their concordant effects strengthen the conclusion that macrophages are key cellular mediators of FILM-induced PPARα suppression and anti-PD-1 resistance.

We have updated the text in the Supplementary Materials accordingly (See revised Supplementay Materials page 11 line 28-30, page 12 line 1-7).

8.Fig. 5I profiles M1-associated genes in FILM-CAF–recruited macrophages. Please expand phenotyping to include M2 markers and other polarization signatures. Given that Il1b and Tnfa are also induced alongside Nos2, the potential synergy between these cytokines and NOS2-derived NO merits further investigation.

Response:

We thank the reviewer for this helpful suggestion. To more fully define the macrophage phenotype induced by FILM-derived CAFs, we have expanded the phenotyping beyond the initial M1-associated genes. In the revised manuscript, we now show that FILM-CAF–recruited macrophages upregulate inflammatory genes, including Nos2, Il1b, and Tnfa, together with selected M2/TAM-associated markers such as Arg1, Mrc1, Il10, Tgfb1 (See revised Figure 3E; Figure S7H). These results indicate that FILM-derived CAFs do not generate a classical M1 macrophage population, but instead induce a $NOS2^+$ inflammatory TAM-like state with mixed polarization features.

Regarding the co-induction of Il1b and Tnfa, we directly tested whether these cytokines contribute to tumor-cell PPARα repression. In macrophage–tumor cell co-culture experiments, neutralization of IL-1β (BioXcell: BE0246; Clone: B122) or TNFα (BioXcell: BP0058; clone: XT3.11) did not restore PPARα expression in tumor cells. In contrast, nitric oxide (NO) scavenging (using C-PTIO) or pharmacological inhibition of NOS2 (with NIL) robustly rescued PPARα levels (See revised Figure 3F; Figure S7I-K). These results indicate that NOS2-derived NO is the dominant mediator of PPARα suppression in this setting, while IL-1β and TNFα likely reflect the broader inflammatory activation program of these macrophages.

9. $NOS2^+$ macrophages can arise under non-FILM stimuli (e.g., TLR ligation). Do $NOS2^+$ macrophages increase in abundance specifically within FILM? Please quantify $NOS2^+$ macrophages in both FILM patients and mouse models to establish their prevalence and link to disease course.

Response:

We appreciate the reviewer's point that $NOS2^+$ macrophages can be generated by inflammatory stimuli outside the FILM setting, such as TLR ligation. To directly address whether $NOS2^+$ macrophages are specifically enriched in FILM context, we performed a quantitative comparison of their abundance in FILM versus non-FILM settings using both clinical samples and experimental models in the revised manuscript.

In the human HCC cohort, dual staining for NOS2 and CD68 showed that $NOS2^{+}CD68^{+}$ macrophages were significantly increased in FILM tumors compared with non-FILM tumors. Their abundance was also higher in anti-PD-1 non-responders than responders (See revised Figure S7L, M). In both mouse HCC models, FILM increased the abundance of $NOS2^{+}$ macrophages compared with control liver tumor models. Importantly, these cells were not uniformly distributed throughout the tumor, but were preferentially enriched at the tumor leading edge, where CAF accumulation and collagen-rich stroma were also prominent (See revised Figure 3G). This spatial pattern is consistent with our co-culture data showing that FILM-derived CAFs recruit macrophages and promote a $NOS2^{+}$ inflammatory TAM-like state (See revised Figure 3E).

Together, these data clarify that $NOS2^{+}$ macrophages are not unique to FILM, but they are selectively enriched within the FILM niche in both patients and mouse models.

10.Fig. 5L implicates NO in repressing PPARα expression, but the transcriptional mechanism is not defined. Please delineate the upstream factors and promoter/chromatin events by which NO down-regulates PPARA (e.g., involvement of NF-κB/FOXO/HDACs, S-nitrosylation effects, or enhancer accessibility).

Response:

We appreciate the reviewer's request to define the transcriptional mechanism by which NO represses PPARα expression. In the revised manuscript, we have expanded this part of the study from a NO-dependent phenotype to a promoter-level mechanism linking NOS2-derived NO to impaired PPARα transcription.

To delineate the upstream transcriptional mechanism, we first performed candidate transcription factor prioritization using the hTFtarget database and correlation analysis in TCGA and our HCC cohort. Among predicted regulators of PPARα, we identified a subset of transcription factors positively associated with PPARα expression and then performed a functional siRNA screen. This screen identified SP1, rather than the other candidate transcription factors, as the factor required for FILM-associated macrophages to repress PPARα expression.

We next examined whether NO altered SP1 promoter occupancy. Co-culture with FILM leading-edge macrophages did not significantly change total SP1 expression in HCC cells, arguing against transcriptional level downregulation of SP1. However, ChIP-qPCR showed that SP1 occupancy at the PPARα promoter was markedly reduced after co-culture with FILM-associated macrophages, and this reduction was restored by C-PTIO or NIL. These data indicate that NOS2-derived NO

suppresses PPARα transcription by impairing SP1 promoter binding rather than by decreasing SP1 expression.

To define how NO affects SP1 function, we further examined S-nitrosylation, a canonical NO-mediated post-translational modification. We found that SP1 S-nitrosylation was increased in FILM HCC tissues and in HCC cells co-cultured with FILM-associated macrophages. Treatment of HCC cells with the NO donor SNAP was sufficient to increase SP1 S-nitrosylation, reduce SP1 occupancy at the PPARα promoter, and suppress PPARα mRNA expression; these effects were reversed by C-PTIO. Thus, NO represses PPARα through S-nitrosylation-dependent impairment of SP1 promoter occupancy.

We have revised the Results sections to emphasize this mechanism (See revised Figure 3H-M; revised Main Text page 11 line 8-25). Although NF-κB, FOXO factors, HDACs, and enhancer accessibility may regulate PPARα in other biological contexts, our data support SP1 S-nitrosylation as the proximal mechanism in FILM-associated HCC.

Minor

1.Fig. 2A legend: five NES dot sizes are shown but only four numerical values are provided; please correct the legend.

Response:

We thank the reviewer for pointing this out. We have corrected the Fig. 2A legend so that the NES dot-size scale is consistently labeled with the corresponding numerical values.

2.Fig. 4A labeling: the “GW9578” designation is inconsistent with the main text and caption; please standardize the nomenclature.

Response:

We thank the reviewer for noting this labeling inconsistency. We have corrected “GW9578” to “GW7647” in Fig. 4A to ensure consistency with the main text and figure legend. This change does not affect the interpretation or conclusions of the study.

3.Fig. 5A vs. Fig. 5B: FILM-derived tumor-infiltrating lymphocytes appear to reduce tumor-cell PPARα, yet lymphocyte depletion shows no effect. Please clarify this discrepancy, including timing, subsets targeted, and experimental conditions.

Response:

We thank the reviewer for pointing out this ambiguity. The apparent discrepancy was due to

imprecise terminology in the original figure label. The "FILM-derived TILs" used in Fig. 5A were not purified lymphocytes, but a heterogeneous $CD45^+$ tumor-infiltrating immune cell fraction that included myeloid cells, including macrophages. Thus, the reduction in tumor-cell PPARα expression in this co-culture assay reflects the activity of the mixed immune compartment rather than lymphocytes alone. Consistently, in vivo depletion of lymphoid subsets did not restore PPARα expression, whereas macrophage depletion robustly rescued tumor-cell PPARα levels and GSDME cleavage. We have therefore revised the figure, legend, Methods, and corresponding text to replace "TILs" with "tumor-infiltrating immune cells (TIICs)" and to clarify the experimental conditions.

Reviewer #2 (Remarks to the Author):

The authors of this study try to demonstrate that the fibro-inflammatory liver microenvironment (FILM) fosters NOS2+ macrophages, which suppress PPARα in tumor cells, down-regulating the PDK4–ROS–caspase-3 axis and blocking GSDME-mediated pyroptosis, thereby conferring anti–PD-1 resistance. Ligand activation of PPARα with fenofibrate restores pyroptosis, ignites CD8+ T-cell immunity, and synergizes with anti–PD-1 therapy in multiple murine models. In principle this study would be a good contribution to the field, however, more data and interpretation are needed to support their conclusions and improve the readability of this manuscript.

Major comments:

1. For Figure 1F, could the complete GSEA results be displayed? Is the pyroptosis pathway among the most or one of the most significantly altered pathways?

Response:

We thank the reviewer for this helpful suggestion. We have revised Figure 1F and the corresponding figure legend to make the scope of the analysis clearer. Our intention was not to present an unbiased all-pathway GSEA ranking, but to perform a focused analysis of predefined cell death-related gene sets after observing reduced TUNEL positivity and impaired ICD-associated markers in FILM tumors. We have now clarified this point in the figure legend and revised the presentation to avoid implying that Figure 1F represents a complete all-pathway GSEA output.

Following the reviewer's suggestion, we have revised Figure 1 and the corresponding supplementary data to improve transparency. Instead of showing only individual enrichment plots, we now present the complete focused cell death-related GSEA panel, including pyroptosis, apoptosis, autophagy, necroptosis process and ferroptosis, in both mouse and human HCC tissues (See revised Figure S2D).

When considering the results from mouse and human HCC tissues together, pyroptosis was among the most consistently and prominently suppressed pathways within the focused cell death-related analysis in FILM tumors. We therefore prioritized pyroptosis for subsequent validation, particularly because this transcriptomic finding was supported by orthogonal experimental evidence. Specifically, FILM tumors showed reduced GSDME cleavage, together with decreased membrane CRT and cytoplasmic HMGB1, whereas other gasdermin family members did not show comparable activation. These findings led us to focus on GSDME-mediated pyroptosis rather than other cell death modalities.

2. In Figure 1G, the more prominent GSDME cleavage in Non-FILM tumors seems to result from

higher total GSDME protein expression; tumors with low total GSDME also show correspondingly low GSDME-N levels.

Inflammatory conditions may activate STAT3 and other pathways to up-regulate GSDME expression, whose abundance is a prerequisite for tumor-cell pyroptosis; therefore, the authors should compare total GSDME expression levels between Non-FILM and FILM environments.

Response:

We thank the reviewer for this important comment. To address this concern, we have now quantified total GSDME expression and normalized cleaved GSDME to total GSDME in both Non-FILM and FILM tumors.

In the revised manuscript, full-length GSDME protein was not markedly reduced in FILM tumors compared with Non-FILM tumors. In contrast, the cleaved GSDME fragment and the cleaved/full-length GSDME ratio were significantly decreased in FILM tumors (See revised Figure 1F; Figure S2E). Thus, the lower GSDME-N signal in FILM is not simply attributable to reduced total GSDME abundance, but reflects impaired proteolytic activation of GSDME.

This conclusion is further supported by the anti-PD-1 treatment experiment. Anti-PD-1 increased the cleaved/full-length GSDME ratio in control tumors, whereas this effect was absent in both CCl_4- and MCD-induced FILM tumors despite preserved full-length GSDME expression (See revised Figure 1G). These data indicate that FILM suppresses the upstream caspase-3-dependent activation machinery required for GSDME cleavage rather than merely reducing GSDME protein availability. We have clarified this point in the Results section (See revised Main Text page 8 line 2-10).

3. In Figure 2A, the PPAR pathway is not among the most prominently altered pathways. Why did the authors choose to pursue this pathway for subsequent investigation?

Response:

We thank the reviewer for raising this important point. We have revised the text to clarify that our focus on PPAR signaling was not based solely on absolute pathway ranking in Figure 2A. Although PPAR signaling was not the top-ranked pathway, it showed reproducible downregulation in both mouse and human FILM-HCC tissues and was therefore considered a conserved FILM-associated pathway alteration.

To make this rationale clearer, we have revised the manuscript to display KEGG enrichment results from human HCC tissues and mouse HCC models side by side (See revised Figure 2A). In this revised presentation, PPAR signaling is highlighted in both datasets, demonstrating its consistent

suppression in FILM compared with Non-FILM tumors.

Importantly, PPARα was subsequently prioritized not only because of this cross-species consistency, but also becausePPARα, but not PPARγ or PPARδ, was selectively downregulated, associated with anti-PD-1 response, and functionally linked to GSDME-mediated pyroptosis in our validation experiments. Specifically, restoration or ligand activation of PPARα promoted GSDME cleavage, pyroptotic ICD, and anti-PD-1 sensitization, whereas GSDME loss abolished the sensitizing effect of PPARα. Thus, PPARα was selected as a mechanistically validated upstream regulator of FILM-associated pyroptosis suppression, rather than simply as the highest-ranked enriched pathway.

4. Hepa 1-6 is not an ideal cell line for studying HCC tumor immunology. Hepa1-6 was derived from a C57L mice, which has minor MHC mismatch with C57BL/6 mice. Therefore, at least one additional model—such as the RIL-175 cell line—should be used to validate the conclusions presented in this study.

Response:

We thank the reviewer for this important suggestion. We acknowledge that although Hepa1-6 has been widely used in syngeneic HCC immunology studies, its C57L origin may introduce concerns related to minor histocompatibility mismatch when implanted into C57BL/6 mice. To strengthen the robustness and immunological relevance of our conclusions, we have now performed additional validation experiments using Hep53.4, an independent C57BL/6J-derived murine HCC cell line, which provides a strain-compatible validation model in C57BL/6 hosts.

In the Hep53.4 model, we recapitulated the central findings observed in Hepa1-6 tumors. Specifically, FILM tumors showed impaired anti-PD-1 responsiveness and reduced GSDME cleavage. These results demonstrate that our conclusions are not restricted to Hepa1-6. The new data have been incorporated into the revised manuscript (See revised Figure S1B, S2G), and we have also clarified this point in the Discussion sections (See revised Main Text page 20 line 10-13).

5. The manuscript first establishes that FILM lowers PPARα expression and that this reduction cripples GSDME-mediated pyroptosis and immunotherapy response. Logically, the next question should be how FILM down-regulates PPARα; instead, the authors pivot to detailing how PPARα activation in Non-FILM settings induces pyroptosis, relegating the mechanism of FILM-driven PPARα suppression to a short section at the end. This disrupts narrative flow and weakens overall coherence.

Response:

We thank the reviewer for this insightful comment regarding the narrative structure. We agree that, once FILM-associated PPARα downregulation was identified as a key event suppressing GSDME-mediated pyroptosis and anti-PD-1 responsiveness, the most logical next step was to explain how FILM represses PPARα expression before moving to the downstream metabolic mechanism of PPARα activation.

To improve the coherence of the manuscript, we have reorganized the Results section accordingly. In the revised version, we first establish that FILM suppresses GSDME-mediated pyroptosis and drives anti-PD-1 resistance. We then identify PPARα downregulation as the key tumor-cell-intrinsic event linking FILM to impaired GSDME cleavage and immunotherapy resistance. Immediately after this, we now present the mechanism by which FILM downregulates PPARα, namely the CAF–macrophage–NOS2/NO–SNO-SP1 axis that reduces SP1 occupancy at the PPARα promoter and represses PPARα transcription. The downstream mechanism by which ligand-activated PPARα restores the PDK4–ROS–caspase-3–GSDME axis is then presented after the FILM-driven PPARα suppression mechanism.

We have revised the Results section order, section headings, and transitional sentences throughout the manuscript to make this logic explicit (See revised Main Text page 10-11).

6. The middle section of this manuscript demonstrates that activation, rather than expression level, of PPARα is the decisive trigger for downstream GSDME-pyroptosis, which appears to contradict the earlier claim that FILM suppresses PPARα expression to suppress pyroptosis and promote anti-PD-1 resistance. Is FILM linked to PPARα activation, and if so, how? Clarifying this interplay is essential for coherence.

Response:

We thank the reviewer for pointing out this important issue regarding the relationship between PPARα expression and PPARα activation. We have revised the manuscript to clarify that these two observations are not contradictory.

Mechanistically, FILM is primarily linked to reduced tumor-cell PPARα expression rather than to a direct loss of ligand-dependent PPARα activation machinery. Across both human and mouse cohorts, FILM significantly reduced, but did not completely abolish, PPARα mRNA and protein levels through a macrophage-derived NO-dependent transcriptional repression mechanism. This reduction limits receptor availability and thereby constrains the amplitude of downstream PDK4–ROS–caspase-3–GSDME signaling.

At the same time, PPARα remains a ligand-dependent nuclear receptor. *In vitro*, PPARα overexpression alone was insufficient to induce GSDME cleavage under standard culture conditions, whereas pharmacological activation with GW7647 induced PPARα nuclear translocation, canonical target gene expression, PDK4 induction, mitochondrial ROS accumulation, caspase-3 activation, and GSDME cleavage. *In vivo*, where endogenous lipid cues are present, PPARα overexpression also induced canonical PPARα target genes. Thus, FILM does not necessarily eliminate PPARα ligand activity; rather, it reduces the receptor pool available to respond to endogenous or pharmacological PPARα activation.

We have revised the manuscript accordingly to explicitly clarify this two-layer model and to unify terminology regarding PPARα expression versus activation, thereby improving conceptual coherence throughout the manuscript."

7. FILM lowers PPARα expression and thereby causes anti-PD-1 resistance, and restoring PPARα expression can reverse this resistance. Translational logic would therefore favor agents that re-induce PPARα expression. However, because the authors emphasize that activation—rather than absolute expression level—of PPARα is the critical determinant for inducing tumor-cell pyroptosis, they opt to use PPARα agonists to enhance immunotherapeutic efficacy. And they also predict benefit only in patients whose tumors already express high PPARα. This creates a paradox: if FILM has already suppressed PPARα, how could activation of the residual low-level of PPARa be sufficient to active GSDME-pyroptosis?

Response:

We thank the reviewer for pointing out this important issue. We understand why this may appear paradoxical. Our intention was not to suggest that PPARα agonists can overcome complete loss of PPARα expression. Rather, our data indicate that FILM reduces PPARα expression to different degrees, but does not abolish PPARα in all tumors.

In tumors that still retain detectable PPARα, the remaining receptor can be pharmacologically activated. Under these conditions, GW7647 or fenofibrate restores PDK4 induction, mitochondrial ROS accumulation, caspase-3 activation, GSDME cleavage, and anti-PD-1 sensitization (See revised Figure 4&6). By contrast, when Ppara is genetically deleted in HCC cells, GW7647 no longer effectively suppresses tumor growth or enhances anti-PD-1 efficacy (See revised Figure S18F, G). These results indicate that the effect of PPARα agonists still requires tumor-cell PPARα.

We have therefore revised the manuscript to make the therapeutic implication more precise. PPARα

agonists are not proposed as a universal strategy for all FILM tumors, especially those with profound PPARα loss. Instead, our data support their use in PPARα-retaining tumors, where the residual receptor pool remains druggable. This interpretation is consistent with our humanized PDX experiments, in which fenofibrate enhanced anti-PD-1 efficacy in PPARα-positive tumors but not in PPARα-negative tumors (See revised Figure 6N-P).

We also agree that restoring PPARα expression is an important complementary strategy. Our mechanistic data identify the NOS2–NO–SNO-SP1 axis as one mechanism by which FILM represses PPARα transcription. Therefore, blocking this upstream suppressive pathway may be particularly relevant for tumors with very low PPARα expression. In the present study, we focused on PPARα agonists because clinically available agents such as fenofibrate allowed us to test whether residual PPARα activity in PPARα-retaining tumors could be therapeutically exploited.

8. Although not explicitly stated, the authors' data imply that tumor-cell-intrinsic PPARα is the pivotal switch for therapy-induced apoptosis and pyroptosis, whereas it is clear that PD-1 blockade drives tumor-cell death largely through other immune effector mechanisms.

Response:

We thank the reviewer for this important clarification. We agree that PD-1 blockade does not directly induce tumor-cell death through a tumor-cell-autonomous mechanism alone, but primarily acts by reinvigorating antitumor immune effector mechanisms, including $CD8^+$ T-cell and DC-dependent responses. We have revised the manuscript to avoid implying that tumor-cell PPARα is the sole switch for therapy-induced tumor-cell death.

Our interpretation is that tumor-cell-intrinsic PPARα determines whether immune-mediated antitumor pressure can be efficiently coupled to GSDME-mediated pyroptotic immunogenic cell death. In FILM tumors, suppression of PPARα reduces PDK4 expression, mitochondrial ROS accumulation, caspase-3 activation, and GSDME cleavage, thereby limiting pyroptotic ICD and weakening the amplification of antitumor immunity. Conversely, restoring or pharmacologically activating tumor-cell PPARα reinstates this PDK4–ROS–caspase-3–GSDME axis, allowing anti-PD-1-induced immune effector activity to generate a more immunogenic tumor-cell death response.

Consistent with this model, depletion of $CD8^+$ T cells or DCs attenuated the sensitizing effect of GSDME cleavage on anti-PD-1 therapy, indicating that the therapeutic benefit still depends on immune effector mechanisms. At the same time, tumor-cell Ppara deletion impaired the ability of GW7647 to enhance anti-PD-1 efficacy, whereas GW7647 retained activity in Ppara-deficient

recipient mice bearing PPARα-intact tumors. These data suggest that tumor-cell PPARα is not a replacement for immune effector killing, but rather a tumor-intrinsic determinant that permits immune-mediated killing to proceed through a GSDME-dependent pyroptotic ICD program.

We have revised the Discussion sections to clarify this point and now describe tumor-cell PPARα as a permissive regulator of GSDME-mediated pyroptotic immunogenicity, rather than as the sole driver of anti-PD-1-induced tumor-cell death (See revised Main Text page 18 line 5-12).

Minor comments:

9. For the right-hand panel of Figure 1G, two tumors per group are insufficient to represent the cohort; results from at least five tumors per group should be presented and statistically analyzed.

Response:

We thank the reviewer for raising this point. The right-hand panel of Figure 1G displays representative Western blot results. Due to the technical limitation of Western blot lane capacity (a maximum of 15 lanes per membrane after including controls and molecular markers), we were only able to show two representative tumors per group in the blot itself. However, as the reviewer correctly notes, two samples do not adequately reflect cohort-level variation. To address this concern, we have now included quantitative analysis from all available tumors (n = 6 per group). The statistical summary of GSDME cleavage levels from all tumors is now provided in the revised figure (See revised Figure 1G).

10. The n number of sample size should be stated in the corresponding figure legends.

Response:

We thank the reviewer for the important reminder. As suggested, we have now clearly stated the sample size in the corresponding figure legends, including both the number of tumors shown in representative Western blots and the total number of tumors included in the quantitative analyses. This information has been added to all relevant figures to ensure transparency and reproducibility.

Reviewer #3 (Remarks to the Author):

This manuscript investigates the role of PPARα activation in promoting GSDME-mediated pyroptosis and enhancing anti–PD-1 responsiveness in hepatocellular carcinoma. The topic is conceptually attractive, and the proposed link between metabolic signaling and immunogenic cell death has potential translational significance. However, despite intriguing preliminary findings, the study in its current form lacks the mechanistic depth and experimental transparency required to support its major claims. The data provide correlations rather than causality, and critical methodological information is missing, which limits reproducibility and interpretability.

Major Concerns

1. The central conceptual gap lies in the unproven necessity and mechanistic positioning of GSDME within the PPARα–PD-1 pathway. While the authors propose that PPARα activation induces ROS/caspase-3–dependent GSDME cleavage to enhance anti–PD-1 efficacy, there is no direct evidence demonstrating that GSDME is required rather than a byproduct of metabolic stress. A comprehensive scRNA-seq combined with CITE-seq analysis comparing Gsdme-KO and WT tumor-bearing mice under PPARα agonist treatment is essential to delineate how GSDME loss alters immune-cell composition, activation, and transcriptional networks (e.g., macrophage polarization, dendritic-cell activation, T-cell exhaustion).

This analysis would also reveal the cell-type preference of PPARα activation and the cellular susceptibility to GSDME deletion, providing insight into whether pyroptosis selectively affects particular immune or tumor populations. In addition, UMAP plots visualizing Gsdme expression and KO-dependent clustering shifts should be presented to confirm both the efficiency and cell-type specificity of GSDME deletion within the tumor microenvironment. Please also ensure open access to the raw scRNA-seq and CITE-seq datasets.

Response:

We thank the reviewer for this important suggestion. We agree that defining the functional contribution of GSDME to PPARα agonist-mediated immune remodeling is essential for strengthening the mechanistic conclusion.

To investigate whether GSDME contributes to PPARα agonist-induced immune remodeling during anti–PD-1 therapy, we performed $CD45^+$ immune-cell scRNA-seq on orthotopic FILM-HCC tumors established from control or Gsdme-deficient HCC cells. In the new $CD45^+$ scRNA-seq analysis, unsupervised clustering identified the major tumor-infiltrating immune populations, including T/NK cells, TAMs, DCs, neutrophils, and B cells. Subcluster analyses showed that PPARα agonist treatment shifted T cells toward a proliferative/cytotoxic state and expanded an IFN-responsive DC subset under anti–PD-1 therapy, whereas Gsdme deletion attenuated these changes. In contrast, TAM remodeling induced by PPARα agonist treatment was less affected by Gsdme deletion. These data suggest that GSDME contributes, at least in part, to PPARα agonist-induced

immune activation, including IFN-responsive DC expansion and subsequent T-cell remodeling during anti–PD-1 therapy. Thus, GSDME cleavage is unlikely to be merely a byproduct of metabolic stress, but instead participates functionally in PPARα agonist-mediated enhancement of anti–PD-1 immunity. We have added these data and the corresponding description to the Results section entitled "Pharmacological PPARα Activation Enhances Anti–PD-1 Therapy Efficacy in HCC".

We agree that CITE-seq would provide additional single-cell protein-level resolution of immune-cell states. However, CITE-seq requires prospective sample preparation, antibody-panel optimization, and additional sequencing, which would require further technical optimization and a sufficient experimental timeline. Therefore, to address the reviewer's mechanistic concern in a feasible and directly interpretable manner, we performed $CD45^+$ immune-cell scRNA-seq to define immune-cell transcriptional states.

Regarding the reviewer's request for UMAP visualization of Gsdme expression and knockout specificity, we would like to clarify that the newly added scRNA-seq experiment was designed to specifically characterize immune-microenvironmental remodeling. Therefore, we sorted $CD45^+$ tumor-infiltrating immune cells for sequencing, which allowed higher-resolution analysis of T-cell, TAM, and DC states under anti–PD-1 plus PPARα agonist treatment. Because tumor cells were not included in this $CD45^+$ immune-cell-enriched dataset, it would not be appropriate to use these data to evaluate tumor-cell Gsdme expression or knockout efficiency. Instead, GSDME knockout efficiency was validated in the relevant tumor-cell compartment. These assays provide direct confirmation of GSDME depletion in tumor cells, whereas the $CD45^+$ scRNA-seq data define the downstream immune consequences of tumor-cell GSDME loss.

We have incorporated these new data into the revised manuscript as Figure S20 and revised the Results, Methods, and figure legends accordingly. Raw and processed scRNA-seq data have been deposited in the Genome Sequence Archive (GSA) at the National Genomics Data Center, China National Center for Bioinformation, under accession number CRA043152.

2. The manuscript lacks critical details regarding mouse strains, sex, age, and genotyping of Gsdme-KO mice, as well as sources, catalog numbers, and doses of anti–PD-1 antibodies, GW7647, fenofibrate, and viral vectors (LV, AAV). These omissions preclude evaluation of reproducibility and raise concerns about data reliability. All key materials and their validation (e.g., knockout confirmation, vector construction maps, titers) should be explicitly stated.

Response:

We thank the reviewer for pointing out these important omissions. We recognize that these methodological details are essential for reproducibility, and we have now substantially expanded the Methods section and added a new Supplementary Table summarizing all key materials used in the study.

In the revised manuscript, we now explicitly state the mouse strain background, sex, age range, source, housing conditions, and experimental allocation for each *in vivo* model. We have also provided detailed information for all major reagents, including the source, catalog number, clone, dose, route, and treatment schedule of anti-PD-1 antibody; the source, catalog number, dose, solvent, route, and dosing frequency of GW7647 and fenofibrate; and the construction, source, titer, dose, and delivery route of LV and AAV vectors. For viral vectors, we now include the backbone information, inserted sequence or shRNA/sgRNA target sequence, promoter, vector map where applicable, and viral titer.

These revisions have been incorporated into the Methods section and summarized in Supplementary Table S5-11. We believe these additions improve transparency and reproducibility and address the reviewer's concern regarding material validation and data reliability (See revised Supplementay Materials page 59-68)..

3. This work lacks raw data and data-sharing transparency. No accession numbers are provided for RNA-seq or other omics datasets. Raw Western blot images, original flow-cytometry files, and unprocessed microscopy data are not included. Public deposition of sequencing data (GEO/ArrayExpress) and provision of uncropped images are mandatory for reproducibility.

Response:

We thank the reviewer for raising this important point. In the revised manuscript, we have substantially expanded the data availability information and provided access to the original datasets and unprocessed source files.

Specifically, Raw and processed scRNA-seq data have been deposited in the Genome Sequence Archive (GSA) at the National Genomics Data Center, China National Center for Bioinformation, under accession number CRA043152. Human bulk RNA-seq data are deposited in the Genome Sequence Archive for Human (GSA-Human) under accession number HRA013521. Mouse bulk RNA-seq data are deposited in the Genome Sequence Archive (GSA) under accession number CRA030675. In addition, the raw and uncropped Western blot images corresponding to all immunoblot panels have been included as Supplementary Raw Images.

We have revised the Data availability section accordingly to clearly describe where each category of raw and processed data can be accessed. We believe these additions substantially improve the transparency and reproducibility of the study.

4. The linear signaling cascade is overstated. Current evidence shows correlation but not causation between PPARα activation, PDK4 induction, ROS accumulation, and GSDME cleavage. Rigorous loss- and gain-of-function validation (PDK4 knockout, kinase-dead rescue, PDH activity, mitochondrial respiration, and metabolic-flux assays) is required to establish PDK4 as a mechanistic intermediary.

Response:

We thank the reviewer for this important critique. We agree that the initial presentation may have made the PPARα–PDK4–ROS–GSDME axis appear overly linear, and that correlation alone would not be sufficient to establish PDK4 as a mechanistic intermediary. We have therefore revised the text to avoid overstatement and have added loss- and gain-of-function experiments to directly test the causal role of PDK4 in this pathway.

In the original version, we identified PDK4 as a candidate downstream effector of ligand-activated PPARα based on transcriptomic screening and showed that PDK4 expression was reduced in FILM tumors and anti-PD-1 non-responders. In the revised manuscript, we have now strengthened this conclusion with functional validation. First, silencing PDK4, but not the other candidate ROS-related genes APOA4 or CYP1B1, markedly impaired GW7647-induced mitochondrial ROS accumulation. PDK4 knockdown also attenuated GW7647-induced membrane blebbing, LDH/HMGB1 release, Annexin V^{+}7-AAD^{+} tumor-cell death, and GSDME cleavage (See revised Figure 5E; Figure S15G, H).

To further place PDK4 mechanistically between PPARα activation and mitochondrial redox stress, we examined the PDK4–PDH axis. GW7647 increased phosphorylation of the PDH E1α subunit and reduced PDH enzymatic activity in control HCC cells, whereas these effects were largely lost after PDK4 knockdown. We then generated PDK4-knockout cells and reconstituted them with either wild-type PDK4 or a kinase-dead PDK4 mutant.The KD mutant was generated by substituting Asp394 and Trp395 with alanine, as previously described (Wynn RM et al., PMID: 18658136). Re-expression of wild-type PDK4 restored GW7647-induced PDH phosphorylation, mitochondrial ROS elevation, and GSDME cleavage, whereas kinase-dead PDK4 failed to do so despite comparable protein expression (See revised Figure S16). These rescue experiments demonstrate that the kinase activity of PDK4 is required for the metabolic-redox and pyroptotic effects downstream

of PPARα activation.

Based on these new data, we have revised the Results sections to state more precisely that ligand-activated PPARα transactivates PDK4, and that PDK4 kinase activity is required for PDH inhibition, mitochondrial ROS accumulation, caspase-3 activation, and GSDME cleavage (See revised Main Text page 14 line 1-11).

5. ROS is interpreted as an upstream trigger of caspase-3 activation, yet PPARα primarily regulates transcriptional programs rather than acute oxidative stress. Without time-course or mitochondrial functional data, the claim that ROS precedes caspase-3 activation is speculative. Specific genetic and temporal assays should be used to define this hierarchy.

Response:
We thank the reviewer for this important and technically rigorous comment. To address the reviewer's concern and to define the temporal hierarchy more rigorously, we have added both time-resolved and genetic functional data in the revised manuscript.

To define the hierarchy more rigorously, we added a time-course analysis after GW7647 treatment. Mitochondrial ROS increased at earlier time points, whereas cleaved caspase-3 and cleaved GSDME became detectable later (See revised Figure S14A, B). This temporal pattern supports a sequence in which ligand-activated PPARα first induces metabolic-redox remodeling, followed by caspase-3 activation and GSDME cleavage. Importantly, PPARα deletion impaired the entire GW7647-induced ROS–caspase-3–GSDME response, indicating that this cascade depends on tumor-cell PPARα (See revised Figure S14A, B).

We also strengthened the pathway using genetic and metabolic perturbations. Antioxidant blockade with NAC or MitoTEMPO prevented GW7647-induced membrane blebbing and LDH/HMGB1 release. Inhibition of CPT1-dependent FAO with etomoxir or CPT1 knockdown reduced mitochondrial ROS accumulation, caspase-3 activation, GSDME cleavage, and pyroptotic cell death (See revised Figure 4G, H; Figure S14C, D).

These new results have been incorporated into the revised manuscript and clarify the hierarchical relationship between PPARα activation, ROS generation, and caspase-3–dependent GSDME cleavage (See revised Main Text page 12 line 29-30; page 13 line 1-10).

6. The identification of Annexin V^+/7-AAD^+/$CD45^-$ cells as pyroptotic is not justified, as this

phenotype typically marks late apoptosis. Definitive evidence—such as co-localization of cleaved GSDME, IL-1β/IL-18 release, or real-time membrane-integrity assays—is required to confirm true pyroptosis.

Response:

We agree with the reviewer that Annexin V^+/7-AAD^+ staining is not a pyroptosis-specific marker and can also reflect late apoptosis or secondary necrosis. We apologize for any over-interpretation in the original text.

We note, however, that Annexin V^+/7-AAD^+ staining has been widely used in the literature as a surrogate readout of terminal membrane rupture associated with gasdermin-mediated pyroptosis, particularly in studies focusing on GSDME-dependent cell death (PMID: 39486886). In the revised manuscript, Annexin V^+/7-AAD^+/$CD45^-$ cells are described more conservatively as membrane-disrupted tumor cells, rather than as pyroptotic cells per se. We used this staining only as a complementary readout of tumor-cell membrane permeabilization. The assignment of this phenotype to GSDME-mediated pyroptosis is based on convergent evidence, including GSDME cleavage, caspase-3 dependence, LDH/HMGB1 release, and pyroptotic morphology. Importantly, the increase in membrane-disrupted tumor cells and the accompanying LDH/HMGB1 release were abolished by GSDME deletion, supporting that this membrane-disruptive phenotype depends on GSDME in our experimental setting. We have revised the manuscript accordingly to clarify this point and avoid overstatement (See revised Main Text page 9 line 15-16, 24-25; page 12 line 6-7).

7. The identification of $NOS2^+$ macrophages as the major immunosuppressive population relies on immunostaining and limited in-vitro assays. Without single-cell profiling or multiplex spatial validation, it remains unclear whether $NOS2^+$ cells form a distinct macrophage subset or a transient activation state. Moreover, the mechanism by which NO suppresses PPARα (direct S-nitrosylation vs. secondary cytokine effects) is not experimentally addressed.

Response:

We appreciate the reviewer's insightful comments regarding the identity of $NOS2^+$ macrophages and the mechanism by which NO suppresses PPARα. Our revised manuscript supports a more precise interpretation: $NOS2^+$ macrophages represent a FILM-enriched inflammatory TAM-like activation state that is spatially enriched at the tumor leading edge and functionally linked to PPARα repression.

We agree that single-cell profiling or high-plex spatial assays could further refine macrophage taxonomy. However, our study does not rely on $NOS2^+$ cells being a stable lineage; rather, we

demonstrate functional necessity of NOS2⁺ macrophages in FILM-mediated immunosuppression. In the revised manuscript, we therefore clarify that macrophages-derived NO represents a functionally dominant inflammatory activation state that is selectively enriched within the FILM. Importantly, quantitative analyses in both human HCC specimens and mouse models demonstrate a significant increase in the proportion of $NOS2^+$ macrophages specifically in FILM tumors, particularly at the tumor margin, compared with non-FILM controls (See revised Figure 3G; Figure S7L). These data establish $NOS2^+$ macrophages as a prevalent and spatially organized immunosuppressive population in FILM, independent of whether they represent a stable subset or a dynamic activation state.

Regarding the mechanism by which $NOS2^+$ macrophages suppress PPARα, we have now clarified that this effect is primarily mediated by NOS2-derived NO rather than by secondary inflammatory cytokines. In co-culture experiments, the NO scavenger C-PTIO or the NOS2 inhibitor NIL restored PPARα expression in HCC cells exposed to leading-edge macrophages isolated from FILM tumors or to BMDMs polarized by FILM tumor-derived CAFs (See revised Figure 3F; Figure S7I). In contrast, neutralization of IL-1β or TNFα did not restore PPARα expression (See revised Figure S7J, K).

To define how NO represses PPARα transcription, we next examined transcription factors predicted to regulate PPARα. hTFtarget analysis identified 29 candidate transcription factors, among which 10 showed consistent positive correlations with PPARα expression in both TCGA and our HCC cohort. A functional siRNA screen further identified SP1 as the key factor required for FILM leading-edge macrophage-mediated suppression of PPARα; knockdown of SP1, but not other candidate transcription factors, abolished this suppressive effect (See revised Figure 3H, I; Figure S9E).

We then asked whether NO altered SP1 abundance or its promoter-binding activity. Co-culture with FILM leading-edge macrophages did not significantly change total SP1 expression in HCC cells, but markedly reduced SP1 occupancy at the PPARα promoter. This reduction was reversed by C-PTIO or NIL, indicating that NOS2-derived NO impairs SP1 promoter binding rather than decreasing SP1 expression (See revised Figure 3J; Figure S9F). Because NO commonly regulates protein function through S-nitrosylation, we next examined SP1 S-nitrosylation. S-nitrosylated SP1 was increased in FILM HCC tissues and in HCC cells co-cultured with FILM leading-edge macrophages. Moreover, treatment with the NO donor SNAP was sufficient to rapidly increase SP1

S-nitrosylation, reduce SP1 occupancy at the PPARα promoter, and decrease PPARα mRNA expression (See revised Figure 3L, M). These effects were reversed by C-PTIO, supporting a direct NO-dependent mechanism.

Together, these results indicate that FILM-associated leading-edge macrophages repress PPARα transcription through a NOS2-derived NO–SNO-SP1 mechanism, in which S-nitrosylation of SP1 impairs its occupancy at the PPARAα promoter and thereby suppresses PPARα expression.

We have revised the Results sections to make this mechanism explicit and to distinguish it from indirect cytokine-mediated repression (See revised Main Text page 10 line 23-30; page 11 line 1-25).

8. Because PPARα agonists affect multiple cell types, it is unclear whether the observed pyroptosis and PD-1 sensitization originate from tumor-intrinsic or immune-cell–intrinsic PPARα activity. Tumor-specific versus host-specific knockout or bone-marrow transplantation experiments are required to delineate cell-type specificity.

Response:

We thank the reviewer for highlighting this important issue of cell-type specificity. Because PPARα agonists can indeed act on multiple cellular compartments, we have added reciprocal genetic experiments to determine whether the therapeutic effect of GW7647 is primarily mediated by tumor-cell or host-derived PPARα.

First, we deleted Pparα in HCC cells and implanted control or Pparα-deficient tumor cells into immunocompetent WT recipient mice. In this setting, loss of tumor-cell Ppara markedly impaired the ability of GW7647 to suppress tumor growth and enhance anti-PD-1 efficacy (Figure S18F, G). This result indicates that tumor-cell Pparα is required for the pyroptosis-promoting and anti-PD-1–sensitizing effect of GW7647.

Second, we performed the reciprocal experiment by implanting Pparα-intact HCC cells into WT or Pparα$^{-/-}$ recipient mice. In Ppara$^{-/-}$ recipients, GW7647 no longer induced host FAO-related gene expression, confirming loss of host PPARα responsiveness. Nevertheless, GW7647 retained its antitumor activity and its ability to enhance anti-PD-1 efficacy when the implanted tumor cells still expressed Pparα (Figure S18H-K). These data indicate that host-derived PPARα is largely dispensable for the major therapeutic effect observed in our model.

Together, these reciprocal experiments support the conclusion that GW7647-mediated GSDME activation and anti-PD-1 sensitization are driven predominantly by tumor-cell-intrinsic PPARα rather than immune-cell- or host-intrinsic PPARα activity. We have revised the Results accordingly (See revised Main Text page 15 line 16-20).

9. Randomization, blinding, and sample-size justification are not described. Several figures display only mean ± SD without individual data points, hindering evaluation of variance and significance. Statistical parameters and exact n values should be clearly reported.

Response:

We thank the reviewer for pointing out these important issues regarding statistical transparency and reproducibility. We have revised the manuscript, figure legends, and supplementary information to provide clearer information on randomization, blinding, sample size, and statistical reporting.

In the revised Methods, we now state that mice were randomly assigned to treatment groups after tumor establishment based on comparable baseline tumor burden or bioluminescence intensity. Histological quantification, immunostaining analysis, and flow-cytometric gating were performed with investigators blinded to group allocation whenever feasible. We also added exclusion criteria, including procedure-related death before treatment initiation or failure to establish detectable tumors before randomization. Sample sizes were chosen based on prior experience with orthotopic HCC models and were designed to provide sufficient power to detect biologically meaningful differences in tumor burden and immune readouts while minimizing animal use (See revised Supplementay Materials page 11 line 6-16).

We have also revised the figure legends to report exact n values, the definition of error bars, and the statistical tests used for each comparison. For *in vivo* experiments, n now refers to the number of biologically independent mice. For *in vitro* experiments, n refers to independent biological replicates unless otherwise specified. We have also clarified whether data are shown as mean ± SD or mean ± SEM and have made this consistent across the manuscript. To improve visualization of variance, we have revised the relevant figures to display individual data points together with summary statistics whenever possible, rather than showing only bar graphs with mean ± SD.

These revisions have been incorporated into the Statistical analysis section, figure legends, and Supplementary materials..

Minor Points

1. Inadequate citation support in the Introduction. Key statements rely on review articles rather than primary mechanistic studies (e.g., Refs 17–18 and 22–23). Original research citations should replace reviews to substantiate claims regarding GSDME's role in immunotherapy and expression in HCC.

Response:

We thank the reviewer for this important suggestion. We agree that primary mechanistic studies provide stronger support for the statements regarding GSDME-mediated pyroptosis, its role in antitumor immunity/immunotherapy responsiveness, and its expression relevance in HCC. Accordingly, we have carefully revised the Introduction and replaced the previously cited review articles with original research articles. Specifically, the references supporting the role of GSDME in pyroptosis-mediated antitumor immunity and immunotherapy response have been updated to Refs. 18–20, and the references supporting GSDME expression and functional relevance in HCC have been updated to Refs. 24–25. These changes have strengthened the citation basis for the relevant statements in the Introduction (See revised Main Text page 23).

2. Typographical and nomenclature errors. For example, "PAPRγ" should read "PPARγ." Check all figures and legends carefully for consistency.

Response:

We thank the reviewer for pointing out the typographical and nomenclature errors. We apologize for these oversights. In the revised manuscript, we have corrected "PAPRγ" to "PPARγ" and have carefully re-checked all figures, figure legends, main text, and supplementary materials for spelling, gene/protein nomenclature, and symbol consistency (e.g., PPARα/PPARγ/PPARδ, GSDME, PD-1, IFN-γ). All identified errors have now been corrected.

3. Missing methodological details. Include antibody sources and dilution, RNA-seq analysis pipeline, normalization method, and statistical tests used for gene-expression comparisons.

Response:

We thank the reviewer for identifying these missing methodological details. We have expanded the Methods and Supplementary Materials and Methods to improve methodological transparency. Specifically, we have added Supplementary Tables summarizing all antibodies used in this study, including supplier, catalog number, clone where applicable, application and dilution. This includes antibodies used for western blotting, immunohistochemistry, immunofluorescence, multiplex immunofluorescence, ChIP-qPCR and flow cytometry (See revised Supplementay Materials page 61-64). We have also updated the RNA-seq analysis pipeline in the Supplementary Materials (See revised Supplementay Materials page 5-6).

4. Data presentation. Provide individual datapoints in bar graphs, specify number of biological replicates, and show representative uncropped Western blots.

Response:

We thank the reviewer for pointing out this issue. We have revised the data presentation throughout the manuscript to improve transparency and allow better assessment of variance.

In the revised figures, bar graphs now display individual data points whenever applicable, together with the summary statistics. We have also updated all figure legends to specify the exact number of biological replicates for each experiment. In addition, representative uncropped Western blots have been provided in the revised source data file. The corresponding cropped blots in the main figures are now linked to their uncropped versions.

5. Clinical cohort limitations. The retrospective analysis (14 vs 25 patients) is underpowered; authors should tone down translational claims and describe inclusion criteria and potential confounders.

Response:

We thank the reviewer for this important comment. We agree that Cohort #3 was limited by its retrospective design and small sample size and therefore was not sufficiently powered to draw definitive clinical conclusions. In the revised manuscript, we have toned down the translational claims and now describe the clinical findings as exploratory and hypothesis-generating. We also revised the Supplementary Methods to clarify the eligibility criteria for Cohort #3. Specifically, Cohort #3 was defined as a retrospective exploratory cohort of 39 patients with clinically or pathologically diagnosed HCC who received anti-PD-1-based systemic therapy with or without concomitant fenofibrate between January 2020 and January 2024. Eligible patients had documented anti-PD-1 treatment records, available baseline and follow-up radiological imaging, and sufficient follow-up information for response assessment according to iRECIST. Fenofibrate exposure was defined as documented fenofibrate use concomitant with anti-PD-1 therapy before radiological response assessment.

We further clarified that baseline clinicopathological variables and potential confounders, including age, sex, HBV infection, ECOG performance status, Child-Pugh class, tumor number, tumor size, BCLC stage, and AFP level, are summarized in Supplementary Table 3. We also added a limitation statement noting that, despite comparison of these baseline variables, the retrospective design, limited sample size, potential selection bias, treatment heterogeneity, and unmeasured confounders

may affect interpretation of the clinical association.

Given the small sample size, we have avoided overfitting multivariable models. We have also added a statement in the Discussion that the 14 versus 25 comparison is underpowered, retrospective, and susceptible to treatment-selection bias. A prospective biomarker-stratified trial will be required to determine whether fenofibrate truly improves anti-PD-1 efficacy in PPARα-retaining HCC (See revised Main Text page 16 line 15-17, page 20 line 13-18).

6. Drug selectivity. Discuss pharmacological differences between GW7647 and fenofibrate and clarify whether both act via identical mechanisms.

Response:

We thank the reviewer for raising this important pharmacological point. We have revised the manuscript to distinguish more clearly between GW7647 and fenofibrate and to avoid implying that these two agonists are pharmacologically identical.

GW7647 was used in this study primarily as a potent and relatively selective experimental PPARα agonist for mechanistic interrogation. It has been reported as a high-affinity PPARα agonist with approximately 200-fold selectivity over PPARγ and PPARδ, and commonly serves as a tool compound to activate PPARα in mechanistic studies [PMID: 11354382]. By contrast, fenofibrate is a clinically approved fibrate used for dyslipidemia. It is a prodrug that is converted to fenofibric acid, and it has lower potency than GW7647 and may exert broader metabolic and cell-context-dependent effects [PMID: 15790930].

we have revised the Discussion to acknowledge that fenofibrate may not act exclusively through PPARα and may have additional metabolic or PPARα-independent effects (See revised Main Text page 20 line 20-25).

Reviewer #4 (Remarks to the Author):

Peng Chen et al. describe an interesting study of the mechanisms underlying the fibroinflammatory liver microenvironment (FLIM)-driven immunosuppression in hepatocellular carcinoma. They provide evidence that NOS2+ macrophages suppress PARP□ expression/activation, which lowers PKD4 levels, leading to decreased ROS levels, further suppressing caspase-3/GSDME-mediated pyroptosis. This is a well-designed study using omics analyses of available clinical samples as well as the validation of the mechanisms in multiple preclinical mouse models. By retrospective analysis of clinical cohorts and utilizing preclinical FLIM-HCC models, the authors demonstrate that PARP□ activator fenofibrate improves anti-PD1 therapy.

This is a timely study, demonstrating an interesting mechanism by which even an inflammatory environment can lead to suppression of immunogenic cell death. I only have some minor comments.

1. Fig 1G: In human samples, it seems that full-length GSDME as well as GSDMA (Fig. S2C) expression is higher in non-FLIM samples 1-3, which then directly correlates with increased levels of the NT fragment of GSDME. The author should analyze the effect of FLIM on FL GSDME expression and the effect of anti-PD1 therapy on it as well. The effect of anti-PD1 therapy in the mouse model is more telling, although one cannot miss that anti-PD-1 treatment boosts FL GSDME levels.

Response:

We thank the reviewer for this careful observation. We have now performed additional densitometric quantification to clarify whether the difference in GSDME-N reflects altered total GSDME abundance or altered cleavage efficiency.

Using the Western blots shown in Fig. 1F–G, we quantified full-length GSDME normalized to the loading control, as well as the GSDME-N/full-length GSDME ratio. In human HCC samples, full-length GSDME showed some inter-sample variation, as noted by the reviewer, but its overall level was not significantly reduced in FILM tumors compared with non-FILM tumors. In contrast, the GSDME-N/full-length GSDME ratio was significantly decreased in FILM tumors. These data indicate that FILM primarily impairs GSDME cleavage efficiency rather than simply reducing total GSDME expression.

We also re-examined GSDMA expression in Fig. S2C. Although GSDMA showed heterogeneous variation among individual samples, FILM did not produce a statistically significant or consistent change in full-length GSDMA expression, particularly in the mouse models. Moreover, we did not observe a FILM-associated cleavage pattern for GSDMA comparable to that of GSDME. We therefore focused subsequent functional validation on the GSDME axis (See below).

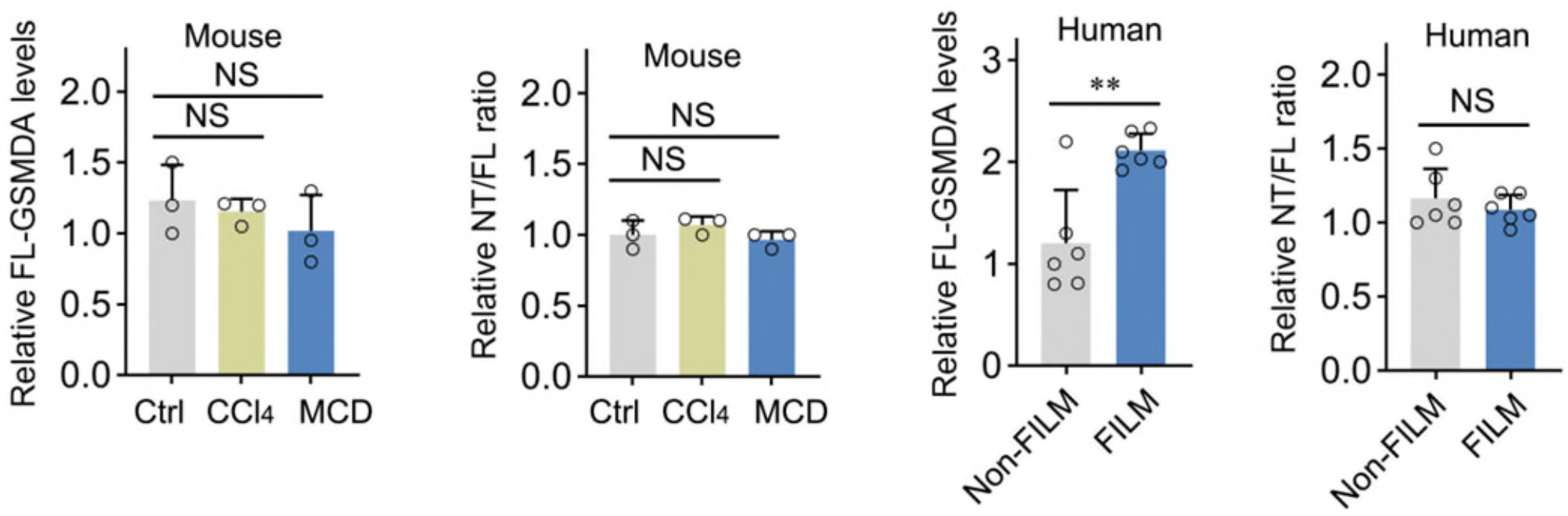

In the mouse experiments, we agree with the reviewer that anti-PD-1 treatment modestly increased full-length GSDME expression. This may be compatible with immune-activation-associated regulation of GSDME, as reported in recent studies linking IFNγ/STAT1 signaling to GSDME-associated pyroptotic programs in HCC [PMID: 39486886]. Importantly, however, increased full-length GSDME did not uniformly translate into GSDME-N generation. Robust GSDME cleavage and an increased GSDME-N/full-length GSDME ratio were observed only in control-liver tumors after anti-PD-1 treatment, whereas FILM tumors failed to efficiently generate GSDME-N despite retaining detectable full-length GSDME

.

We have revised Fig. 1F–G and Fig. S2 to include quantification of full-length GSDME, and the GSDME-N/full-length GSDME ratio, and have clarified in the Results section that FILM suppresses proteolytic activation of GSDME rather than merely reducing total GSDME abundance (See revised Main Text page 8 line 2-10).

2. A surprising result is relatively low cytotoxicity due to exogenously provided NT-GSDME. Is FLIM also suppressing NT-GSDME pore-forming activity? This experiment, however, requires a bit more detailed technical description. It is unclear how the transduced cell line was prepared. Introduction of constitutively expressed NT-GSDME would lead to immediate cell death. Please provide a detailed description in the methods (plasmid preparation, cell line transduction and animal model).

Response::

We thank the reviewer for raising this important technical and conceptual point. We have revised the manuscript to clarify both the interpretation of the NT-GSDME rescue experiment and the method used to generate the corresponding tumor cells.

Our intention was not to suggest that FILM suppresses the intrinsic pore-forming activity of already generated NT-GSDME. Rather, our data indicate that FILM suppresses the upstream activation step required for endogenous GSDME cleavage. Expression of NT-GSDME was used as a bypass

experiment to test whether restoring the active GSDME fragment could rescue the pyroptosis-deficient and anti-PD-1–resistant phenotype in FILM tumors.

We also recognize that constitutive high-level expression of NT-GSDME would be expected to cause rapid membrane rupture and cell death. To avoid this issue, NT-GSDME was expressed using a controlled lentiviral system rather than by constitutive high-level expression during cell-line establishment. Briefly, the N-terminal fragment of mouse GSDME was cloned into a doxycycline-inducible lentiviral vector. HCC cells were transduced and selected in the absence of doxycycline, allowing stable expansion before induction of NT-GSDME expression. NT-GSDME expression was then induced during tumor growth, as described in the revised Methods. This strategy allowed us to generate viable tumor cells while restoring GSDME pore-forming activity *in vivo*.

We have added a detailed description of the NT-GSDME vector construction, lentiviral transduction, selection, induction, validation, and orthotopic implantation procedure in the Supplementary Materials section (See revised Supplementay Materials page 9 line 12-20).

3. Fig. 5A: The labelling GW7647/Fenofibrate is misleading. Figs. 5F and 5O: The authors should provide quantification of cleaved GSDME levels, if possible, also by western blot. Fig. 5I: It seems that fenofibrate worsens the effect of antiPD1 therapy, or are the curves mislabeled?

Response:

We thank the reviewer for these helpful comments regarding figure labelling and data presentation. We have carefully rechecked the corresponding raw data and revised Figure 5 accordingly.

First, we have corrected the labelling in Fig. 5A. In the original version, “GW7647/Fenofibrate” was intended to indicate PPARα agonist-based treatment in general. However, only GW7647 was used in this specific murine experiment, and we agree that the original label was potentially misleading. In the revised figure, this panel is now labelled as “GW7647”, and the figure legend has been updated to clearly specify which PPARα agonist was used in each experiment (See revised Figure 6A).

Second, we have added quantification of cleaved GSDME levels in the revised Fig. 6D and Fig. 5L. These additions make the increase in GSDME activation after GW7647 or fenofibrate treatment, particularly in combination with anti-PD-1, more transparent (See revised Figure 6D, L).

Third, we re-examined the raw data for Fig. 5I and identified a labelling inconsistency in the original

figure. The group labels and curve colours have now been corrected. In the revised version, the fenofibrate plus anti-PD-1 group correctly shows improved antitumor efficacy compared with anti-PD-1 alone, consistent with the raw data and the other therapeutic readouts (See revised Figure 6H).

4. If the ROS are behind the activation of caspase-3/GSDME pyroptosis, could ROS-inducing drugs provide the same effect (skipping PARP/PKD4 steps)?

Response:

We thank the reviewer for raising this important conceptual question. We have clarified in the revised manuscript that ROS is required for GW7647-induced caspase-3/GSDME activation, but nonspecific ROS elevation is not sufficient to fully recapitulate this process.

To test this directly, we treated HCC cells with mitochondrial ROS-inducing agents, including rotenone and antimycin A. These agents increased mitochondrial ROS, but they did not efficiently induce LDH/HMGB1 release (See below).

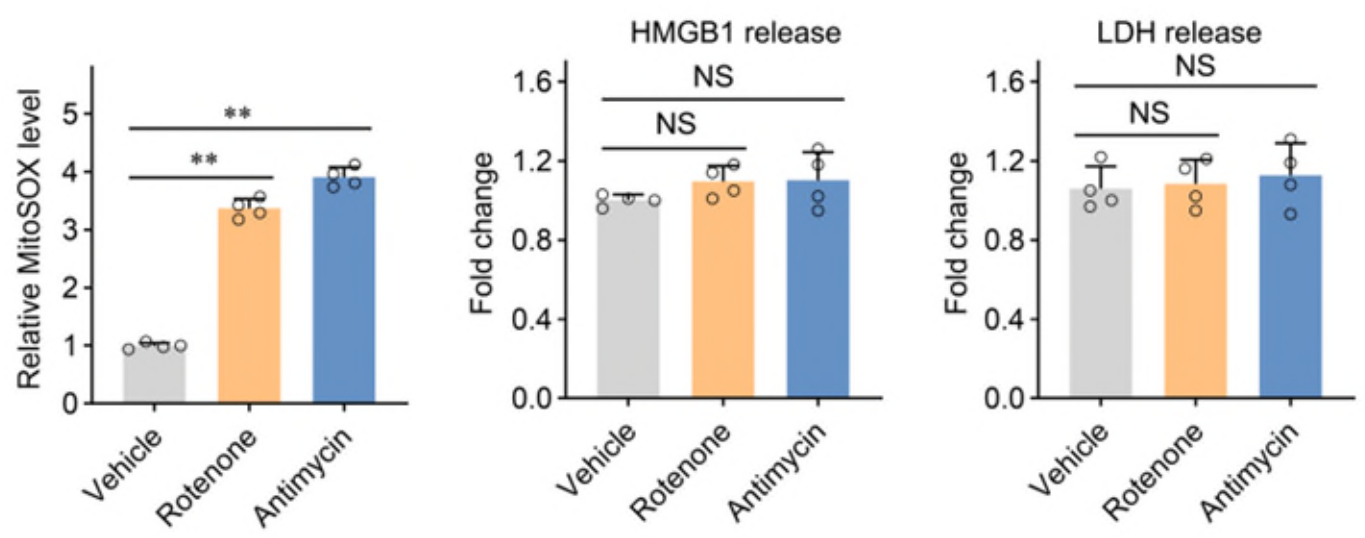

These results indicate that ROS functions as a necessary component of the pathway, but that generic mitochondrial oxidative stress alone does not reproduce PPARα-driven GSDME pyroptosis. This distinction is important because ligand-activated PPARα does not simply increase ROS acutely. Instead, it transcriptionally induces PDK4 and rewires mitochondrial metabolism through PDH inhibition and FAO-associated metabolic remodeling. This PPARα–PDK4-dependent metabolic-redox state precedes caspase-3 activation and supports subsequent GSDME cleavage.

Thus, our data suggest that the biological effect of ROS depends on its metabolic context, timing, and integration with PPARα-dependent transcriptional remodeling. ROS-inducing drugs may generate oxidative stress, but under the conditions tested they do not bypass the requirement for the PPARα–PDK4 axis to produce efficient caspase-3/GSDME-mediated pyroptotic ICD.

5. Perhaps the steps downstream of PKD4 could be elaborated a bit more, at least in the discussion? How does ROS induce activation of caspase-3? Is this caspase-9 initiated cascade?

Response:

We thank the reviewer for raising this mechanistic question. Our current data support mitochondrial ROS as an upstream contributor to caspase-3/GSDME activation. Mitochondrial ROS increased before detectable caspase-3 cleavage and GSDME activation after GW7647 treatment, and blockade of ROS with NAC or MitoTEMPO suppressed GW7647-induced membrane disruption and LDH/HMGB1 release. These findings, together with CPT1 inhibition/knockdown and PDK4 loss- and rescue-of-function experiments, support a model in which PPARα–PDK4-dependent metabolic remodeling generates mitochondrial redox stress upstream of caspase-3 activation.

To address the reviewer's question regarding caspase-9, we performed an additional control experiment using the caspase-9 inhibitor z-LEHD-FMK. Caspase-9 inhibition partially reduced GW7647-induced LDH and HMGB1 release (See below), suggesting that caspase-9 may contribute to the connection between mitochondrial ROS and caspase-3/GSDME activation. However, because this inhibition was partial and caspase-9 was not the main mechanistic focus of the study, we did not present caspase-9 as an obligatory upstream mediator in the main model.

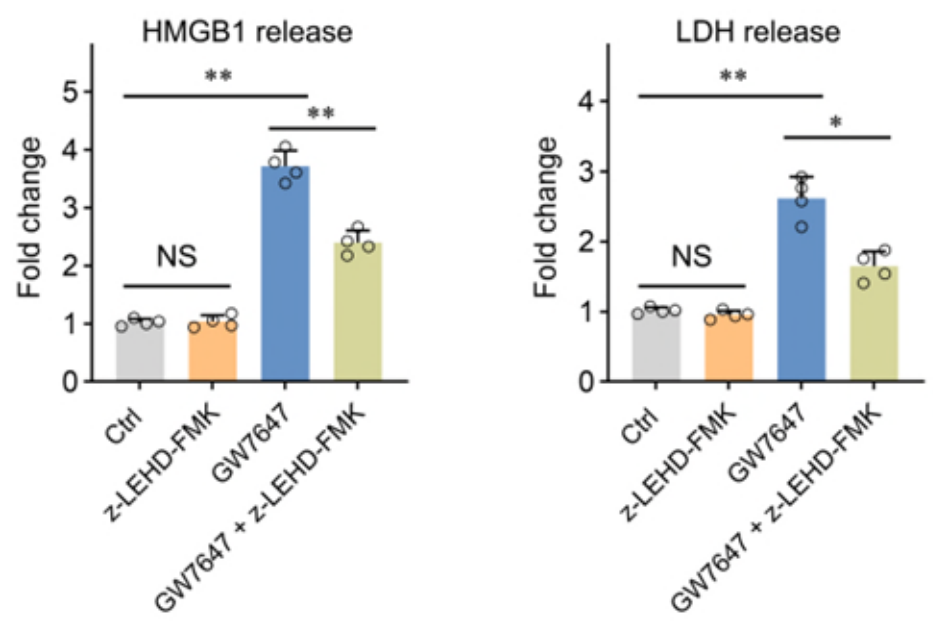

We have revised the Discussion to state that the precise apical caspase mechanism linking mitochondrial ROS to caspase-3 remains to be defined (See revised Main Text page 19 line 22-25).

Response to Reviewers

Reviewer #1

The authors have addressed all of my concerns, and the manuscript is now suitable for publication.

Response: We sincerely thank the reviewer for the positive evaluation of our revised manuscript and for the constructive comments during the review process.

Reviewer #2

The author's revisions have addressed most of the issues I raised, the overall quality of the revised manuscript has improved significantly, and I recommend acceptance for publication.

Response: We sincerely thank the reviewer for the encouraging assessment and helpful suggestions. We are pleased that the reviewer finds the revised manuscript substantially improved.

Reviewer #3

The authors did a great job. The revision is thorough. The paper is much stronger now. I have a few remaining suggestions about wording and figure placement.

Comment 1: The data support immunogenic cell death, but total GSDME levels don't change—only its cleavage does. More importantly, GSDME is highly expressed in normal tissues and often plays protective roles there (see PMIDs: 41638192, 41407670, 40707439, 41701674). This means "abundance" does not equal "activation." FILM mainly acts on upstream metabolic thresholds (ROS and caspase-3 levels), not on GSDME itself. The authors should present GSDME as a "downstream executor." This neatly explains why giving fenofibrate systemically is safe in normal tissues—they don't have the FILM-induced metabolic stress. The above-

mentioned publications should be added into the references list for discussion.

Response: We thank the reviewer for this insightful suggestion. We have revised the Discussion to clarify that GSDME functions mainly as a downstream executor of pyroptotic immunogenicity, whereas FILM suppresses the upstream PPARα–PDK4–mitochondrial ROS–caspase-3 threshold required for GSDME cleavage. We have also added the suggested references discussing the tissue- and context-dependent functions of GSDME in normal tissues.

Comment 2: The paper focuses on FILM, but the key datasets that define it, especially the $CD45^+$ immune scRNA-seq and spatial transcriptomics, are stuck in the Supplementary Materials. For a journal like Nature Communications, these foundational data are critical for showing the study's impact. The authors should combine these panels and move them to the main figures, so readers can immediately see the multi-omics strength of the study.

Response: We thank the reviewer for this important suggestion. We have therefore moved the spatial transcriptomic analysis of CAFs, macrophages, NOS2 expression, pyroptosis activity and PPARα pathway activity into the main figures. We have also moved the $CD45^+$ immune-cell scRNA-seq analysis into the main figures to better show how PPARα agonist-induced GSDME-dependent pyroptosis remodels immune-cell states during anti-PD-1 therapy. To accommodate these additions, we moved several descriptive or repetitive phenotypic panels to the Supplementary Information.

Comment 3: The PDK4 kinase-dead rescue experiment (proving that kinase activity drives pyroptosis) and the Ppara-/- reciprocal transplants (proving the phenotype is tumor-intrinsic) are the most important mechanistic findings in the paper. But they are still hidden in Figures S16 and S18. These need to be in the main figures. To make room, move the repetitive or purely descriptive phenotypic panels to the supplement.

Response: We thank the reviewer for highlighting the importance of these mechanistic experiments. We have moved the PDK4 wild-type and kinase-dead rescue experiments into the

main figures to emphasize that PDK4 kinase activity is required for PPARα activation-induced mitochondrial ROS accumulation, caspase-3 activation and GSDME cleavage. We have also moved the Ppara−/− reciprocal transplantation experiment into the main figures to clarify that the therapeutic response is predominantly driven by tumor-cell-intrinsic PPARα. Several repetitive or descriptive phenotypic panels have been transferred to the Supplementary Information to keep the main figures focused.

Reviewer #4

Comment: The authors addressed all my concerns. I have no further suggestions.

Response: We sincerely thank the reviewer for the positive evaluation and for the helpful comments during the review process.
